# Unravelling the Secret of Sulfur Confinement and High Sulfur Utilization in Hybrid Sulfur‐Carbons

**DOI:** 10.1002/adma.202513346

**Published:** 2026-01-19

**Authors:** Tim Horner, Enis Oğuzhan Eren, Elif Begüm Yılmaz, Jiyong Kim, Ernesto Scoppola, Alexandros Vasileiadis, Nadezda V. Tarakina, Markus Antonietti, Paolo Giusto, Evgeny Senokos

**Affiliations:** ^1^ Department of Colloid Chemistry, Max Planck Institute of Colloids and Interfaces Potsdam Germany; ^2^ Functional Materials and Devices, Fraunhofer Institute For Applied Polymer Research IAP Potsdam Germany; ^3^ Department of Biomaterials, Max Planck Institute of Colloids and Interfaces Potsdam Germany; ^4^ Department of Radiation Science and Technology Delft University of Technology Delft The Netherlands

**Keywords:** inverse vulcanization, metal‐sulfur batteries, radical stabilization, sulfur confinement

## Abstract

Understanding sulfur confinement and chemical transformation in hybrid sulfur‐carbon materials is critical for advancing metal‐sulfur batteries. Here, we investigate the structural evolution of a sulfur‐rich polymer into a hybrid sulfur‐carbon via inverse vulcanization and thermal condensation. Multiscale analyses reveal a stepwise transformation, beginning with the emergence of sulfur radicals at ∼175°C, followed by the progressive development of a carbon matrix above 300°C that stabilizes the radical species. Around 450°C, a transitional phase forms, consisting of conjugated carbon clusters covalently bonded to sulfur chains. This hybrid structure confines sulfur within pseudo‐graphitic nanodomains, effectively suppressing polysulfide dissolution and enhancing redox stability. DFT simulations show how sulfur confinement modulates Na‐S reaction energetics, while electrochemical testing confirms high sulfur utilization, delivering ∼1000 mAh $g_{\mathrm{SC}}^{-1}$ and 1200 Wh ${\mathrm{kg}}_{\mathrm{SC}}^{-1}$, setting a new performance benchmark for room‐temperature Na─S batteries. These findings provide critical insights into the correlation between structural evolution and electrochemical performance, offering design principles for next‐generation sulfur‐based electrodes.

## Graphical abstract

Thermal condensation of inverse vulcanized sulfur‐carbon hybrids enables a bottom‐up sulfur confinement strategy, in which a protective carbon phase is progressively constructed around sulfur species. The resulting carbon nanodomains covalently tether sulfur chains and stabilize radical intermediates. This integrated architecture effectively suppresses polysulfide shuttling and enables nearly complete sulfur utilization, delivering ∼1000 mAh g^−1^ and 1200 Wh kg^−1^. The approach establishes a new performance benchmark for room‐temperature Na‐S batteries and offers clear design principles for advanced sulfur cathodes.

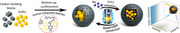

## 1 Introduction

Structuring of materials at the nanoscale, often perceived as a contemporary advancement, has historical origins spanning over four millennia [1]. This enduring pursuit, driven by a long‐standing fascination with manipulating matter at the nanoscale, has evolved into the interdisciplinary field of nanotechnology. One of the main focuses in this field is to explore the effect of confined environments on reaction mechanisms, kinetics, and product formation [2]. Confining reactants within nanoscale spaces, such as porous materials, capsules, or host‐guest systems, allows for the customization of molecular interactions, and chemical transformations [2, 3, 4, 5]. Comprehending and harnessing the effects of confinement presents significant opportunities for unlocking new avenues for catalysis, drug delivery, and energy storage, among others.

Porous materials serve as ideal structures for nanoscale confinement, acting as molecular vessels that create a protective environment to prevent undesired reactions [6, 7, 8]. By encapsulating molecules within their structure, porous materials isolate reactive or sensitive compounds, stabilizing otherwise unstable chemical species, and altering their reactivity, thereby broadening their potential applications. In particular, the confinement of sulfur species within nanocavities presents a promising approach for enhancing electrochemical sulfur conversion [9]. Sub‐nanometer pores can enclose metastable sulfur species, promoting quasi‐solid‐state reactions with improved stability and accelerated kinetics [10].

A widely used approach for confinement involves the creation of a host material, typically a conductive porous carbon, which is subsequently filled with sulfur through a melt‐diffusion or a gas‐infiltration method [11, 12, 13, 14, 15]. This approach allows sulfur to access and fill the open pores, but leaves a pathway for the potential reverse loss of polysulfides through these channels, leading to the critical issue of polysulfide shuttling [16, 17]. Additionally, this method faces several challenges, such as incomplete sulfur loading and uneven distribution within the matrix, along with the presence of electrically disconnected inactive species. Limited pore volume can further restrict sulfur loading, ultimately hindering the full utilization of the active species.

Recently, we proposed a novel synthetic strategy to form a microporous carbon matrix that enables the nanoconfinement of sulfur species via a one‐pot combined inverse vulcanization and thermal condensation process [9]. The encapsulation of sulfur within the carbon phase prevents the diffusion of electrolyte molecules into the confined space, resulting in a quasi‐solid‐state sulfur conversion. The physical entrapment of sulfur species in ultra‐micropores, along with their chemical anchoring, enables a reversible and atom‐efficient redox conversion, ensuring a nearly full sulfur utilization and long cycling stability [10]. However, the process by which this nanocomposite structure is formed remains elusive. Elucidating this mechanism could provide critical insights for the strategic design of materials optimized for sulfur confinement. This understanding may further facilitate the adaptation of these principles to other molecular systems by fine‐tuning structural and compositional features for improved performance and stability.

Herein, we report an investigation into the formation of the sulfur‐carbon hybrid materials synthesized via a one‐pot approach combining inverse vulcanization with thermal condensation by utilizing a more sustainable, naturally occurring terpene. By targeting different thermal condensation temperatures, the physicochemical structure of the resulting sulfur‐carbons can be tuned, significantly influencing their electrochemical performance. Employing these methods allows for the observation of structural changes in the sulfur‐carbon composites, which can be related to their performance as cathodes in room‐temperature sodium‐sulfur batteries. This approach provides valuable insight into the mechanisms driving the formation of these materials and highlights their potential impact on advancing energy storage technologies.

## 2 Main

**FIGURE 1:**
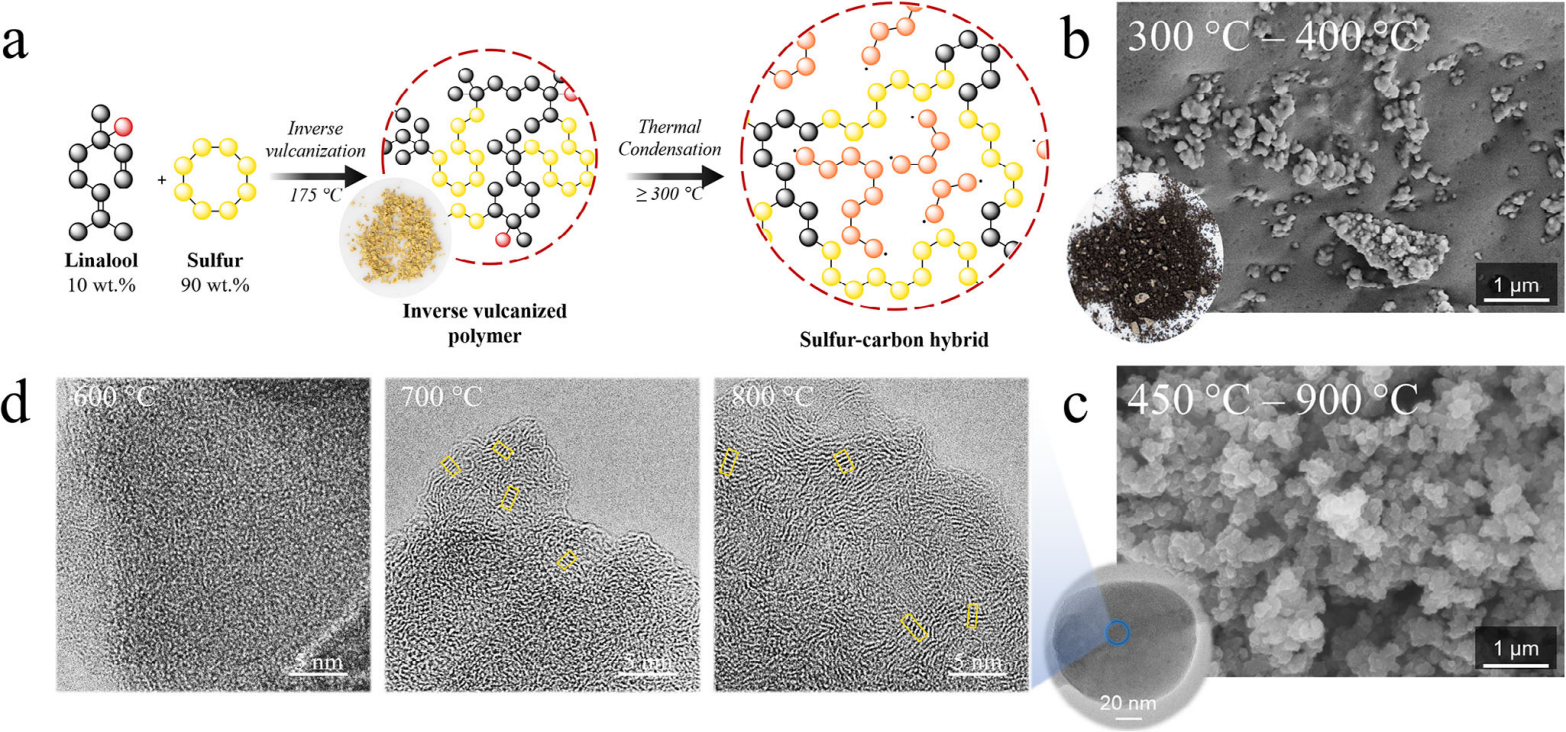
(a) Schematic illustration of the synthesis route for sulfur‐carbon hybrid materials via a one‐pot process combining inverse vulcanization at 175°C with subsequent thermal condensation at ≥ 300°C. SEM images of the sulfur‐carbons condensed in two temperature ranges: (b) 300°C to 400°C and (c) 450°C to 900°C, accompanied by an HR‐TEM image of a single sulfur‐carbon nanosphere. (d) HR‐TEM images of sulfur‐carbons thermally condensed at 600°C, 700°C, and 800°C.

The hybrid sulfur‐carbon material was synthesized through a one‐pot process combining inverse vulcanization and thermal condensation (Figure 1a). Elemental sulfur and linalool, a naturally derived terpene, were mixed in a 9:1 weight ratio and heated under a nitrogen atmosphere [9, 18]. Upon reaching 175°C, homolytic cleavage of the S─S bonds generates a highly reactive biradical sulfur solvent, which initiates an inverse vulcanization reaction with the olefinic C═C double bonds of linalool, covalently linking sulfur to carbon. In addition, side reactions such as hydrogen atom transfer contribute to the formation of a copolymer primarily consisting of linear and cyclic sulfur‐carbon motifs (Figure S1) [19, 20, 21, 22, 23]. The high sulfur‐to‐linalool ratio leads to an excess of non‐bonded sulfur that does not participate in the initial inverse vulcanization. This free sulfur acts as the main source of thermally generated sulfur biradicals, which can be stabilized within a confined carbon matrix during the following high‐temperature treatment.

The resulting sulfur copolymer was subsequently subjected to a thermal condensation step at higher temperatures ranging from 300°C to 900°C, forming black sulfur‐carbon hybrid monoliths (Figure 1b, inset). As the temperature increases toward 300°C, the sulfur biradicals engage in a dynamic interplay of chain propagation, fragmentation, and recombination. This leads to the formation of a transient equilibrium of short‐chain sulfur species, such as ∙S─S∙ and ∙S─S─S∙ biradicals. At these temperatures, in the absence of full confinement or covalent anchoring to carbon, a significant fraction of non‐bonded sulfur persists in these reactive radical forms. Beyond 300°C, the sulfur radicals begin to recombine or volatilize, while the surrounding carbon matrix undergoes condensation. This process is accompanied by a gradual decline in total sulfur content, particularly the non‐bonded fraction, which is continuously released upon surpassing sulfur's boiling point (445°C) [24, 25].

Elemental analysis revealed a progressive reduction of sulfur content with increasing thermal condensation temperatures, decreasing from 96.5 wt.% at 300°C (“300°C” referring to the thermal condensation temperature) to 44.6 wt.% at 600°C and 14.0 wt.% at 900°C (Table S1). The major loss of sulfur occurs as the thermal condensation temperature surpasses the boiling point of elemental sulfur, leading to the volatilization of remaining non‐bonded S_8_. In contrast, the observed increase in the carbon content is relative, as the absolute amount of carbon in the final product remains similar (14% to 19% carbon yield).

Electron microscopy observations of the sulfur‐carbons obtained at different thermal condensation temperatures show an evolution in the morphology of the material. Scanning electron microscopy (SEM) images reveal that all sulfur‐carbons primarily consist of large agglomerates ranging from 20 to 200 µm (Figure S2), composed of densely packed, submicron‐sized spherical particles. Elemental mapping via energy‐dispersive X‐ray spectroscopy (EDX) (Figures S3 and S4) shows a consistent and homogeneous distribution of carbon, oxygen, and sulfur throughout the sulfur‐carbons at the micron scale. At condensation temperatures below 450°C, the sulfur‐carbons develop a smooth glazed surface, suggestive of a softened molten sulfur phase condensed and solidified upon cooling (Figure 1b; Figure S2a,b). These characteristics vanish at 450°C, with the clusters of spherical nanoparticles becoming the dominant morphology (Figure 1c; Figure S2c–g). As the thermal condensation temperature approaches the boiling point of sulfur, the initially cohesive surface morphology becomes unstable due to the onset of sulfur volatilization. Elevated vapor pressure disrupts the softened sulfur layer, causing it to fragment and redistribute. The mobilized sulfur recondenses within the carbon phase into discrete nanospheres, with the carbon likely guiding and confining the process to favor the formation of spherical particles. Above 600°C, the nanosphere morphology remains mostly unaltered, while major changes in the structure of the sulfur‐carbon occur at the nanoscale.

High‐resolution transmission electron microscopy (HR‐TEM) reveals a predominantly amorphous carbon matrix for the sulfur‐carbon produced at 600°C. However, at 700°C, partial graphitization emerges, evident by the presence of pseudo‐graphitic carbon segments, consisting of a few carbon layers with large interlayer distances of ∼0.37 nm (Figure 1d; Figure S5). Simultaneously, high‐resolution scanning transmission electron microscopy (HR‐STEM) images reveal temperature‐dependent nanovoid formation (Figure S6), attributed to the decomposition and removal of sulfur confined within the carbon matrix. As the thermal condensation temperature reaches 800°C, graphitic domains grow in size, while the interlayer distance increases to 0.40 nm, reflecting ongoing sulfur volatilization. The gradual conversion of an amorphous to a pseudo‐graphitic system at higher temperatures is also evident from the changes in the sulfur L_2,3_‐ and carbon K‐edge electron energy loss (EELS) spectra (Figure S7). The decreasing intensity and broadening of sulfur‐related features indicate progressive sulfur loss, while the sharpening and growth of carbon π^*^ (∼286 eV) and σ^*^ (∼293 eV) peaks confirm increasing sp^2^ character and the development of an extended C─C network, characteristic of pseudo‐graphitic ordering.

This transformation marks a critical shift beyond 600°C, where the carbon matrix begins to adopt hard carbon‐like features with short‐range order, structural defects, and enhanced porosity [26, 27].

**FIGURE 2:**
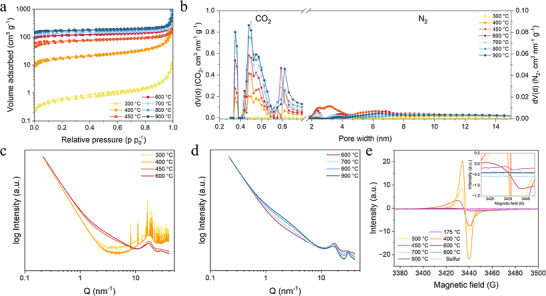
(a) N_2_ physisorption isotherm of sulfur‐carbons thermally condensed between 300–900°C. (b) PSD derived from CO_2_ and N_2_ physisorption. Normalized SAXS/WAXS scattering profiles of sulfur‐carbons condensed in two temperature ranges: (c) 300°C to 600°C and (d) 600°C to 900°C. (e) EPR spectra of elemental sulfur, inverse vulcanized sulfur‐linalool polymer (175°C), and sulfur‐carbons condensed between 300°C and 900°C.

Thermal condensation of the sulfur copolymer induces a profound evolution in the porosity of the resulting sulfur‐carbon composites. The formation of an optimal microporous network is crucial for the efficient containment of sulfur species. To investigate this transformation, gas physisorption analysis was further performed. Nitrogen adsorption isotherms exhibit type I curves with a steep uptake of nitrogen at low relative pressure, confirming a predominantly microporous character (Figure 2a) [28, 29]. As the condensation temperature increases from 300°C to 450°C, both the total specific surface area (SSA_tot_) and pore volume (V_tot_) increase significantly (Table S2 and Figure S8d), driven by the volatilization of non‐bonded sulfur species near and beyond sulfur's boiling point [24]. The continuous decomposition and subsequent evaporation of sulfur‐containing compounds at higher temperatures further contribute to the increase of SSA_tot_ and V_tot_. Complementary CO_2_ physisorption, which is sensitive to pores with a diameter smaller than 0.7 nm [30, 31], confirms the enrichment of ultra‐microporosity, in the sulfur‐carbon, reaching a peak at 900°C with an SSA_micro_ of 792 m^2^ g^−1^ and a V_micro_ of 0.25 cm^3^ g^−1^ (Table S2 and Figure S8a,e). The primarily ultra‐microporous nature of the sulfur‐carbons can be seen from the pore size distribution (PSD), with the majority of the pores being within the sub‐nanometer range (Figure 2b). This suggests that the temperature‐controlled evolution of sulfur not only generates a hierarchical porous network but also establishes a framework capable of physically trapping sulfur species.

Small‐ and wide‐angle X‐ray scattering (SAXS/WAXS) analyses provide complementary insights into the evolving nanostructure and porosity of sulfur‐carbons during thermal condensation (Figure 2c,d; Figure S9a–h) [32, 33, 34]. In the WAXS region, distinct diffraction peaks corresponding to crystalline sulfur appear between 8 and 50 nm^−1^, disappearing above 400°C, indicating the removal of volatile, non‐bonded S_8_ (Figure S9a) [35, 36]. A broad peak at 17.6 nm^−1^, attributed to the (002) diffraction of pseudo‐graphitic carbons, is observed even at low condensation temperatures, suggesting initial structural prearrangement in the otherwise amorphous matrix. As the condensation temperature increases, the (002) peak shifts to lower Q‐values, which is indicative of an expansion of the interlayer distance (Table S3). Simultaneously, the continuous removal of sulfur species competes with the development of larger pseudo‐graphitic domains. At 600°C, short‐range order becomes evident through the emergence of a peak at 30 nm^−1^, assigned to the (100) in‐plane ordering of graphene‐like domains [37]. As the temperature increases beyond 700°C, this peak becomes more pronounced, indicating the progressive development of pseudo‐graphitic domains, further supported by HRTEM observations (Figure 1d).

To analyze scattering data, different models were applied depending on the thermal condensation temperature. For sulfur‐carbons obtained at 300°C and 400°C, a combined model including Porod's law, the Teubner‐Strey model, and a constant background was used to interpret the scattering profile [32, 38, 39]. In this framework, Porod's law provides insights into macroscopic interfaces between carbon, sulfur, and void space while the Teubner–Strey model describes the scattering of thermally condensed sulfur‐carbons. At higher thermal condensation temperatures, additional scattering in the range from 0.6 to 4 nm^−1^, typically attributed to the micropore region, emerges [34]. In agreement with previous studies, a Q^−2^ slope and the Debye‐Bueche model were additionally incorporated to capture the onset of quasi‐2D lamellar‐like domains, indicating early structural reorganization toward layered carbon architectures [40]. This new composite model enables the extraction of key structural parameters, such as the lamellar d‐spacing, average micropore radius, and the amphiphilicity factor f_a_, tracking their evolution across the thermal condensation temperature range [32].

Based on these assumptions, the average micropores radius gradually increases with rising thermal condensation temperature, displaying a significant transition from 600°C to 700°C, where the radius expands from 0.47 to 0.74 nm (Table S3). This increase together with a rising f_a_ value, indicates a progressive loss of short‐range order and correlates with the depletion of sulfur with increasing thermal condensation temperature (Table S1). As volatile sulfur is removed, the resulting empty micropores enhance the electron density contrast, increasing scattering intensity in that region and possibly introducing local defects that disrupt the short‐range order.

In the initial stage of sulfur copolymer formation, thermal activation at 159°C initiates the homolytic cleavage of S─S bonds, generating sulfur biradicals [24, 41]. These highly reactive intermediates can either react with unsaturated carbon bonds or temporarily persist as free radicals in sulfur‐rich environments. However, due to their high reactivity and low intrinsic stability, these unpaired species typically undergo rapid recombination or quenching during synthesis and storage, which explains why they are often overlooked in sulfur cathode studies. Nevertheless, if stabilized, they could significantly influence the local chemical environment and impact the electrochemical behavior of sulfur cathodes. To investigate the formation and potential stabilization of sulfur radicals in our sulfur‐carbons, we employed electron paramagnetic resonance (EPR) spectroscopy, revealing a high density of unpaired electrons in the sulfur‐carbons thermally condensed between 300°C and 600°C (Figure 2e). A lack of a clear EPR signal in sulfur copolymer synthesized at 175°C indicates rapid recombination of biradicals due to insufficient chemical and structural stabilization. However, increasing the synthesis temperature after inverse vulcanization to 300°C initiates the formation of a complex carbon‐sulfur network, in which condensed chains form a semi‐organized matrix that restricts radical recombination and enables their detection at room temperature. The maximum EPR signal intensity is observed at 400°C, indicating that a significant fraction of sulfur radicals becomes sufficiently isolated and stabilized within the evolving sulfur‐carbon framework, effectively preventing their recombination into cyclic S_8_. Beyond this temperature, the signal intensity gradually decreases and disappears at 700°C due to ongoing thermal decomposition of sulfur species.

The determination of the g‐values from EPR spectra provides valuable insights into the local environment of unpaired electrons. In sulfur‐carbon materials thermally condensed at 300°C and 400°C, the g‐values are ∼2.005, which is notably higher than the free electron value of ∼2.002, indicating that the unpaired electrons are predominantly localized on sulfur atoms [42, 43]. These materials exhibit sharp, symmetric EPR signals, consistent with relatively uniform electronic environments around the sulfur species. However, increasing the thermal condensation temperature to 450°C results in a broader and more asymmetric EPR signal, accompanied by a further increase in the g‐value to 2.006. This spectral broadening and shift suggest the emergence of local hybridization within the developing carbon phase, which begins to influence the electronic environment of nearby radical species. Concurrently, thermal decomposition shortens the sulfur chains, further contributing to the observed spectral asymmetry [44]. At this stage, the unpaired electrons are likely no longer confined to individual sulfur atoms but instead delocalized across shorter sulfur chains embedded within the carbon matrix. The stabilization of these delocalized radicals can be facilitated by enhanced spin–orbit coupling within the hybrid structure, differing from materials like sulfurized polyacrylonitrile (SPAN), which exhibit lower g‐values associated with sulfur‐centered radicals stabilized via covalent C─S and N─S bonds in a conjugated polymeric framework [45]. Thermally condensed sulfur‐carbon hybrids, by comparison, display higher g‐values and broader EPR signals, suggesting more localized unpaired electrons that are progressively stabilized by a developing carbon matrix rather than by extended π‐conjugation [45, 46].

**FIGURE 3:**
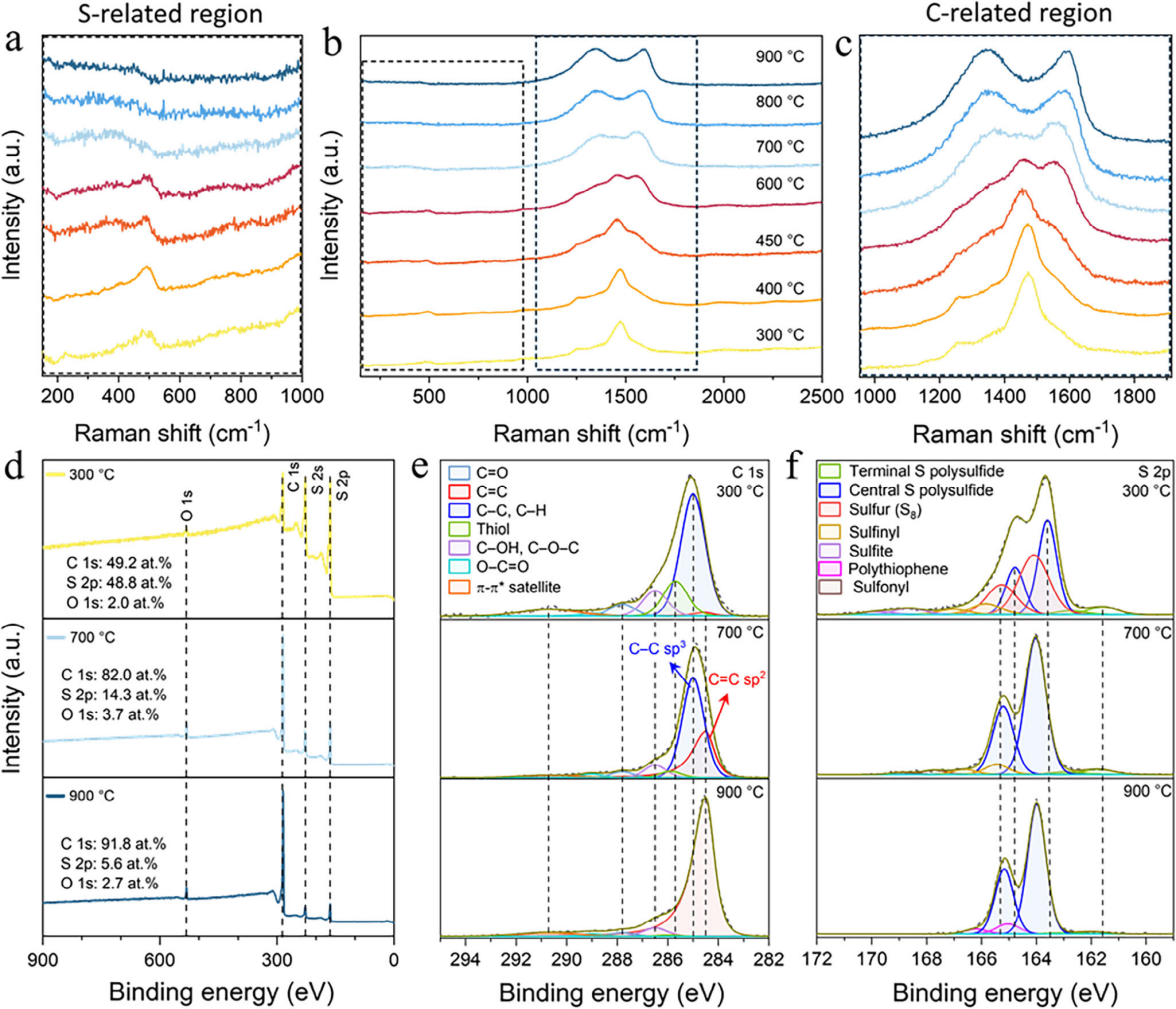
(a–c) Raman spectra of sulfur‐carbons thermally condensed between 300°C and 900°C, with highlighted spectral region of a) sulfur‐ and c) carbon‐related peaks. (d) XPS survey and high‐resolution (e) C 1s and (f) S 2p XPS peaks for sulfur‐carbons thermally condensed at 300°C, 700°C, and 900°C.

The analysis of the chemical evolution of sulfur‐carbons during thermal condensation was conducted using Raman spectroscopy (Figure 3a–c). The spectral region below 600 cm^−1^ reveals information about the chemical state of sulfur, with bands at 370 and 485 cm^−1^ corresponding to C─S bond stretching and S─S oscillation, respectively. Raman spectra recorded at low laser power and short exposure also confirm the presence of S_8_ at 300°C, as shown by distinct peaks below 600 cm^−1^ (Figure S10). These features quickly vanish under short exposure, due to the inherent instability of S_8_. The lack of Raman signals for S─S vibrational modes at elevated temperatures suggests that sulfur chains are confined within emerging pseudo‐graphitic carbon layers. This phenomenon, previously reported for nanoconfined sulfur species in sulfur‐carbon composites, was attributed to the phonon confinement effect [15, 47, 48, 49].

Significant changes in the Raman spectra are observed above 1200 cm^−^^1^, where a prominent peak at 1450 cm^−1^, associated with C─C and/or C─H bending vibrations in a disordered, partially condensed carbonaceous network modified or stabilized by sulfur, is present. This peak gradually decreases with increasing temperature, indicating progressive condensation and structural evolution of the sulfur‐carbon matrix [50]. A similar intermediate Raman band at 1450 cm^−1^ was previously reported for a sulfur‐cyclohexanol system heated to 400°C, where sulfur played a catalytic role in dehydration and dehydrogenation processes during low‐temperature graphene formation [51]. These intermediate species, distinct from graphitic carbon, were transient and disappeared above 450°C as a sp^2^‐bonded carbon network developed. A similar trend is observed in our sulfur‐carbons, for which the shape of the Raman spectrum changes drastically at 450°C, with the appearance of a shoulder at 1530 cm^−1^, which evolves into a distinct G band at higher temperatures. The G band corresponds to in‐plane stretching vibrations of sp^2^‐hybridized carbon atoms in a conjugated system. Simultaneously, the D band emerges at 1340 cm^−1^ and becomes prominent at 700°C, reflecting the breathing modes in the graphene Brillouin zone [52]. The gradual appearance of D and G bands, along with the fading of the peak at 1450 cm^−1^, indicates a shift from sp^3^‐hybridized C─C/C─H bond vibrations towards a more polyaromatic hydrocarbon system between 450°C and 700°C. Eventually, these nanostructures undergo partial graphitization from 700°C to 900°C, forming conjugated pseudo‐graphitic layers. The transformation of smaller polyaromatic domains into a more ordered pseudo‐graphitic structure is evident from the blueshift of the G band, indicating an increase in graphitic domain size and a reduction in structural disorder [53].

X‐ray photoelectron spectroscopy (XPS) analysis provides further insights into the chemical changes in the sulfur‐carbon structure (Figure 3d–f; Figure S11a–c, and Table S4). A clear increase in the C 1s signal alongside a gradual decline in the S 2p intensity reflects the transition from a sulfur‐rich environment to a carbon‐dominated system. High‐resolution deconvolution of the C 1s peak (Figure 3e; Figure S11b) reveals a clear transformation from a broad, sp^3^‐rich signal with contributions from C─C, C─O, and C─H bonds at 300°C, to a sharp, sp^2^‐dominated profile with an emerging *π*–*π* ^*^ satellite feature at 700°C and beyond. This development indicates progressive aromatization and local graphitic ordering of the carbon matrix, supported by the increasing sp^2^/sp^3^ ratio and consistent with the evolution of D and G bands in Raman spectra and the appearance of layered domains in HR‐TEM images [54, 55].

In parallel, the S 2p spectra (Figure 3f; Figure S11c) exhibit a complex peak structure at low temperatures, characteristic of central and terminal polysulfide environments, along with minor contributions from sulfur allotropes and oxidized species such as sulfonyls. [21, 56, 57] The S 2p components attributed to terminal polysulfide species likely correspond to the same sites responsible for the unpaired electrons observed in EPR (Figure 2e). As temperature increases, the S 2p features become narrower and shift slightly to higher binding energies, indicating both a reduction in sulfur chemical diversity and a more electron‐deficient, confined environment. These changes reflect the cleavage of long‐chain polysulfides and their conversion into shorter, chemically uniform sulfur species likely confined within or bonded to the developing graphitic carbon network (Figure S11c) [21, 22]. Despite the high temperature thermal condensation temperature of 900°C, a significant sulfur content remains, suggesting strong retention and possible incorporation of sulfur in thermally stable configurations, such as edge‐doped or thiophene‐like structures [58]. These structural and compositional trends support a synergistic transformation pathway in which sulfur promotes early condensation and crosslinking, while progressive carbonization stabilizes the system into an electronically delocalized, sulfur‐functionalized material.

**FIGURE 4:**
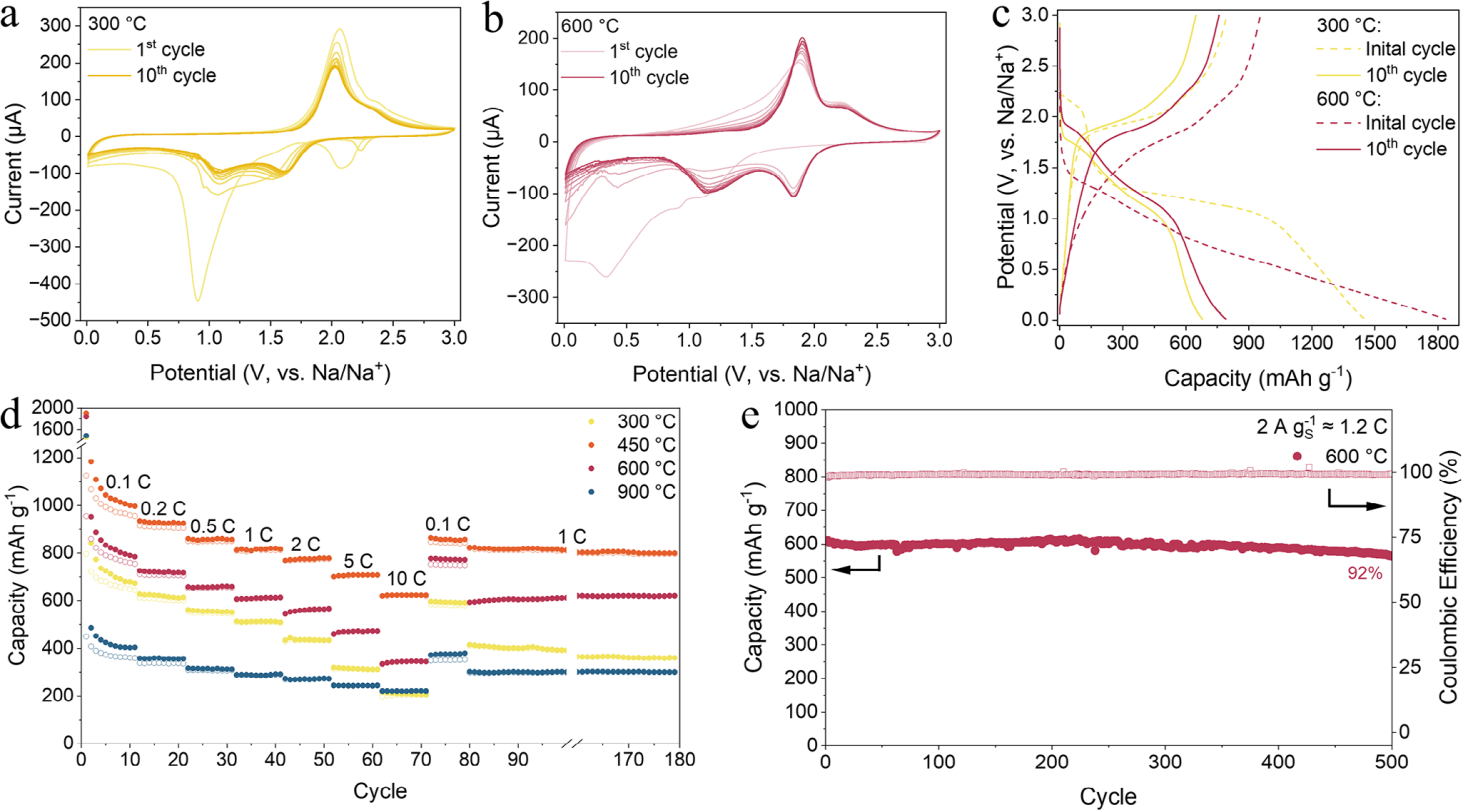
CV measurements of sulfur‐carbons thermally condensed at (a) 300°C and (b) 600°C. Comparison of GCD profiles of sulfur‐carbon condensed at 300°C and 600°C, showing the initial and 10^th^ cycle, (d) rate capability in the range of 0.1 C to 10 C and subsequent short‐term cycling at 1 C for 100 cycle, (e) long‐term cycling at 2 $A\;g_{S}^{-1}\;$(≈ 1.2 C) for sulfur‐carbon thermally condensed at 600°C.

Electrochemical analysis of the Na‐S system offers direct insight into how sulfur speciation and confinement evolve as a function of the thermal history of the sulfur‐carbon composite [59]. For the material condensed at 300°C, the initial cyclic voltammetry (CV) shows two distinct cathodic peaks at 2.1 and 0.9 V, associated with the stepwise reduction of elemental sulfur or long‐chain polysulfides (S_8_ → Na_2_S_x_, *x* ≥ 4) and the further conversion of short‐chain species (Na_2_S_2_ → Na_2_S), respectively (Figure 4a; Figure S12). Both peaks rapidly diminish in subsequent cycles, indicating that a large fraction of sulfur, likely in the form of non‐bonded and non‐confined S_8_, does not retain within the matrix. This behavior points to low sulfur stabilization, with soluble polysulfides diffusing into the electrolyte and undergoing irreversible side reactions [17, 60]. However, GCD profiles show that roughly half of the initial capacity is retained and stabilizes over extended cycling (Figure 4c; Figure S13). This suggests that while a portion of sulfur is leached early due to lack of effective confinement, a remaining fraction is chemically embedded or physically trapped within the sulfur‐carbon matrix, allowing it to participate in reversible redox reactions. For the sulfur‐carbon prepared at 400°C, an additional cathodic peak at 1.5 V appears during the first discharge (Figure S12), indicating the formation of intermediate polysulfides from medium‐length sulfur chains. The stabilized capacity reaches 1053 mAh g^−1^, showing a much smaller fraction of irreversible sulfur loss compared to the sulfur‐carbon condensed at 300°C, consistent with a lower content of unstable S_8_ (Figure 4c; Figure S14).

The electrochemical features of the sulfur‐carbons drastically change once the condensation temperature exceeds sulfur's boiling point. Starting from 450°C, no signs of sulfur reduction are observed during initial CV and GCD cycles (Figure 4b,c; Figures S12 and S13). Instead, the CV is dominated by a sharp cathodic feature below 0.5 V, attributed to electrolyte decomposition and formation of a solid‐electrolyte interface (SEI) on the surface of conductive carbon [61]. However, with continued cycling, two broad redox peaks emerge, indicating a gradual electrochemical activation of sulfur confined within the carbon network. The increase in peak intensity over the first 10 cycles, along with the shift in GCD plateau to higher potentials, confirms that sulfur is not initially accessible but becomes progressively active as Na‐ions infiltrate the matrix and cleave S─S bonds or open up embedded sulfur chains. This activation leads to a rise in the average potential of GCD curves and a stable energy density of 1200 and 933 ${\mathrm{Wh}\;\mathrm{kg}}_{\mathrm{SC}}^{-1}$ at 450°C and 600°C, respectively (Tables S6 and S7). Between 450°C and 700°C, the electrochemical signatures of the sulfur‐carbon composites shift noticeably, with the main GCD plateau and corresponding CV reduction peak moving from 1.6 to 1.85 V, reflecting the involvement of longer polysulfide species in the conversion process.

While low‐temperature samples initially contain substantially longer sulfur chains, these are largely lost during the first few cycles, leaving behind shorter and more stable species. In contrast, sulfur‐carbons treated above 450°C retain their long‐chain polysulfides, as stronger confinement within the carbon matrix enhances their chemical stability and suppresses shuttling. This remarkable stability arises from the effective confinement and protection provided by the carbon network at elevated thermal condensation temperatures, limiting solvent access and mitigating polysulfide shuttling. As the condensation temperature rises further, the average sulfur chain length shortens, evidenced by a decrease in plateau potential at 800°C and the complete disappearance of the high‐voltage plateau at 900°C. This shift reflects a critical reduction in local sulfur concentration, falling below the threshold needed to form extended polysulfides. Consequently, the electrochemical mechanism transitions to a direct reduction of tightly bonded sulfur to Na_2_S at 1.1 V, marking a fundamental change in redox behavior driven by sulfur confinement and depletion.

To gain further insight into the conversion mechanism, ex situ XPS was conducted on sulfur–carbons prepared at 300 and 600°C (Figure S15). The 300°C sample shows rapid precipitation of Na_2_S already at intermediate discharge, leaving residual sulfur and leading to incomplete utilization, whereas the 600°C material undergoes a more gradual S^0^ → Na_2_S_x_ → Na_2_S pathway with nearly full reversibility upon charging. These results confirm that stronger confinement at elevated condensation temperature promotes homogeneous sulfur dispersion, suppresses reorganization, and enables more stable redox chemistry. The rate performance of the sulfur‐carbons highlights the interplay between sulfur confinement, evolving carbon structure, and the redox kinetics (Figure 4d; Figure S14). Sulfur‐carbons prepared at 300°C and 400°C undergoes noticeable capacity decay at high C‐rates, retaining 49 and 21% at 5 C, respectively, primarily due to weak sulfur anchoring and prolonged polysulfide dissolution. Above 400°C, improved confinement and structural integration lead to higher rate stability, with 50%–70% capacity retention at 5 C. At higher thermal condensation temperatures, the partially sp^2^‐hybridized carbon matrix enables efficient electron delocalization, contributing to improved rate capability. However, the highest rate performance is achieved by the sulfur‐carbon thermally condensed at 450°C, containing relatively lower content of conductive carbon (Tables S1 and S4). This suggests that optimal rate tolerance is governed not merely through high electrical conductivity, but through a combined effect of a carbon matrix that enables both ion and electron transport and the presence of sulfur‐centered radicals that accelerate redox kinetics [62]. These radicals can act as highly reactive intermediates, lowering the energy barrier for S─S bond cleavage and facilitating the formation and transformation of polysulfide species during cycling. Their presence may also improve charge delocalization and promote faster electron transfer between sulfur species and the conductive carbon matrix, accelerating the overall sulfur reduction and oxidation processes. As a result, the material exhibits rapid and efficient redox dynamics, even under high‐rate conditions.

Electrochemical impedance spectroscopy (EIS) conducted at different potentials during both sodiation and desodiation shows a clear divergence between low and high‐temperature sulfur‐carbons (Figure S16a–c). Low temperature sulfur‐carbons exhibit pronounced impedance growth and additional interfacial processes during cycling between 1–3 V, consistent with poor sulfur confinement and nonuniform Na_2_S formation, whereas intermediate and high temperature sulfur‐carbons maintain low and only weakly varying impedance, consistent with finely dispersed polysulfides and Na_2_S (Figure S18a–d).

To estimate the stability of sulfur species during repeated electrochemical conversion, the electrodes were subjected to an additional 100 cycles at 1 C directly after the rate performance measurement (Figure 4d; Figure S14). The sulfur‐carbon condensed at 300°C exhibits a gradual capacity decay of 0.126% per cycle, consistent with low sulfur retention due to polysulfide dissolution. In contrast, sulfur‐carbons treated above 400°C demonstrate initial capacity increase and its full retention are observed, attributed to improved confinement of shortened sulfur chains within the carbon network. The effect of sulfur confinement becomes more prominent at 600°C, with a minor capacity decline of 0.016% per cycle over 500 GCD cycles (Figure 4e). These trends confirm that structural evolution of the carbon framework between 400 and 700°C plays a key role in stabilizing sulfur species and mitigating shuttle effects during extended cycling.

Post‐cycling EDX mapping further corroborates these findings (Figure S19a–c), while electrodes condensed at 300°C display aggregated sulfur‐rich clusters formed by dissolution and redistribution of non‐confined sulfur, those treated at higher temperatures retain a uniform sulfur distribution. This homogeneity reflects improved confinement and matrix integrity arising from progressive sp^3^‐sp^2^ rehybridization and local graphitization, explaining the enhanced reversibility and long‐term stability of high‐temperature sulfur‐carbons. In line with this, HR‐STEM analysis of the 600°C electrode after 500 GCD cycles reveals that the short‐range order of carbon nanodomains is largely preserved (Figure S20a–d), confirming that the partially graphitized framework remains intact and provides a robust host for stable sulfur redox chemistry.

To gain further insights into the kinetics of electrochemical processes in the sulfur‐carbons, a galvanostatic intermittent titration technique (GITT) was performed (Figures S21a and S22a). The GITT results reveal that the Na‐ion diffusion coefficient (D_Na_) increases with the condensation temperature of sulfur‐carbons, reaching its maximum at 800°C (Figures S21b,c, and S22b,c). This enhancement by more than an order of magnitude is attributed to two key factors: first, at condensation temperatures below 450°C, long sulfur chains create a polymer‐like environment, forming large clusters that clog pores and act as physical barriers, significantly hindering Na‐ion transport. As the temperature rises, these chains fragment into shorter polysulfide units, reducing steric hindrance and enabling more efficient Na‐ion diffusion. Second, higher condensation temperatures promote the formation of a more conductive carbon matrix with ordered domains, enhancing electrical conductivity and creating a nanoconfinement effect that disperses sulfur species more evenly. This structural evolution improves Na‐ion accessibility and minimizes diffusion limitations, allowing smaller sulfur species (S_4‐6_) to diffuse more freely within the carbon network. The combination of sulfur chain fragmentation and the development of a more conductive carbon structure synergistically facilitates Na‐ion transport, leading to a substantial increase in the diffusion coefficient.

**FIGURE 5:**
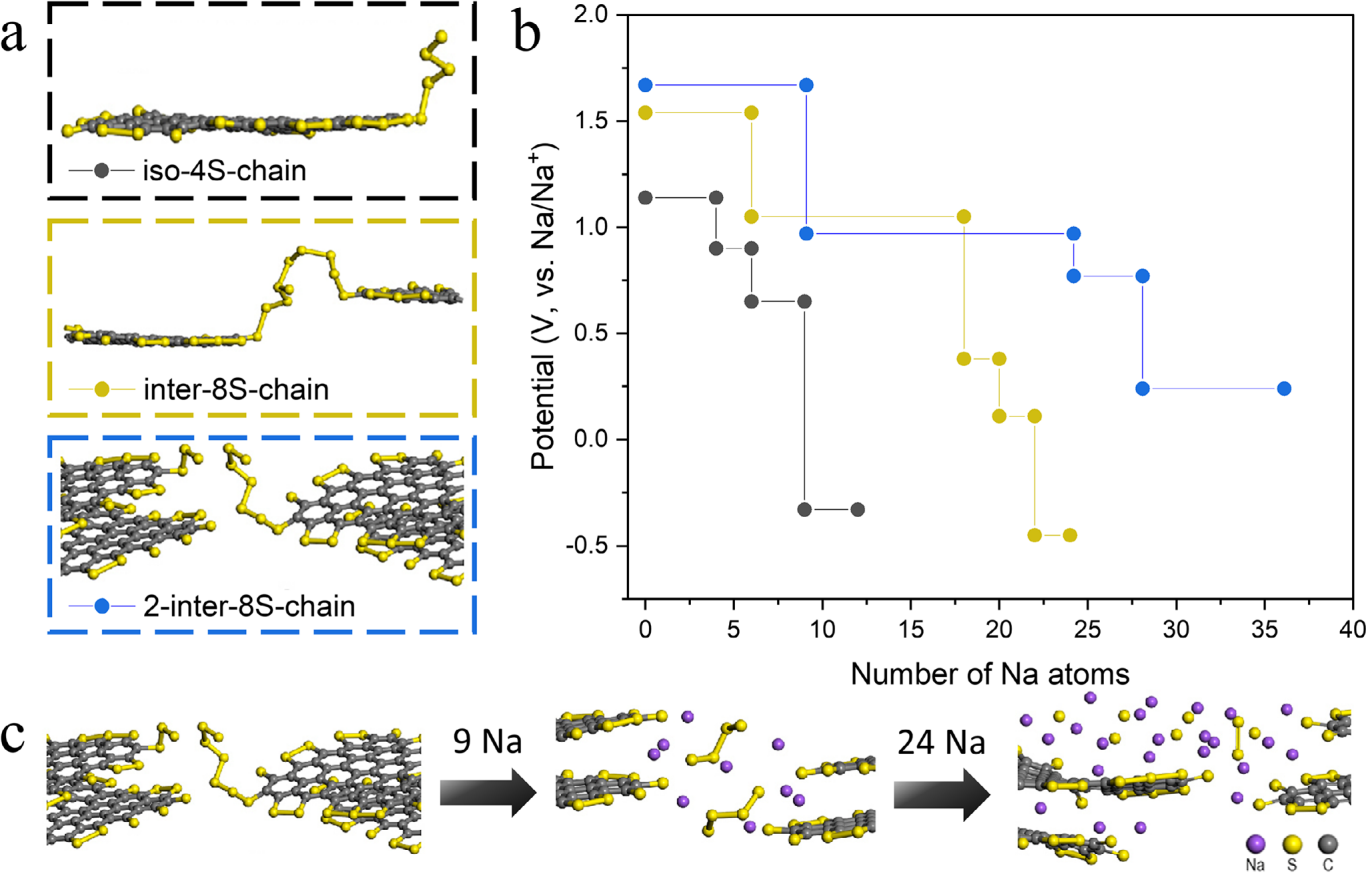
(a) DFT‐optimized structures of three sulfur‐carbons with different degrees of confinement and interactions between carbon sheets and sulfur chains: iso‐4S‐chain, inter‐8S‐chain, and 2‐inter‐8S‐chain. (b) Calculated voltage profiles upon sodiation of the three sulfur‐carbon models. (c) Sequential illustrations of Na‐atom insertion into the 2‐inter‐8S model and interaction with sulfur.

To investigate the effects of sulfur confinement and carbon structure on sodiation behavior, density functional theory (DFT) simulations were performed on defective model systems with varying sulfur chain lengths and degrees of spatial confinement (Figure 5a; Figures S23–S30, and Tables S8–S11). In the isolated sulfidated graphene model (Figure 5a, top), the sodiation process initiates as incoming Na‐atoms cleave the sulfur chains at ∼1.14 V (Figure 5b), resulting in the formation of localized Na‐S clusters (Figure S20a). Subsequent weaker interactions occur near edge‐bound sulfur sites at ∼0.65 V. As spatial confinement increases, first by introducing interacting sulfidated graphene sheets with longer sulfur chains between them (Figure 5a, middle), and then by adding additional sulfidated layers (Figure 5a, bottom), the resulting Na─S clusters become increasingly stabilized and shielded from the open environment. This confinement modulates the exposed surfaces of nucleated Na_x_S clusters and shifts sodiation plateaus to higher voltages, up to 1.72 V as sodium polysulfides are formed in more enclosed environments.

DFT calculations also suggest that Na‐atoms primarily react by cleaving free sulfur chains, leading to the formation of small Na_x_S clusters. This behavior correlates with the short, flat voltage plateaus observed during discharge, indicative of discrete redox steps. These findings are consistent with the redox features observed experimentally. The presence of longer sulfur chains extends the voltage plateaus, enabling greater Na atom incorporation and enhancing overall capacity. Figure 5c presents the structural evolution of the sulfur‐carbon composite upon sodiation, as observed in DFT simulations. The illustration reveals how Na atoms sequentially interact with sulfur chains and carbon edge sites, undergoing distinct reduction stages and forming various Na_x_S species. The full computational analyses, detailing the impact of sulfur chain length and confinement, is provided in the (Figures S23–S30). These results demonstrate that both parameters significantly affect redox behavior by stabilizing Na‐S intermediates and modulating sodiation energetics, providing valuable mechanistic insight into the behavior of complex sulfur‐carbon architectures in Na‐S batteries.

**FIGURE 6:**
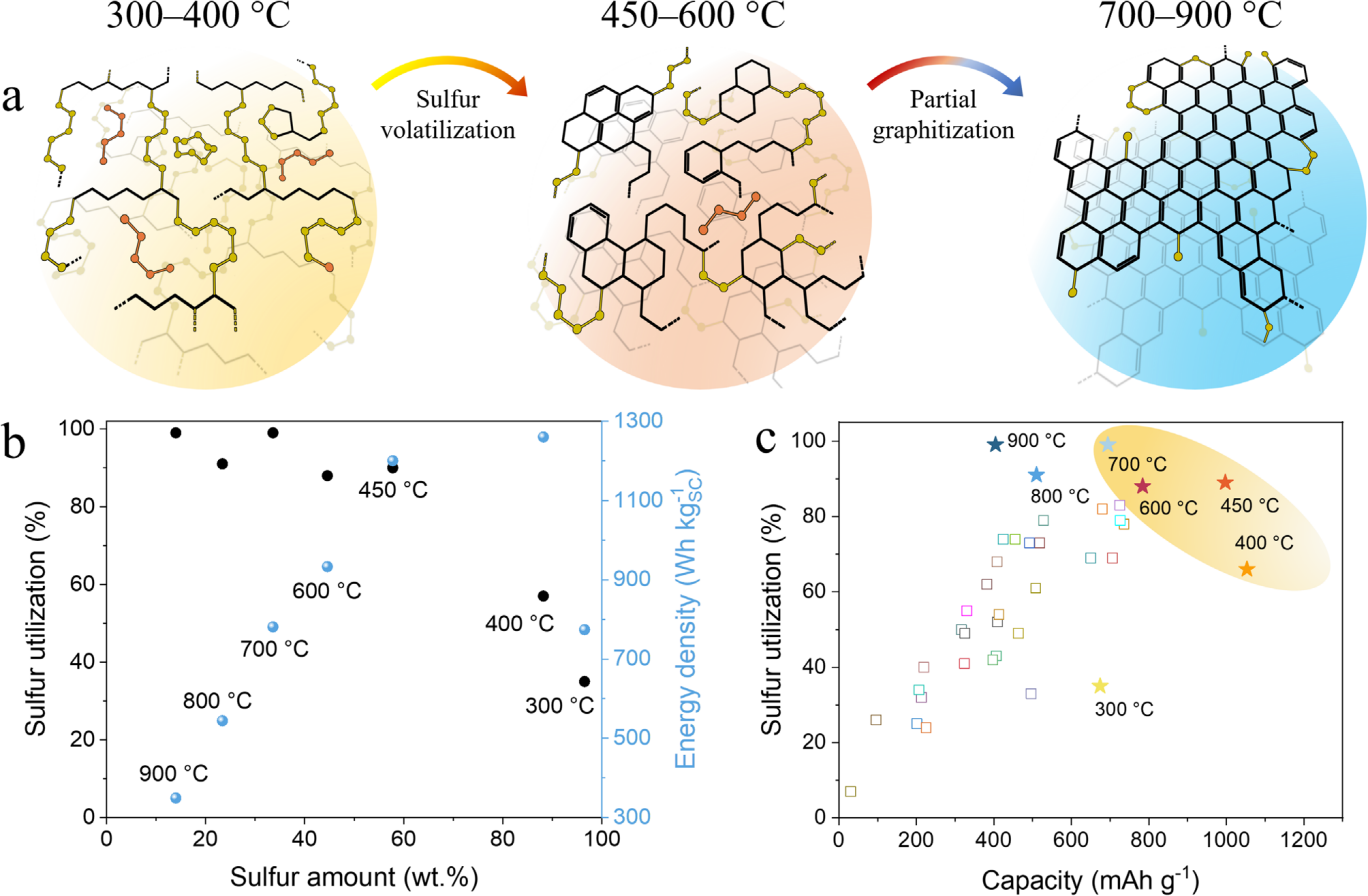
(a) Schematic illustration of the structural evolution of sulfur‐carbons during thermal condensation with increasing temperatures. Carbon is shown in black, covalently bonded sulfur in yellow, and sulfur radicals in red. (b) Correlation between sulfur content and sulfur utilization in the resulting materials. Corresponding energy densities (based on total mass of sulfur‐carbon) are indicated on the secondary *y*‐axis. (c) Comparison of sulfur utilization and specific capacity of the sulfur‐carbon materials developed in this study with previously reported cathode materials for room‐temperature Na─S batteries from the literature.

As revealed through extensive physicochemical and electrochemical analyses, the sulfur‐carbon undergoes distinct structural transformations across three temperature regimes (Figure 6a). Below 450°C, the sulfur‐carbons retain a high sulfur content (>85 wt.%), primarily as long‐chain sulfur species (S_x_, *x* ≥ 5) covalently bonded to a soft carbon matrix composed of sp^3^‐hybridized carbon chains, resembling polyisoprene‐like structures. At this stage, an excess of non‐bonded sulfur biradicals are generated, some are stabilized by the surrounding carbon matrix and persist at room temperature, while others recombine to form S_8_ molecules. In the intermediate temperature range from 450°C to 600°C, the sulfur content decreases markedly (58–45 wt.%) due to the gradual removal of non‐bonded S_8_ and shortening of the sulfur chain lengths. Concurrently, the carbon framework begins transitioning from sp^3^ to sp^2^ hybridization, forming partially conjugated and locally graphitized domains. At condensation temperatures above 700°C, the carbon matrix becomes predominantly sp^2^‐hybridized, forming a pseudo‐graphitic matrix, while the sulfur content falls below 25 wt.%, S_1‐2_ species covalently attached to the carbon backbone.

A key property of metal‐sulfur conversion systems is the amount of sulfur that is actively participating in the reaction relative to the total amount of sulfur incorporated in the material. This number is usually around 50%–60% and rarely approaches 80% due to various factors, such as low electrical conductivity and potential losses of active material through polysulfide shuttling or formation of inactive sulfur species. The amount of sulfur involved in the conversion reaction can be determined by analyzing the contribution of carbon to the total capacity. Linear GCD profiles below 1.25 V, excluding sulfur conversion charge, as confirmed by DFT calculations, show a capacity increase from 68 to 130 mAh g^−1^ as the condensation temperature rises (Figure S31). This increase is due to the gradual formation of defect‐rich conjugated carbon domains, providing additional conductive sites for Na‐ion adsorption. Sulfur utilization can be estimated by excluding the carbon contribution to total capacity (Table S6). For sulfur‐carbon composites synthesized at lower temperatures, utilization remained below 65%, largely due to substantial sulfur loss from polysulfide shuttling during initial cycles. As the condensation temperature increases, the sulfur‐carbon composite develops a structured and conductive carbon matrix, leading to effective sulfur confinement and a sharp improvement in utilization, peaking at 99% for the composite treated at 700°C.

The combination of high sulfur utilization and quasi‐solid‐state mechanism of sulfur conversion in a confined space directly impacts the energy density, influenced by the average potential, total capacity, and the sulfur loading (Figure 6b; Tables S6,  S7, and S12). The sulfur‐carbons formed between 400°C and 600°C feature a favorable balance of high sulfur content, stable radicals, and effective carbon encapsulation, delivering high energy densities of up to 1200 ${\mathrm{Wh}\;\mathrm{kg}}_{\mathrm{SC}}^{-1}$. At lower temperatures, a large fraction of sulfur remains in an unconfined elemental state, prone to dissolution and shuttling in the electrolyte, restricting the overall energy density to 774 ${\mathrm{Wh}\;\mathrm{kg}}_{\mathrm{SC}}^{-1}$. In contrast, partial graphitization of the sulfur‐carbon formed at 700°C enhances electronic conductivity and improves sulfur confinement within the carbon matrix, enabling near‐full sulfur utilization. However, despite these advantages, the lower sulfur content limits the energy density to ∼780 ${\mathrm{Wh}\;\mathrm{kg}}_{\mathrm{SC}}^{-1}$.

Thermally condensed sulfur‐carbon hybrids derived from inverse vulcanized copolymers establish a new benchmark for RT Na─S cathodes, achieving a total capacity of ~1000 mAh g^−1^ and nearly complete sulfur utilization (Figure 6c; Table S12), and these values are largely preserved even at higher sulfur loadings (Figure S33). This performance arises from a tailored architecture that combines high sulfur loading with covalently bonded chains and radical species uniformly confined within a conductive carbon matrix. The structure facilitates efficient electron transport while suppressing polysulfide shuttling, minimizing active material loss, and enabling extended cycling stability. These properties result from a finely tuned balance between sulfur chain length, reactivity, and spatial confinement, which promotes stable sulfur‐carbon interactions. Together, the synergistic effects of sulfur confinement, structural stability, and electronic connectivity position this material among the most promising cathodes for next‐generation room‐temperature Na‐S batteries.

## 3 Conclusion

In this study, we investigated the chemical and structural development of the hybrid sulfur‐carbon structure synthesized through a combined one‐pot inverse vulcanization/thermal condensation approach within 300°C to 900°C. At lower condensation temperatures (300°C to 400°C) the sulfur‐carbon contains more than 90 wt.% of sulfur in the form of long polysulfide chains interconnected through short sp^3^‐hybridized carbon chains and non‐bonded elemental sulfur. If the condensation temperature is increased to intermediate temperatures (450°C to 700°C) the non‐bonded S_8_ evaporates, resulting in the formation of the majority of the porosity of the sulfur‐carbon. Additionally, as the temperature is increased, the carbon chains condense into a mixture of sp^2^/sp^3^‐hybridized carbon matrix. The sulfur retained in the sulfur‐carbons is partially confined, allowing for the stabilization of sulfur radicals, which lead towards increased rate capability and utilization. Through the combination of a moderate sulfur content of up to 58.5 wt.%, polysulfide chain length, partially condensed carbon matrix, and ultra‐microporosity, the sulfur‐carbons condensed at 450°C exhibit a highly stable capacity of almost 1000 ${\mathrm{mAh}\;g}_{\mathrm{SC}}^{-1}$ and an energy density of 1200 ${\mathrm{Wh}\;\mathrm{kg}}_{\mathrm{SC}}^{-1}$. Further increasing the condensation temperature leads to the development of a pseudo‐graphitic carbon matrix and shortening of the polysulfide chains, which results in the formation of electrochemically inactive sulfur species, thereby reducing the capacity and energy density. This comprehensive study highlights the influence of thermal condensation temperature on the structural and electrochemical properties of sulfur‐carbon materials and provides a pathway for next‐generation Na─S batteries.

## 4 Experimental

### 4.1 Inverse Vulcanization/Carbonization of Linalool With Sulfur

The sulfur‐carbons were synthesized via the following procedure: [9] Sulfur (thermo scientific, 99.5%) and linalool (Sigma–Aldrich, ≥99%) were placed in a ceramic crucible (diameter: 11 cm) in a ratio of 90:10 wt.% and introduced into a muffle furnace (Nabertherm, Germany) with gas chamber which was flushed for 2 h with nitrogen (1.2 L min^−1^) before heating. The closed crucible was then heated via two steps to the chosen thermal condensation temperature. The first heating ramp started at room temperature, rising to 175°C (180°C h^−1^) and kept at that temperature for 20 min before increasing it to the chosen thermal condensation temperature (300–900°C, 180°C h^−1^) and kept there at that temperature for 2 h. Throughout the heating program, the chamber is continuously supplied with nitrogen to maintain an inert atmosphere. The resulting sulfur‐carbons were denoted according to the temperature of the thermal condensation step (e.g., 300°C). For electrode preparation, the resulting sulfur‐carbon was ground to a powder.

### 4.2 Electrode Preparation and Electrochemical Measurements

All given potentials are measured vs. Na/Na^+,^ and all capacities are normalized by the total mass of the active material. The working electrodes were fabricated by blending sulfur‐carbon acting as active material with conductive carbon black (Ketjenblack, AkzoNobel) and acrylonitrile multi‐copolymer (LA133, redoxme AB, 15 wt.% in H_2_O) as the binding agent in a weight ratio of 70:20:10. For working electrodes containing active material prepared at 450°C, sodium alginate (Sigma–Aldrich) was used as binder in the same ratio. Both binders were dissolved in bidestilled water (Integra UV plus) before the addition of the active material conductive carbon blend. The mixture was applied onto a carbon‐coated aluminum (thickness: 15 µm) using an automatic doctor blade film applicator (mtv messtechnik, Germany) and dried overnight in a vacuum oven at 60°C. Afterwards, the electrodes were stamped into circular disks and employed as working electrodes by assembling them in a three‐electrode Swagelok‐type cell in an Ar‐filled glovebox (MBraun, Germany) with an H_2_O and O_2_ content of less than 0.1 ppm. The final mass of the active materials in electrodes was ∼1.0 mg cm^−2^ for all sulfur‐carbons. The Swagelok‐type cell was structured by wetting the working electrode with the electrolyte (1 m NaClO_4_ in 50:50 ethylene‐, propylene carbonate with 5 wt.% fluoroethylene carbonate, E‐Lyte GmbH), placing a circular piece of glass fiber (Whatman GF/C, diameter: 13 mm) and stacking a thin circular slice of sodium metal (99.5%, Sigma–Aldrich, 99% Thermo Scientific Chemicals) as counter electrode. Sodium metal was also employed as the reference electrode separated by one or two circular pieces of glass fiber, which were wetted each with the same electrolyte. For each glass fiber placed in the battery 100 µL of electrolyte was added, resulting in a total of 200 to 300 µL of electrolyte per Swagelok‐cell. Electrochemical measurements were conducted on a Biologic MPG‐2 instrument (France). The galvanostatic charge–discharge curves of the half‐cells were collected in a potential range of 0.01–3.00 V and a current of 0.1 C if not stated otherwise, with half‐cells resting for three to six hours before the measurements. Cyclic voltammetry analysis was conducted in a potential range of 0.01–3.00 V at a scan rate of 0.1 mV s^−1^ unless stated otherwise. The rate capability of the sulfur‐carbons was evaluated at seven different C rates: 0.1, 0.2, 0.5, 1, 2, 5, and 10 C, with 10 cycles at each rate before returning to 0.1 C for another 10 cycles. The cyclability of all sulfur‐carbons was assessed with short‐term cycling at 1 C for 100 cycles. In addition, the long‐term performance of the sulfur‐carbons thermally condensed at 600°C was tested for 500 cycles at 1 $A\;g_{S}^{-1}$. The galvanostatic intermittent titration technique was conducted, where current pulses (0.2 C) were applied for 900 s, and relaxation potentials were measured for 1800 s. Calculation of the Na‐ion coefficients from these measurements was achieved based on the approximation from Weppner and Huggins [63, 64, 65]. This model requires a significantly larger relaxation than diffusion time additional to low contributions of ohmic and kinetic overpotential achieved through large transient data. The carbon contribution to the total capacity of the sulfur‐carbons was estimated using galvanostatic charge–discharge measurements with stepwise increasing low cut‐off potentials. These potentials were raised in 0.25 V steps, and the discharge capacity was determined from the last cycle before plateaus associated with sulfur conversion were observed. Electrochemical impedance spectra (EIS) were obtained in the frequency range from 100 kHz to 100 mHz at a potential amplitude of 10 mV and varied bias voltage at the indicated states of charge.

### 4.3 Physicochemical Characterizations

SAXS/WAXS measurements were conducted on the µSpot beamline of BESSY‐II (Helmholtz‐Zentrum Berlin, HZB, Germany) [66]. Experiments were performed using a monochromatic X‐ray beam at approximately 18.0 keV and a beam size of approximately 100 µm width obtained by a sequence of pinholes. The scattered intensities were collected with a Dectris Eiger 9 m detector. The scattering Q‐range was calibrated against quartz powder. The collected data was normalized over primary beam intensity, transmission, and standard glassy carbon [34]. At 300°C and 400°C the measured data were modeled using a combination of a constant background term, a Q^−4^ power law and the Teubner‐Strey model, with the addition of a Q^−2^ power law and the Debye‐Bueche model at higher thermal condensation temperatures (Equation 1) [32, 38, 39, 67].

(1) $$\begin{array}{cc} I_{total}^{cm-1}\left(Q\right) & =I_{Porod}^{cm-1}\left(Q\right)+I_{PL}^{cm-1}\left(Q\right)+I_{DB}^{cm-1}\left(Q\right)+I_{TS}^{cm-1}\left(Q\right)\\ & \;+background \end{array}$$

Porod's and Q^−2^ power law are proportional to the scattered intensity with a Q^−4^ and Q^−2^ slope, respectively.

(2) $$I_{Porod}^{cm-1}\left(Q\right)\propto \frac{1}{Q^{4}}$$

(3) $$I_{PL}^{cm-1}\propto \frac{1}{Q^{2}}$$

The Teubner‐Strey model (Equation 4) is then further utilized to calculate the amphiphilicity factor f_a_ acting in this system as a disorder parameter indicating a certain degree of short‐range in the case of f_a_ < 0 [32].

(4) $$\begin{array}{cc} I_{TS}^{cm^{-1}}\left(Q\right) & =8\pi \phi \left(1-\phi \right){\left(\Delta SLD\right)}^{2}\\ & \;\times \frac{\xi^{3}}{{\left(1+{\left(\frac{2\pi \xi }{d}\right)}^{2}\right)}^{2}+{\left(1-{\left(\frac{2\pi \xi }{d}\right)}^{2}\right)}^{2}\xi^{2}Q^{2}+\xi^{4}Q^{4}}\; \end{array}$$

(5) $$f_{a}=\frac{1-{\left(\frac{2\pi \xi }{d}\right)}^{2}}{1+{\left(\frac{2\pi \xi }{d}\right)}^{2}}$$

When ξ ≪ *d*, the system exhibits a high degree of disorder, indicating a predominantly heterogeneous structure. In this limit, the Teubner‐Strey model progressively converges toward the Debye‐Bueche model for random phase mixtures (Equation 6) [32].

(6) $$I_{DB}\left(Q\right)=8\pi \phi \left(1-\phi \right){\left(\Delta SLD\right)}^{2}\frac{\xi^{3}}{{\left(1+\xi^{2}Q^{2}\right)}^{2}}$$

In which ϕ and ϕ(1 − ϕ) described the volumetric fractions of a two‐phase system, ΔSLD is the scattering length density difference between, ξ is the correlation length limiting the occurrence of order in the system, d is the pore‐pore distance.

The chemical composition and chemical states were obtained with an Axis Supra+ (Kratos Analytical, UK) X‐ray photoelectron spectroscopy (XPS) setup using monochromatised Al Kα radiation for excitation (15 kV, typical 20 mA). CasaXPS software was used for data processing and interpretation; XPS signals were fitted using GL (30) line shapes, combining Gaussian (70%) and Lorentzian (30%) line shapes, and an asymmetric Lorentzian line shape (LA(1.2,2.5,5)) [68, 69]. The binding energies of C─C bonds, mainly attributed to sp^3^ carbons, in all sulfur‐carbons from 300°C to 900°C were charge‐corrected by setting them equal to 285.0 eV, and their oxygen‐bound specious of C─O, C═O, O─C = O, and were equally fitted at 286.5, 287.8, and 289.0 eV, respectively [68, 70, 71]. Meanwhile, the C = C peaks, mainly attributed to pseudo‐graphitic sp^2^ carbons, and *π*–*π* ^*^ shake‐up satellite are well‐fitted with their aliphatic (C─C, C─H) and their oxygen‐bound peaks in all sulfur‐carbons from 300 to 900°C at 284.5 and 291.0 eV, respectively [68, 72]. The XPS peak‐fitting parameters for C 1s and S 2p are summarized in Tables S13 and S14, respectively.

For ex situ XPS analysis, the electrodes were first discharged to their respective potentials, followed by the application of a constant voltage hold for 30 min. The cells were then disassembled in an Ar‐filled glovebox (H_2_O and O_2_< 0.1 ppm), and the electrodes were carefully rinsed with dry diethyl carbonate. After rinsing, the electrodes were dried within the glovebox at 60°C for 3 h. The prepared samples were subsequently mounted on the XPS sample stage and transferred to the instrument via a double‐sealed transport box before introduction into the evacuation chamber.

Raman spectroscopy was obtained using a WITec Alpha 300 (Germany) confocal Raman microscope with an excitation wavelength of 532 nm, a laser power of 0.5 mW, and an acquisition time of 10 s.

Electron paramagnetic resonance (EPR) measurements were performed on a Bruker EMXnano benchtop X‐Band EPR spectrometer (Germany) with the following settings: Center Field 3448 G, Sweep Width 200 G, Receiver Gain 40 dB, Modulation Amplitude 1.000 G, Number of Scans 20, and Microwave Attenuation 25 dB. Sulfur‐carbons were placed in flame‐sealed EPR capillaries (IntraMark, volume 50 µL, ID 0.86 mm) within an EPR tube (ID 3 mm, OD 4 mm, length 250 mm). The *g*‐values were evaluated using Xenon 1.6 (Bruker) software.

Gas physisorption measurements using CO_2_ at 273 K and N_2_ at 77 K were performed on a Quantachrome Quadrasorb SI (Austria), while Ar physisorption measurements at 87 K were performed on a Quantachrome Autosorb IQ. Prior to the gas physisorption measurements, the sulfur‐carbons with a thermal condensation temperature below 450°C were degassed at room temperature for 18 h, whereas for those with a condensation temperature at or exceeding 450°C, degassing is carried out at 150°C for the same duration. The isotherms obtained from Ar and N_2_ physisorption were analyzed using the Brunauer–Emmett–Teller (BET) method and quenched‐solid density functional theory (QSDFT) to calculate total specific surface area (SSA_tot_), total pore volume (V_tot_), and pore size distribution (PSD) [73, 74, 75, 76, 77]. The isotherms obtained from CO_2_ physisorption measurements were analyzed using a non‐local density functional theory (NLDFT) [78].

Elemental analysis (C, H, N, S, O) was performed using EA3000 and EA3100 elemental analyzers (EuroVector Srl, Pavia, Italy). For C, H, N, and S determination, samples were quantitatively combusted in an oxygen‐rich atmosphere, while oxygen analysis was conducted via high‐temperature pyrolysis. The resulting gaseous products (CO_2_, H_2_O, N_2_, SO_2,_ and CO) were separated on a chromatographic column and detected by a thermal conductivity detector (TCD). Quantification was achieved by calibration against certified standards, sulfanilic acid, 2,5‐bis(5‐*tert*‐butyl‐benzoxazol‐2‐yl)thiophene, thiourea, and elemental sulfur for C, H, N, and S. Benzoic acid and acetanilide was used for O. For samples with sulfur contents above 10 wt.%, 5 to 10 mg of V_2_O_5_ were added as a combustion catalyst to ensure complete oxidation.

Scanning Electron Microscopy (SEM) imaging was performed using the Zeiss LEO 1550‐Gemini (Germany) operated at acceleration voltages of 3, 5, and 10 kV, and equipped with an Oxford Instruments X‐MAX (UK) 80 mm^2^ energy‐dispersive X‐ray (EDX) detector. For (scanning) transmission electron microscopy observations, a suspension of the sample in ethanol was sonicated for 10 min and then drop‐cast to a Cu grid with a holey carbon support and dried for 30 min. The (S)TEM study was performed using a double Cs corrected JEOL JEM‐ARM200F (S)TEM operated at 80 kV and equipped with a cold‐field emission gun and a Gatan Quantum GIF spectroscopy system. Annular dark‐field scanning transmission electron microscopy (ADF‐STEM) images were collected at a probe convergence semi‐angle of 25 mrad. Electron energy loss spectroscopy data were collected in dual EELS mode at energy dispersions 0.1 and 0.25 eV ch^−1^, allowing correction for the zero‐loss peak position. The power law was used build the model for the background subtraction. Multiple scattering effects have been removed using the Fourier ratio method, implemented in the Gatan Digital Micrograph software.

### 4.4 Computational Parameters

The simulated structures utilized density functional theory (DFT) computations with the Perdew‐Burke‐Ernzerhof functional within the Vienna Ab initio Simulation Package framework [79, 80]. Projector augmented wave potentials with [Ne] cores for sodium and sulfur, and [He] cores for carbon, were employed [81]. All computations were spin‐polarized. For DFT relaxations, a cutoff energy of 400 eV was applied along with a 2x2x1 k‐point grid, incorporating dispersion force corrections [82]. Additionally, ab initio molecular dynamics (AIMD) simulations were performed in the canonical ensemble, controlled by the Nosé‐Hoover thermostat, using a 300 eV cutoff energy and a gamma‐only k‐point mesh [83, 84].

Sulfidated graphene models were designed similarly to the pure carbon configurations explored in previous studies [85, 86]. The geometry of the structures, along with the periodic boundary conditions of each model, are presented in Figure S17. The models were optimized by performing AIMD simulations for 10 ps. Then, sodium was added to the models at various concentrations, and AIMD simulations were performed again so that sodium could relax in preferred positions. After AIMD simulations, DFT relaxations and energy calculations were performed to obtain the enthalpies of formation and voltage [87, 88]. Having the MD‐optimized structures as starting points allowed us to additionally remove and insert sodium, examining a collection of additional competitive configurations to complement the enthalpy investigation. The rationale for using sulfidated graphene sheets was based on an additional investigation presented in Figures S17–S20 showcasing how S interacts with defective graphene.

## Conflicts of Interest

The authors declare no conflict of interest.

## Supporting information




**Supporting File**: adma72111‐sup‐0001‐SuppMat.docx

## Data Availability

The data that support the findings of this study are available from the corresponding author upon reasonable request.
