# Supplementary material for: Unravelling the Secret of Sulfur Confinement and High Sulfur Utilization in Hybrid Sulfur‐Carbons
Source: Adv Mater. 2026 Jan 19;38(34):e13346. doi: 10.1002/adma.202513346 (PMC13274698; doi:10.1002/adma.202513346)
Supplement: Supplementary file 1 — Supporting File: adma72111‐sup‐0001‐SuppMat.docx [file ADMA-38-e13346-s001.docx]

Unravelling the Secret of Sulfur Confinement and High Sulfur Utilization in Hybrid Sulfur-Carbons

*Supporting Information*

*Tim Horner,^a^ Enis Oğuzhan Eren,^a^ Elif Begüm Yılmaz,^a^ Jiyong Kim,^b^ Ernesto Scoppola,^c^ Alexandros Vasileiadis,^d^ Nadezda V. Tarakina,^a^ Markus Antonietti,^a^ Paolo Giusto^a^ and Evgeny Senokos^a^**

*^a^ Department of Colloid Chemistry, Max Planck Institute of Colloids and Interfaces, 14476 Potsdam, Germany*

*^b^ Functional Materials and Devices, Fraunhofer Institute for Applied Polymer Research IAP, 14476 Potsdam, Germany*

*^c^ Department of Biomaterials, Max Planck Institute of Colloids and Interfaces, 14476 Potsdam, Germany*

*^d^ Department of Radiation Science and Technology, Delft University of Technology, 2629 JB Delft, Netherlands*

**E-Mail:* [*evgeny.senokos@mpikg.mpg.de*](mailto:evgeny.senokos@mpikg.mpg.de)

**Table S1.** Yield and elemental composition of sulfur-carbons thermally condensed between 300 and 900 °C from elemental analysis (EA) showing a decreasing sulfur and increasing carbon content with rising condensation temperature while the oxygen content remains below 2.5 wt.%.

| Condensation temperature (°C) | Yield (%) | C (wt.%) | S (wt.%) | O (wt.%) |
| --- | --- | --- | --- | --- |
| 300 | 36.2 (± 3.2) | 3.3 (± 0.1) | 96.5 (± 0.5) | 0.2 (± 0.1) |
| 400 | 16.7 (± 6.0) | 11.6 (± 3.0) | 88.0 (± 2.9) | 0.4 (± 0.1) |
| 450 | 3.3 (± 0.2) | 41.4 (± 0.2) | 58.5 (± 0.6) | 1.1 (± 0.1) |
| 600 | 2.8 (± 0.3) | 53.3 (± 0.2) | 44.6 (± 0.3) | 2.1 (± 0.1) |
| 700 | 2.1 (± 0.4) | 65.2 (± 0.3) | 33.6 (± 0.3) | 1.2 (± 0.1) |
| 800 | 1.6 (± 0.5) | 75.9 (± 0.3) | 23.4 (± 0.2) | 0.7 (± 0.1) |
| 900 | 1.3 (± 0.2) | 85.8 (± 0.1) | 13.8 (± 0.1) | 0.4 (± 0.1) |

**Table S2.** Specific surface areas (SSA) and pore volumes (V) of sulfur-carbons thermally condensed between 300 and 900 °C obtained from N_2_, CO_2_ and Ar gas physisorption measurements showing a major increase during the lower temperatures till 600 °C and a steady increase afterwards till 900 °C.

| Condensation temperature (°C) | N_2_ physisorption | | CO_2_ physisorption | | Ar physisorption | |
| --- | --- | --- | --- | --- | --- | --- |
|  | SSA_tot_ (m^2^ g^-1^) | V_tot_ (cm^3^ g^-1^) | SSA_micro_ (m^2^ g^-1^) | V_micro_ (cm^3^ g^-1^) | SSA_tot_ (m^2^ g^-1^) | V_tot_ (cm^3^ g^-1^) |
| 300 | 2 | 0.01 | 3 | <0.01 | <1 | 0.00 |
| 400 | 60 | 0.11 | 199 | 0.06 | 8 | 0.02 |
| 450 | 242 | 0.19 | 438 | 0.13 | 320 | 0.18 |
| 600 | 435 | 0.27 | 510 | 0.15 | 568 | 0.24 |
| 700 | 542 | 0.29 | 641 | 0.19 | 708 | 0.27 |
| 800 | 629 | 0.38 | 725 | 0.22 | 965 | 0.40 |
| 900 | 674 | 0.37 | 792 | 0.25 | 1128 | 0.44 |

**Table S3.** Lamellar d-spacing, amphiphilicity factor and average micropore radius calculated from Teubner-Strey^[1]^ and Debye-Beuche^[2]^ models of small- and wide angle X-ray scattering (SAXS/WAXS) measurements (Figure S9) for sulfur-carbons thermally condensed between 300 and 900 °C.^[3]^ The average micropore radius increases from 600 to 700 °C, and the lamellar d-spacing expands rising condensation temperature, while the amphiphilicity factor remains quasi-stable.

| Condensation temperature (°C) | Lamellar d-spacing  (nm) | Amphiphilicity factor f_a_ | Avg. micropore radius  (nm) |
| --- | --- | --- | --- |
| 300 | 0.35 (± 0.01) | -0.95 (± 0.01) | – |
| 400 | 0.35 (± 0.01) | -0.95 (± 0.01) | – |
| 450 | 0.35 (± 0.01) | -0.92 (± 0.01) | 0.40 (± 0.01) |
| 600 | 0.36 (± 0.01) | -0.90 (± 0.01) | 0.47 (± 0.01) |
| 700 | 0.36 (± 0.01) | -0.89 (± 0.01) | 0.74 (± 0.01) |
| 800 | 0.37 (± 0.01) | -0.88 (± 0.01) | 0.96 (± 0.01) |
| 900 | 0.37 (± 0.01) | -0.88 (± 0.01) | 1.16 (± 0.01) |

**Table S4.** Quantitative results of X-ray photoelectron spectroscopy (XPS) measurements of sulfur-carbons thermally condensed between 300 and 900 °C (Figure 3e, f, and S11) revealing a decrease of carbon and increase of sulfur content with rising condensation temperature, while the oxygen content shows no clear correlation with condensation temperature. The C 1s signal is deconvoluted into C (sp^2^) and C (sp^3^) contributions, with their ratios shifting as the C (sp^2^) content increases with increasing condensation temperature.

| Condensation temperature (°C) | Elemental composition (at.%) | | | Carbon distribution | | Peak area (count) | | Ratio (%) | |
| --- | --- | --- | --- | --- | --- | --- | --- | --- | --- |
|  | C | S | O | C (sp^2^) | C (sp^3^) | C (sp^2^) | C (sp^3^) | C (sp^2^) | C (sp^3^) |
| 300 | 49.23 | 48.75 | 2.02 | 0.87 | 27.81 | 276 | 8793 | 3.04 | 96.96 |
| 400 | 57.29 | 37.54 | 5.17 | 5.16 | 31.37 | 2720 | 15624 | 14.83 | 85.17 |
| 450 | 62.03 | 37.44 | 0.53 | 8.33 | 42.37 | 4096 | 21612 | 15.93 | 84.07 |
| 600 | 74.23 | 24.42 | 1.36 | 10.76 | 51.24 | 6531 | 31091 | 17.36 | 82.64 |
| 700 | 81.95 | 14.28 | 3.77 | 21.87 | 42.91 | 23351 | 41182 | 36.18 | 63.82 |
| 800 | 87.61 | 9.59 | 2.81 | 57.46 | 16.35 | 37652 | 10711 | 77.85 | 22.15 |
| 900 | 91.76 | 5.56 | 2.68 | 78.99 | 0 | 52935 | 0.2 | 100 | 0 |

**Table S5.** Semi-quantitative EDX analysis of the sulfur-carbon produced at 600 °C in non-cycled pristine electrode and after 500 GCD cycles.

| Element | Pristine | Post-cycling |
| --- | --- | --- |
| C (wt.%) | 62.9 (±1.3) | 15.3 (±0.4) |
| S (wt.%) | 28.9 (±1.6) | 7.1 (±3.4) |
| C/S | 2.2 (±0.1) | 2.2 (±1.0) |
| O (wt.%) | 8.2 (±0.3) | 20.9 (±1.5) |
| Na (wt.%) | – | 37.9 (±2.4) |
| F (wt.%) | – | 16.7 (±2.3) |

**Table S6.** Cut-off and specific capacity values used for the calculation of the sulfur utilization and energy density during discharge after full activation for sulfur-carbons thermally condensed between 300 and 900 °C. The cut-off capacities increase with rising condensation temperature, while the total capacity peaks at 400 °C before declining. Similarly, the energy density reaches its maximum at 450 °C and decreasing thereafter.

| Condensation temperature (°C) | Cut-off capacity (mAh g^-1^) | Specific capacity (mAh g^-1^) | Sulfur utilization (%) | Energy density  (Wh $kg_{\mathrm{SC}}^{-1}$) |
| --- | --- | --- | --- | --- |
| 300 | 93 | 674 | 35 | 774 |
| 400 | 73 | 1053 | 66 | 1260 |
| 450 | 127 | 997 | 89 | 1200 |
| 600 | 130 | 784 | 88 | 933 |
| 700 | 129 | 694 | 99 | 781 |
| 800 | 153 | 510 | 91 | 544 |
| 900 | 167 | 404 | 99 | 349 |

**Table S7.** Specific capacity and energy density of the sulfur-carbons normalized by the total mass of the electrode.

| Condensation temperature (°C) | Specific capacity  (mAh g^-1^) | Energy density  (Wh kg^-1^) |
| --- | --- | --- |
| 300 | 472 | 542 |
| 400 | 737 | 882 |
| 450 | 698 | 840 |
| 600 | 549 | 653 |
| 700 | 486 | 547 |
| 800 | 357 | 381 |
| 900 | 283 | 244 |

**Table S8.** Overview of density functional theory (DFT) -derived computational models and their purposes, including their structural characteristics, intended physical purpose, chain lengths, and degrees of confinement.

| Model name | Configuration | Purpose | Chain | Confinement |
| --- | --- | --- | --- | --- |
| Model 1 | Fig. S23 | Reveal S-C interaction | - | - |
| M1-33%S | Fig. S24 | Reveal Na-S-C interaction in a non-S-saturated C system | - | - |
| M1-100%S | Fig. S24 | Reveal Na-S-C interaction in an S-saturated C system | - | - |
| M1-FreeChain | Fig. S24 | Reveal Na-S-C interaction in an S-saturated C system with the presence of a free chain | 4 | A chain enclosed by 2 graphene sheets |
| iso-4S-chain | Fig. S23 | Medium chain interaction with Na, zero confinement | 4 | None, isolated sheet |
| inter-8S-chain | Fig. S23 | Long chain interaction with Na, medium confinement | 8 | Medium, Interaction from 2 sides |
| 2-inter-8S-chain | Fig. S23 | Long chain interaction with Na, high confinement | 8 | High, Interaction from 4 sides |
| 2-inter-2S-chain | Fig. S23 | Short Chain interaction with Na, high confinement | 2 | Very High, Interaction from 4 sides, Closer distances enabled by the small chain |

**Table S9.** Bonding energies for S and Na at carbon edges and vacancies were calculated as: ${E_{bond}=E}_{host+atom}-(E_{host}+E_{atom})$, where $E_{atom}$ is the total energy of an isolated atom placed in a vacuum supercell. With this definition, negative values indicate that the combined system is more stable than the separated components, and therefore correspond to favorable bonding. More negative energies reflect stronger interactions. The calculations are performed in Model 1.

| Type of bonding | Energy (eV) |
| --- | --- |
| S at carbon edge | –5.971 |
| S at carbon vacancy | –4.954 |
| Na at carbon edges | –2.801 |
| Na at carbon vacancy | –1.638 |

**Table S10.** Anchoring strengths for sulfur chains at the sulfated edges were computed analogously: ${E_{anchor}=E}_{host+chain}-(E_{host}+E_{chain})$, $E_{chain}$ is the energy of an isolated chain $S_{n}$ of the same length in a vacuum supercell. Negative anchoring strengths indicate thermodynamically favorable attachments to sulfated edges. The calculations are performed in Model 1.

| Chain length | Anchoring strength (eV) |
| --- | --- |
| 1 | –0.629 |
| 2 | –0.092 |
| 3 | –0.141 |
| 4 | –0.144 |
| 5 | –0.222 |

**Table S11.** Energetics of Na-induced sulfur extraction from carbon edges. Energies are reported relative to the convex hull of the most stable C–S bonding configurations. Positive values indicate that Na “stealing” S from the carbon edge to form Na–S is energetically unfavorable.

| Model name | Energetic C-S edge bonding preference in competition with the formation of free Na-S (eV) |
| --- | --- |
| M1-33%S at Na_0.01_S_0.03_C | 2.86 |
| M1-33%S at Na_0.03_S_0.03_C | 2.52 |
| M1-100%S at Na_0.02_S_0.06_C | 2.18 |

**Table S12.** Comparison of sulfur utilization and capacities based on total amount of active material (AM) of the sulfur-carbons condensed between 300 and 900 °C with reported cathode materials.

| Material | S content  (wt.%) | Sulfur loading  (mg_s_ cm^-2^) | C-Rate | Capacity  (mAh $g_{\mathrm{AM}}^{-1}$) | S utilization  (%) | Reference |
| --- | --- | --- | --- | --- | --- | --- |
| 300 | 96.5 | 0.65 | 0.1 | 674 | 35 | This work |
| 400 | 88.2 | 0.63 |  | 1053 | 66 |  |
|  |  | 1.77 |  | 954 |  |  |
| 450 | 58.3 | 0.24 |  | 997 | 89 |  |
| 600 | 44.6 | 0.23 |  | 784 | 88 |  |
| 700 | 33.6 | 0.17 |  | 694 | 99 |  |
| 800 | 23.4 | 0.17 |  | 510 | 91 |  |
| 900 | 14.0 | 0.08 |  | 404 | 99 |  |
| Porous Carbon | 47.0 | 0.79 | 0.1 | 409 | 52 | ^[4]^ |
| TeS@pPAN | 42.1 | 0.51 | ~0.14 | 518 | 73 | ^[5]^ |
| MPCFs | 61.1 | 0.35 | 0.1 | 693 | 67 | ^[6]^ |
| S@Co-HC | 47.0 | – | ~0.13 | 324 | 41 | ^[7]^ |
| S@Fe-HC | 40.0 | 0.44 | ~0.15 | 213 | 32 | ^[8]^ |
| ACC-40S | 40.0 | 1.00 | 0.1 | 492 | 73 | ^[9]^ |
| S/Ni-MOF-2D | 48.6 | 0.49 | 0.2 | 201 | 25 | ^[10]^ |
| NiS_2_@NPCTs/S | 56.0 | – | ~0.1 | 650 | 69 | ^[11]^ |
| CS ZCS@S | 57.0 | 0.69 | ~0.28 | 434 | 43 | ^[12]^ |
| S/ELSC-40 | 40.0 | 1.00 | 0.1 | 528 | 79 | ^[13]^ |
| CS90-rGO_(S)_ | 88.7 | 2.14 | ~0.14 | 496 | 33 | ^[14]^ |
| S/PCs | 52.3 | – | 0.1 | 725 | 83 | ^[15]^ |
| RGO/SiO_2_/S | 33.0 | 0.79 | 0.1 | 219 | 40 | ^[16]^ |
| S@*i*MCHS | 46.0 | 1.32 | ~0.13 | 413 | 54 | ^[17]^ |
| CN/Au/S | 56.5 | – | ~0.11 | 463 | 49 | ^[18]^ |
| S/TiN-TiO_2_@MCCFs | 56.9 | 1.08 | ~0.11 | 397 | 42 | ^[19]^ |
| Material | S loading  (wt.%) | Sulfur loading  (mg_s_ cm^-2^) | C-Rate | Capacity  (mAh $g_{\mathrm{AM}}^{-1}$) | S utilization  (%) | Reference |
| S@Ni-NFCs | 36.0 | 0.25 | 0.2 | 207 | 34 | ^[20]^ |
| MPC-900-S | 34.0 | 0.70 | 0.2 | 424 | 74 | ^[21]^ |
| Co-NP | 38.0 | 1.00 | 0.1 | 316 | 50 | ^[22]^ |
| Covalent-SC | 36.9 | 0.56 | 0.16 | 455 | 74 | ^[23]^ |
| SeS@pPAN | 36.9 | 0.59 | ~0.16 | 382 | 62 | ^[24]^ |
| N,S-HPC/S | 22.0 | 0.17 | ~0.31 | 96 | 26 | ^[25]^ |
| NGNS/S | 25.0 | – | 0.1 | 30 | 7 | ^[26]^ |
| ZIF-8/S | 50.0 | 0.32 | 0.1 | 508 | 61 | ^[27]^ |
| Ti_3_C_2_T_x_@S | 55.0 | – | ~0.02 | 225 | 24 | ^[28]^ |
| YP50F/S | 35.8 | 1.50 | 0.1 | 408 | 68 | ^[29]^ |
| FSPAN | 56.3 | 0.90 | 0.1 | 736 | 78 | ^[30]^ |
| FTe_0.01_S_0.99_PAN | 49.7 | 0.80 | 0.1 | 680 | 82 | ^[30]^ |
| Ca-O_4_N-C@S | 55.2 | 0.55 | 0.1 | 726 | 79 | ^[31]^ |
| S@MHCS-3 | 39.5 | 0.89 | 0.1 | 325 | 49 | ^[32]^ |
| S@Co-NMCN | 36.0 | 2.00 | 0.1 | 330 | 55 | ^[33]^ |

The XPS peak-fitting parameters, which were applied to the C 1s high-resolution spectra (Figure 3e and S11b) are summarized in Table S13. To accurately resolve the evolution of thiol-derived carbon species during the high-temperature treatments, the C=C sp², C–C sp³, and associated oxygen-bound species were fitted using consistent and physically justified constraints across all sulfur-carbon hybrids. The binding energies of C–C bonds, mainly attributed to sp^3^-hybridized carbons, were charge-corrected by setting them equal to 285.0 eV for all temperatures from 300 °C to 900 °C, and their oxygen-containing components
(C–O, C=O, and O–C=O) were equally fitted at 286.5, 287.8, and 289.0 eV, respectively.^[34-36]^ Meanwhile, the C=C peak, mainly attributed to graphitic sp²-hybridized carbons and the π–π* shake-up satellite were consistently fitted at 284.5 eV and approximately 291.0 eV, respectively.^[34, 37]^

To ensure internal consistency in the data processing, identical linewidth constraints were applied to chemically related components. In particular, the asymmetric line shape LA(1.2,2.5,5), characteristic of graphitic sp^2^-hybridized carbons, was applied to the C=C peak with full width at half maximum (FWHM) constrained to 0.4–0.8 eV, which is typical for ordered graphitic environments. The aliphatic C–C/C–H peak and its oxygen-functionalized derivatives were fitted using a FWHM range of 0.9–1.3 eV with the symmetric line shape GL(30), which is widely accepted for amorphous carbon species and polymeric or partially oxidized carbon networks in XPS data processing. These constraints were enforced uniformly across all temperatures to avoid artificial broadening or misallocation of peak areas, ensuring that the observed intensity variations reflect the underlying chemical evolution rather than fitting artifacts. As a result of the consistent application of these physically grounded fitting parameters, all component peaks exhibit uniformly constrained linewidths. This consistency across temperatures confirms that the peak widths were not artificially broadened or over-constrained, and it further supports that the peak-fitting is self-consistent and reflects the underlying chemical evolution rather than fitting artifacts.

The peak-fitting parameters, which were applied to the S 2p high-resolution spectra (Figure 3f, S11c, and S15) are summarized in Table S14. Each spectral component was fitted as a spin-orbit doublet (2p_3/2_ and 2p_1/2_) with an area ratio of 2:1 and a fixed energy separation of 1.20 eV, which reflects the intrinsic spin-orbit degeneracy of sulfur. These constraints were uniformly applied across all temperatures to ensure physically meaningful and internally consistent deconvolution of the polysulfide and sulfur allotrope species.

To clearly resolve the peak-fitting of terminal and central sulfur environments within the polysulfide chains, the FWHM was constrained primarily within a physically justified range of 1.0–1.3 eV for all sulfur-carbon hybrids up to 600 °C. The only exception occurs at 300 °C for the central sulfur component, where a slightly narrower FWHM range of 0.8–1.3 eV was allowed. This reflects the contribution of low-sulfur-content organic sulfide or thiol-like species (R–SH, R–S–R′).^[38-41]^ At reaction temperatures between 700 and 900 °C, extensive scrambling reactions between polysulfides and sulfur allotropes promote the conversion toward shorter-chain, lower-sulfur-content sulfur-carbon hybrids. These species can exhibit narrower intrinsic peak widths. Therefore, a consistent FWHM constraint of 0.8–1.3 eV was uniformly applied across these sulfur environments above 700 °C. This approach prevents artificial broadening and maintains the chemical validity of the extracted peak components, particularly under high-temperature conditions where the distribution of sulfur bonding motifs becomes more simplified.

**Table S13.** XPS fitting parameters of the C 1s high-resolution spectra

|  | Peak Assignment | Peak Identifier | BE (eV) | Peak (eV) Constraints | FWHM (eV) | FWHM (eV)  Constraints | Area (%) | Lineshape |
| --- | --- | --- | --- | --- | --- | --- | --- | --- |
| 300 °C | C=C sp^2^ | A | 284.5 | 284.5 | 0.80 | 0.4–0.8 | 1.77 | LA(1.2,2.5,5) |
|  | C–C sp^3^ | B | 285.0 | A + 0.5 | 1.15 | 0.9–1.3 | 56.49 | GL(30) |
|  | C–OH, C–O–C | C | 286.5 | A + 2.0 | 1.15 | B*1 | 11.71 | GL(30) |
|  | C=O | D | 287.8 | A + 3.3 | 1.15 | B*1 | 5.51 | GL(30) |
|  | O–C=O | E | 289.0 | A + 4.5 | 1.15 | B*1 | 1.28 | GL(30) |
|  | π-π* sat | F | 290.7 | A + 6.2 | 2.70 | 2.7-2.75 | 7.22 | GL(30) |
|  | Thiol | G | 285.7 | - | 1.15 | 0.9–1.3 | 16.02 | GL(30) |
| 400 °C | C=C sp^2^ | A | 284.5 | 284.5 | 0.80 | 0.4–0.8 | 9.01 | LA(1.2,2.5,5) |
|  | C–C sp^3^ | B | 285.0 | A + 0.5 | 1.15 | 0.9–1.3 | 51.75 | GL(30) |
|  | C–OH, C–O–C | C | 286.5 | A + 2.0 | 1.15 | B*1 | 10.84 | GL(30) |
|  | C=O | D | 287.8 | A + 3.3 | 1.15 | B*1 | 4.71 | GL(30) |
|  | O–C=O | E | 289.0 | A + 4.5 | 1.15 | B*1 | 1.32 | GL(30) |
|  | π-π* sat | F | 290.7 | A + 6.2 | 2.70 | 2.7-2.75 | 2.70 | GL(30) |
|  | Thiol | G | 285.7 | - | 1.15 | 0.9–1.3 | 19.66 | GL(30) |
| 450 °C | C=C sp^2^ | A | 284.5 | 284.5 | 0.80 | 0.4–0.8 | 12.95 | LA(1.2,2.5,5) |
|  | C–C sp^3^ | B | 285.0 | A + 0.5 | 1.03 | 0.9–1.3 | 68.30 | GL(30) |
|  | C–OH, C–O–C | C | 286.5 | A + 2.0 | 1.03 | B*1 | 4.53 | GL(30) |
|  | C=O | D | 287.8 | A + 3.3 | 1.03 | B*1 | 2.87 | GL(30) |
|  | O–C=O | E | 289.0 | A + 4.5 | 1.03 | B*1 | 1.57 | GL(30) |
|  | π-π* sat | F | 290.7 | A + 6.2 | 2.71 | 2.7-2.75 | 1.96 | GL(30) |
|  | Thiol | G | 285.7 | - | 1.04 | 0.9–1.3 | 7.82 | GL(30) |
| 600 °C | C=C sp^2^ | A | 284.5 | 284.5 | 0.80 | 0.4–0.8 | 13.21 | LA(1.2,2.5,5) |
|  | C–C sp^3^ | B | 285.0 | A + 0.5 | 1.15 | 0.9–1.3 | 69.61 | GL(30) |
|  | C–OH, C–O–C | C | 286.5 | A + 2.0 | 1.15 | B*1 | 3.94 | GL(30) |
|  | C=O | D | 287.8 | A + 3.3 | 1.15 | B*1 | 2.90 | GL(30) |
|  | O–C=O | E | 289.0 | A + 4.5 | 1.15 | B*1 | 2.40 | GL(30) |
|  | π-π* sat | F | 290.7 | A + 6.2 | 2.71 | 2.7-2.75 | 6.00 | GL(30) |
|  | Thiol | G | 285.6 | - | 1.15 | 0.9–1.3 | 1.94 | GL(30) |
| 700 °C | C=C sp^2^ | A | 284.5 | 284.5 | 0.80 | 0.4–0.8 | 29.69 | LA(1.2,2.5,5) |
|  | C–C sp^3^ | B | 285.0 | A + 0.5 | 1.04 | 0.9–1.3 | 52.36 | GL(30) |
|  | C–OH, C–O–C | C | 286.5 | A + 2.0 | 1.04 | B*1 | 6.54 | GL(30) |
|  | C=O | D | 287.8 | A + 3.3 | 1.04 | B*1 | 3.09 | GL(30) |
|  | O–C=O | E | 289.0 | A + 4.5 | 1.04 | B*1 | 1.82 | GL(30) |
|  | π-π* sat | F | 290.7 | A + 6.2 | 2.71 | 2.7-2.75 | 3.21 | GL(30) |
|  | Thiol | G | 285.9 | - | 1.03 | 0.9–1.3 | 3.27 | GL(30) |
| 800 °C | C=C sp^2^ | A | 284.5 | 284.5 | 0.73 | 0.4–0.8 | 65.59 | LA(1.2,2.5,5) |
|  | C–C sp^3^ | B | 285.0 | A + 0.5 | 1.02 | 0.9–1.3 | 18.66 | GL(30) |
|  | C–OH, C–O–C | C | 286.5 | A + 2.0 | 1.02 | B*1 | 5.63 | GL(30) |
|  | C=O | D | 287.8 | A + 3.3 | 1.02 | B*1 | 2.70 | GL(30) |
|  | O–C=O | E | 289.0 | A + 4.5 | 1.02 | B*1 | 1.75 | GL(30) |
|  | π-π* sat | F | 290.7 | A + 6.2 | 2.70 | 2.7-2.75 | 3.78 | GL(30) |
|  | Thiol | G | 286.0 | - | 1.02 | 0.9–1.3 | 1.90 | GL(30) |
| 900 °C | C=C sp^2^ | A | 284.5 | 284.5 | 0.80 | 0.4–0.8 | 86.07 | LA(1.2,2.5,5) |
|  | C–C sp^3^ | B | 285.0 | A + 0.5 | 1.12 | 0.9–1.3 | 0 | GL(30) |
|  | C–OH, C–O–C | C | 286.5 | A + 2.0 | 1.12 | B*1 | 4.82 | GL(30) |
|  | C=O | D | 287.8 | A + 3.3 | 1.12 | B*1 | 2.05 | GL(30) |
|  | O–C=O | E | 289.0 | A + 4.5 | 1.12 | B*1 | 1.40 | GL(30) |
|  | π-π* sat | F | 290.7 | A + 6.2 | 2.75 | 2.7-2.75 | 4.91 | GL(30) |
|  | Thiol | G | 286.0 | - | 1.12 | 0.9–1.3 | 0.75 | GL(30) |

**Table S14.** XPS fitting parameters of the S 2p high-resolution spectra

|  | Peak Assignment | Peak  Identifier | BE (eV) | Peak (eV) Constraints | FWHM (eV) | FWHM (eV) Constraints | Area (%) | Lineshape |
| --- | --- | --- | --- | --- | --- | --- | --- | --- |
| 300 °C | Terminal S Polysulfide (2p_3/2_) | A | 161.6 | - | 1.27 | 1.0–1.3 | 2.91 | GL(30) |
|  | Terminal S Polysulfide (2p_1/2_) | B | 162.8 | A+1.2 | 1.27 | A*1 | 1.45 |  |
|  | Central S polysulfide (2p_3/2_) | C | 163.6 | - | 0.80 | 0.8–1.3 | 22.03 |  |
|  | Central S polysulfide (2p_1/2_) | D | 164.8 | C+1.2 | 0.80 | C*1 | 10.99 |  |
|  | Sulfur S_8_ (2p_3/2_) | E | 164.1 | - | 1.30 | 1.0–1.3 | 32.26 |  |
|  | Sulfur S_8_ (2p_1/2_) | F | 165.3 | E+1.2 | 1.30 | E*1 | 16.10 |  |
|  | Sulfinly (2p_3/2_) | G | 165.8 | - | 1.30 | 1.0–1.3 | 5.91 |  |
|  | Sulfinly (2p_1/2_) | H | 167.0 | G+1.2 | 1.30 | F*1 | 2.95 |  |
|  | Surfite (2p_3/2_) | I | 168.6 | - | 1.30 | 1.0–1.3 | 3.60 |  |
|  | Surfite (2p_1/2_) | J | 169.8 | I+1.2 | 1.30 | I*1 | 1.80 |  |
| 400 °C | Terminal S Polysulfide (2p_3/2_) | A | 161.6 | - | 1.20 | 1.0–1.3 | 3.74 | GL(30) |
|  | Terminal S Polysulfide (2p_1/2_) | B | 162.8 | B+1.2 | 1.20 | A*1 | 1.87 |  |
|  | Central S polysulfide (2p_3/2_) | C | 163.8 | - | 1.28 | 1.0–1.3 | 56.23 |  |
|  | Central S polysulfide (2p_1/2_) | D | 165.0 | C+1.2 | 1.28 | C*1 | 28.06 |  |
|  | Sulfinly (2p_3/2_) | E | 165.8 | - | 1.28 | 1.0–1.3 | 6.74 |  |
|  | Sulfinly (2p_1/2_) | F | 167.0 | E+1.2 | 1.28 | E*1 | 3.36 |  |
| 450 °C | Terminal S Polysulfide (2p_3/2_) | A | 161.5 | - | 1.14 | 1.0–1.3 | 5.45 | GL(30) |
|  | Terminal S Polysulfide (2p_1/2_) | B | 162.7 | B+1.2 | 1.14 | A*1 | 2.72 |  |
|  | Central S polysulfide (2p_3/2_) | C | 163.9 | - | 1.15 | 1.0–1.3 | 56.52 |  |
|  | Central S polysulfide(2p_1/2_) | D | 165.1 | C+1.2 | 1.15 | C*1 | 28.21 |  |
|  | Sulfinly (2p_3/2_) | E | 165.7 | - | 1.00 | 1.0–1.3 | 3.10 |  |
|  | Sulfinly (2p_1/2_) | F | 166.9 | E+1.2 | 1.00 | E*1 | 1.55 |  |
|  | Sulfonyl (2p_3/2_) | G | 167.9 | - | 1.25 | 1.0–1.3 | 1.64 |  |
|  | Sulfonyl (2p_1/2_) | H | 169.1 | G+1.2 | 1.25 | G*1 | 0.82 |  |
| 600 °C | Terminal S Polysulfide (2p_3/2_) | A | 161.7 | - | 1.30 | 1.0–1.3 | 3.19 | GL(30) |
|  | Terminal S Polysulfide (2p_1/2_) | B | 162.9 | B+1.2 | 1.30 | A*1 | 1.59 |  |
|  | Central S polysulfide (2p_3/2_) | C | 164.0 | - | 1.12 | 1.0–1.3 | 56.43 |  |
|  | Central S polysulfide(2p_1/2_) | D | 165.2 | C+1.2 | 1.12 | C*1 | 28.16 |  |
|  | Sulfinly (2p_3/2_) | E | 165.7 | - | 1.30 | 1.0–1.3 | 4.59 |  |
|  | Sulfinly (2p_1/2_) | F | 166.9 | E+1.2 | 1.30 | E*1 | 2.29 |  |
|  | Sulfonyl (2p_3/2_) | G | 168.1 | - | 1.30 | 1.0–1.3 | 2.50 |  |
|  | Sulfonyl (2p_1/2_) | H | 169.3 | G+1.2 | 1.30 | G*1 | 1.25 |  |
| 700 °C | Terminal S Polysulfide (2p_3/2_) | A | 161.7 | - | 1.30 | 1.0–1.3 | 3.13 | GL(30) |
|  | Terminal S Polysulfide (2p_1/2_) | B | 162.9 | B+1.2 | 1.30 | A*1 | 1.56 |  |
|  | Central S polysulfide (2p_3/2_) | C | 164.0 | - | 0.89 | 0.8–1.3 | 56.83 |  |
|  | Central S polysulfide(2p_1/2_) | D | 165.2 | C+1.2 | 0.89 | C*1 | 28.36 |  |
|  | Sulfinly (2p_3/2_) | E | 165.4 | - | 0.96 | 0.8–1.3 | 4.74 |  |
|  | Sulfinly (2p_1/2_) | F | 166.6 | E+1.2 | 0.96 | E*1 | 2.36 |  |
|  | Sulfonyl (2p_3/2_) | G | 167.7 | - | 0.97 | 0.8–1.3 | 2.02 |  |
|  | Sulfonyl (2p_1/2_) | H | 168.9 | G+1.2 | 0.97 | G*1 | 1.01 |  |
| 800 °C | Terminal S Polysulfide (2p_3/2_) | A | 161.9 | - | 1.30 | 1.0–1.3 | 2.43 | GL(30) |
|  | Terminal S Polysulfide (2p_1/2_) | B | 163.1 | B+1.2 | 1.30 | A*1 | 1.21 |  |
|  | Central S polysulfide (2p_3/2_) | E | 163.9 | - | 0.84 | 0.8–1.3 | 60.11 |  |
|  | Central S polysulfide(2p_1/2_) | F | 165.1 | C+1.2 | 0.84 | C*1 | 30.00 |  |
|  | Polythiophene (2p_3/2_) | G | 165.2 | - | 0.84 | 0.8–1.3 | 4.16 |  |
|  | Polythiophene (2p_1/2_) | H | 166.3 | E+1.2 | 0.84 | G*1 | 2.08 |  |
| 900 °C | Terminal S Polysulfide (2p_3/2_) | A | 162.0 | - | 1.30 | 1.0–1.3 | 1.83 | GL(30) |
|  | Terminal S Polysulfide (2p_1/2_) | B | 163.2 | B+1.2 | 1.30 | A*1 | 0.91 |  |
|  | Central S polysulfide (2p_3/2_) | E | 164.0 | - | 0.79 | 0.8–1.3 | 59.07 |  |
|  | Central S polysulfide(2p_1/2_) | F | 165.2 | C+1.2 | 0.79 | C*1 | 29.48 |  |
|  | Polythiophene (2p_3/2_) | G | 165.0 | - | 0.93 | 0.8–1.3 | 5.81 |  |
|  | Polythiophene (2p_1/2_) | H | 166.2 | E+1.2 | 0.93 | G*1 | 2.90 |  |


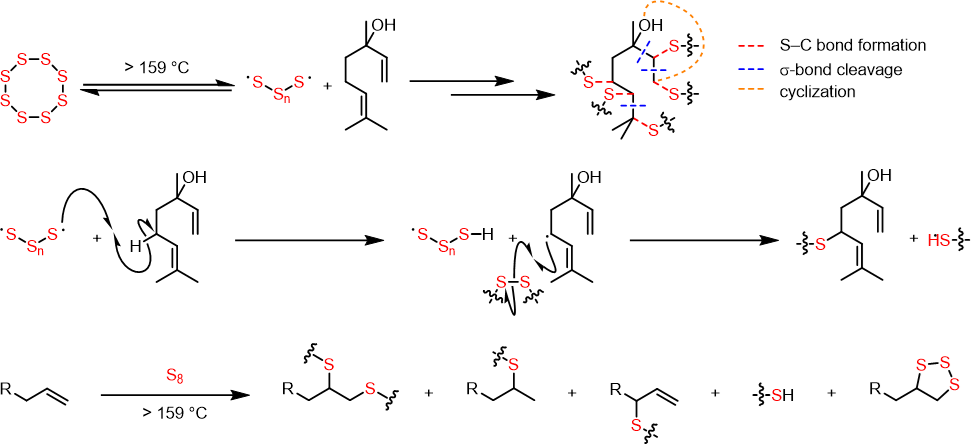


**Figure S1.** Schematic representation of the inverse vulcanization reaction mechanism, highlighting S–C bond formation, potential σ-bond cleavage of C–C bonds, cyclization, allylic hydrogen abstraction, and the resulting products from the reaction of S_8_ with terminal double bonds.^[42-43]^


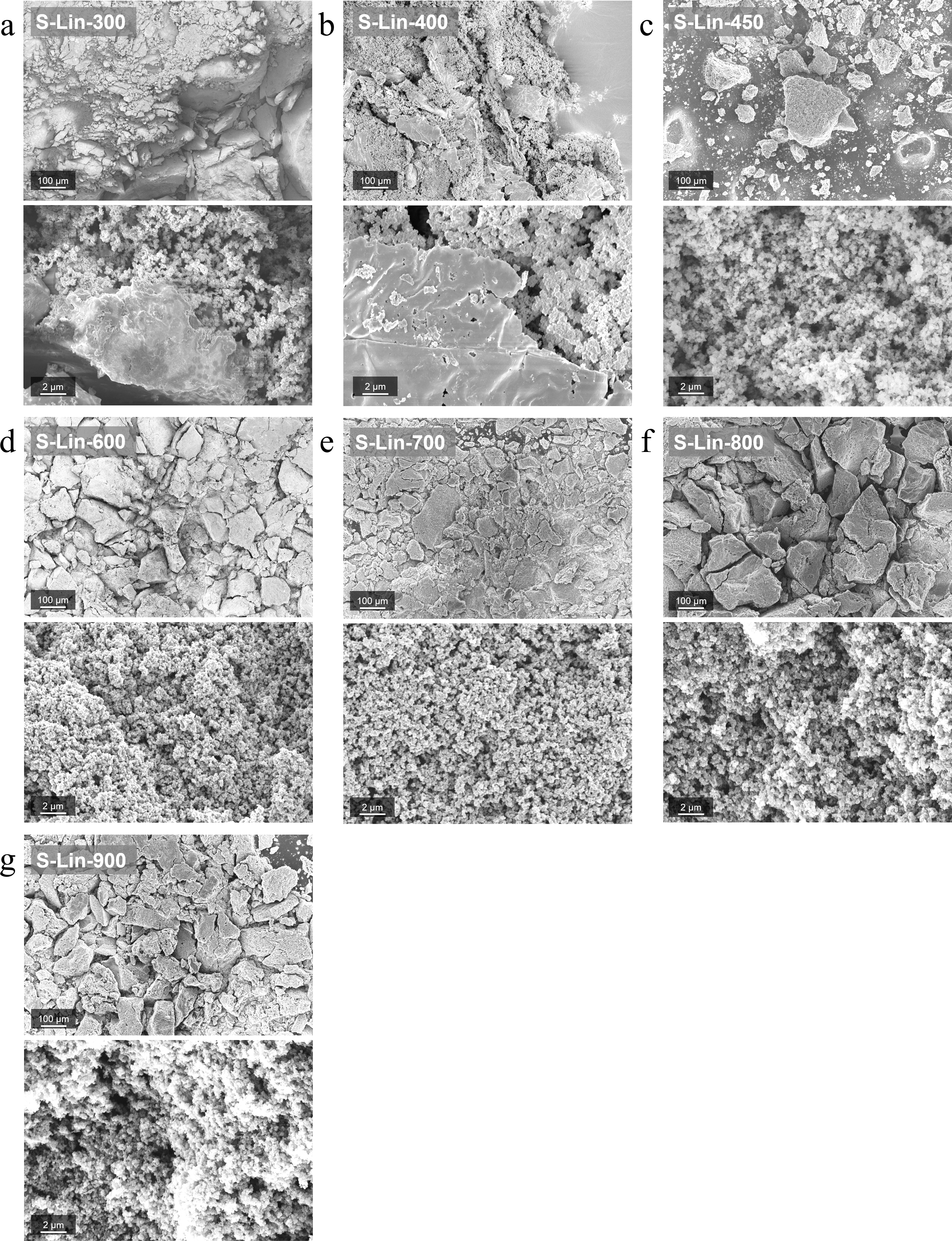


**Figure S2.** a-g) Scanning electron microscopy (SEM) images showing the evolving macroscopic structure of the sulfur-carbons thermally condensed between 300 and 900 °C, displaying chunks made from nanospheres that are covered by a molten polymer-like structure at below 450 °C, and become the dominant structure if the boiling point of sulfur is surpassed.


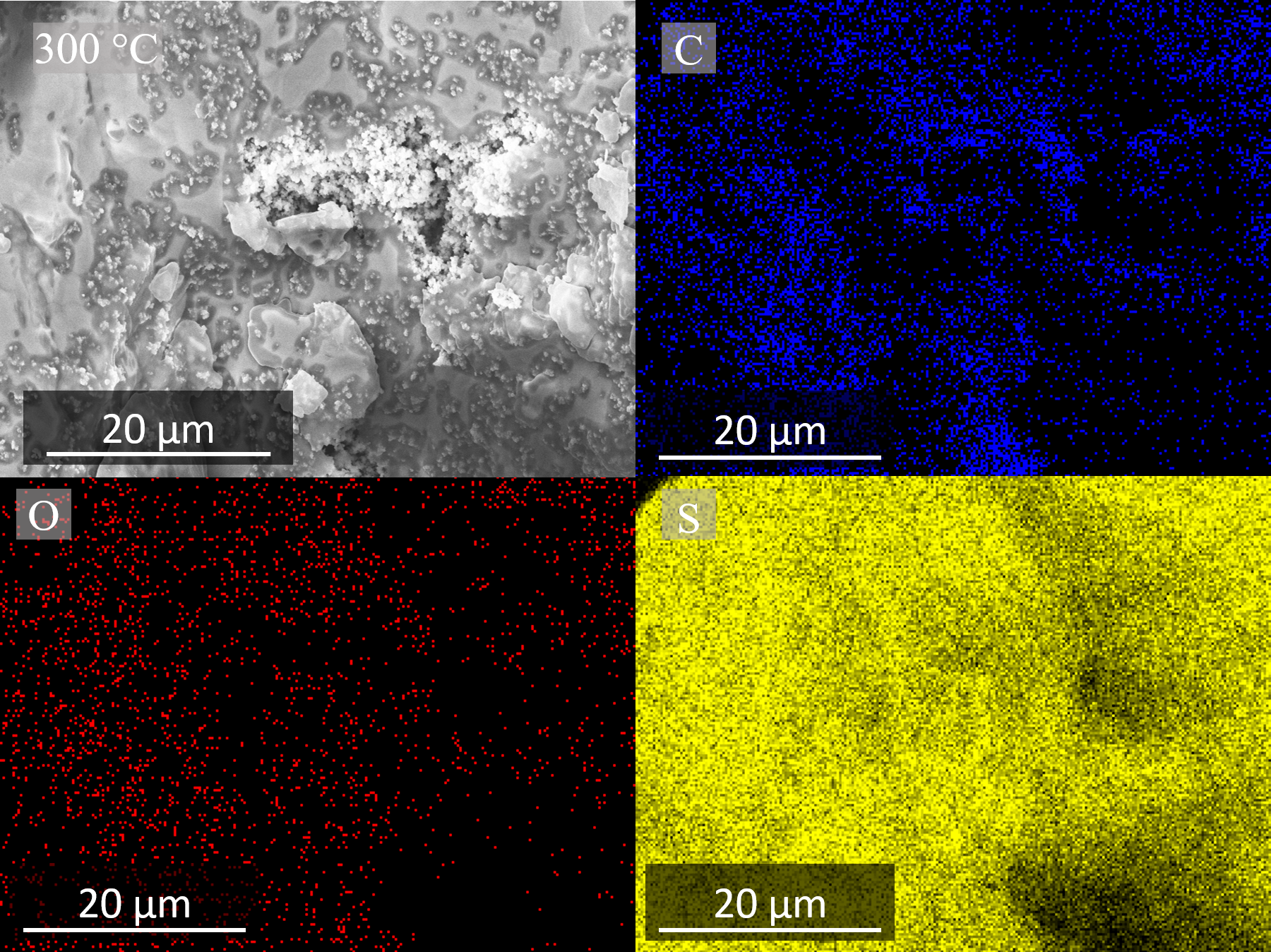


**Figure S3.** Scanning electron microscopy (SEM) image and energy-dispersive X-ray (EDX) mapping of sulfur-carbon thermally condensed at 300 °C, revealing a molten polymer-like structure with embedded submicron spherical-like particles in the SEM image and a homogenous distribution of carbon, oxygen and sulfur in the EDX mapping.


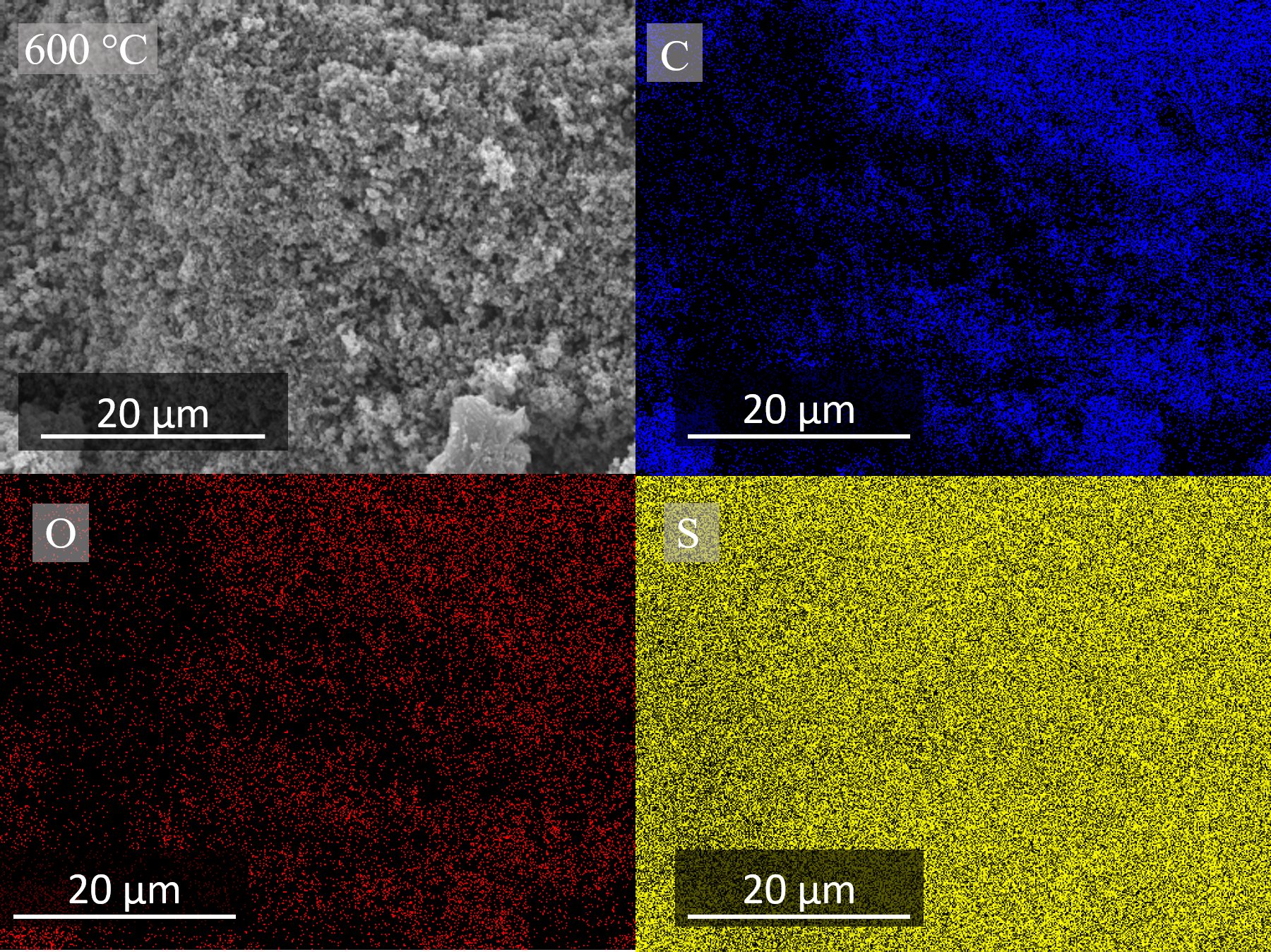


**Figure S4.** SEM image and EDX mapping of sulfur-carbon thermally condensed at 600 °C, revealing the predominant composition of submicron spherical-like particles in the SEM image, with a homogenous distribution of carbon, oxygen and sulfur in the EDX mapping.


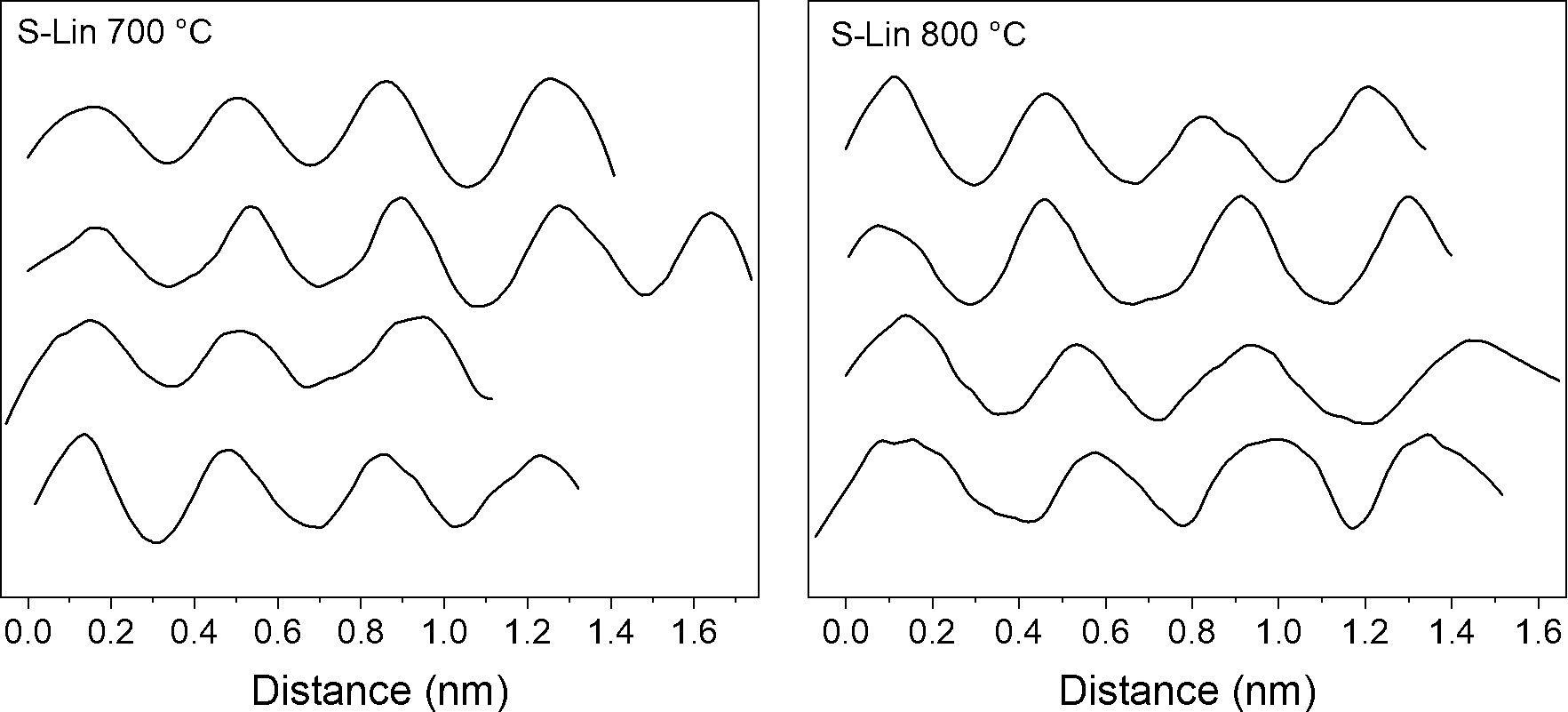


**Figure S5.** Line profiles of HR-TEM images shown in Figure 1d from evolved pseudo-graphitic graphitic stacks, showing the average interlayer distance using the line profile of in the selected region in the inverse Fast Fourier Transformed (FFT) image.

**Figure S6.** High-resolution dark field (upper row) and the corresponding bright field (lower row) scanning transmission electron microscopy (HR-STEM) images of sulfur-carbons thermally condensed at 600, 700, and 800 °C, revealing an amorphous structure at 600 °C and the formation of nanovoids between the pseudo-graphitic carbon layers at 700 and 800 °C.


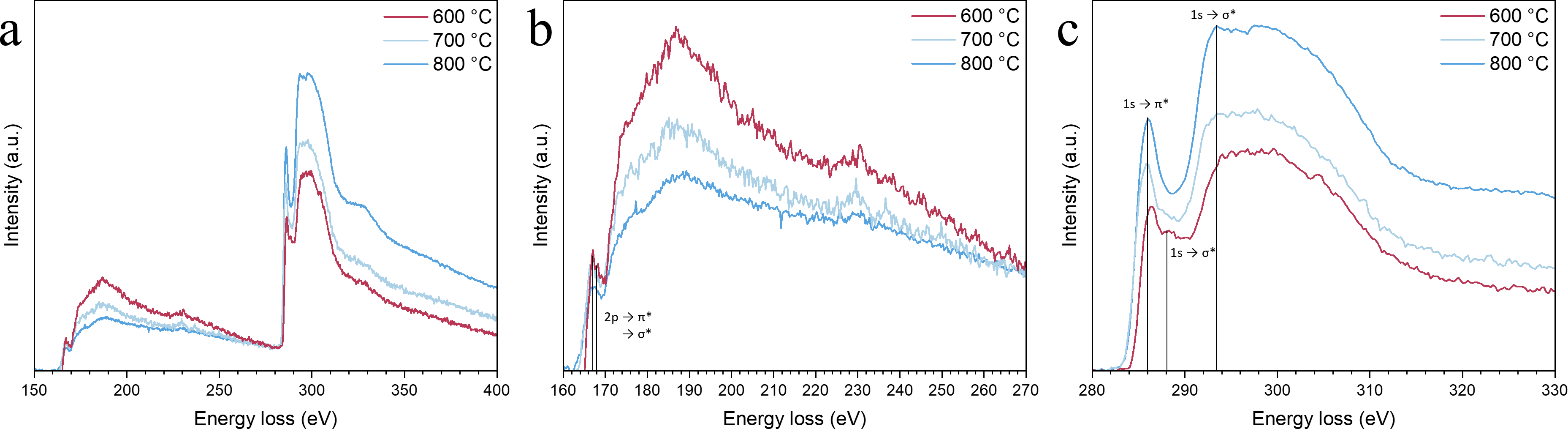


**Figure S7.** a) Electron energy loss spectra (EELS) of sulfur-carbons thermally condensed at 600, 700 and 800 °C revealing the presence of b) sulfur L_2,3_- and c) carbon K-edge, with distinct electron transitions from atomic- to anti-binding molecular orbitals.


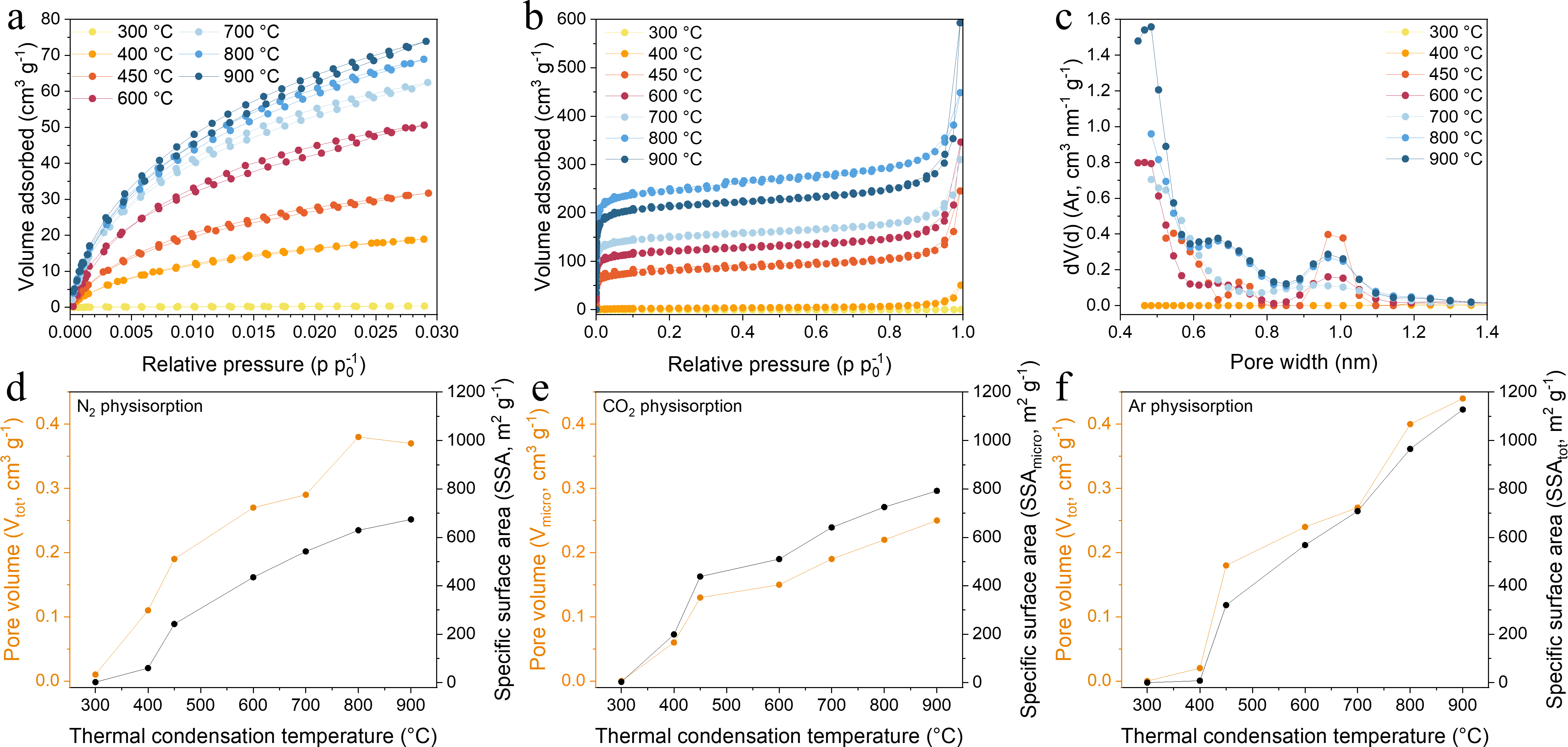


**Figure S8.** a) CO_2_ and b) Ar physisorption isotherms, along with c) pore size distribution (PSD) from Ar physisorption, for sulfur-carbons condensed between 300 and 900 °C showing the progressive development of porosity in the microporous region. Summary of the evolution of the pore volume (V) and specific surface area (SSA) from d) N_2_ (Figure 2a), e) CO_2_ (Figure S8a) and f) Ar physisorption (Figure S8b), illustrating the transition from very low surface area and pore volume at lower condensation temperatures (300-400 °C) to higher values at elevated condensation temperatures (450-900 °C).

CO₂ physisorption is particularly well suited for the analysis of (ultra-)microporous carbonaceous materials. However, sulfur-carbons condensed at low temperatures incorporate up to 96.5 wt.% of sulfur, which may interact with the quadrupole moment of CO_2_, thereby influencing pore filling pressures.^[44]^ This interaction complicates the direct correlation between pore filling pressure and pore size, potentially leading to systematic over- or underestimation.^[45]^ Therefore argon physisorption measurements were carried out to validate the data obtained from CO₂-physisorption (Figures S8b, c and f).

Pore size distribution (PSD) from CO_2_ physisorption reveals a prominent peak at 0.36 nm, likely corresponding to the interlayer spaces in the carbon-matrix, while the broad peaks in the region between 0.40 and 0.70 nm are associated with the removal of different sulfur species from S_2_ to S_8_. The major fraction of the micropore specific surface area and pore volume is developed during thermal condensation at 450 °C, driven by the removal of non-bonded sulfur, preceding the formation of locally ordered carbonaceous network. This suggests a structural reorganization of the amorphous sulfur-carbon phase which starts at lower condensation temperatures. The mesoporous region of the PSD reveals a uniform distribution with a distinct peak at 2.4 nm, emerging at 400 °C and reaching its maximum intensity at 450 °C. This suggests that mesopores in the sulfur-carbons primarily originate from the evaporation of unreacted elemental sulfur, which ceases by 450 °C. Beyond this temperature, mesoporosity gradually decreases as the remaining species are removed. The pore size distribution (PSD) derived from Ar physisorption agrees well with the one calculated from CO₂ physisorption. Nevertheless, the CO₂ physisorption for the sulfur-carbon condensed at 400 °C indicates the presence of micropores, which is not confirmed by the PSD from the Ar physisorption. This discrepancy is potentially due to interactions that go beyond pure physisorption, as the incorporated sulfur interacts with the quadrupole moment of the CO₂ leading to an overestimation of the porosity.

Small- and wide-angle X-ray scattering (SAXS/WAXS) measurements reveal a major increase of the scattering profile intensity occurring in the micropores region (~0.6-4.0 nm^-1^) at 450 °C, concurrent with sulfur evaporation and the initial formation of the microporous framework. Above 600 °C, the scattering intensity continues to rise simultaneously with the partial graphitization of the amorphous carbon phase and gradual decomposition of sulfur chains covalently bonded to carbon. This process results in the formation of nanovoids between the edges of the pseudo-graphitic carbon layer edges, as confirmed by HR-STEM images of sulfur-carbons condensed at 700 and 800 °C (Figure S6). To gain a deeper understanding of the structural evolution of sulfur-carbons, the SAXS patterns were analyzed using appropriate fitting models (Table S3 and Figures S9a-g). A Q^-4^ power law additionally to the Teubner-Strey model was applied to fit the Porod’s slope and WAXS region at high Q-values respectively.^[1, 3]^ The Teubner-Strey model gives structural information about the sulfur-carbons, revealing the increasing lamellar d-spacing with rising condensation temperatures, in agreement with HR-STEM observations. The increased intensity in the microporous region requires the inclusion of the Debye-Bueche model additionally to a Q^-2^ power law.^[2-3, 46]^ The Debye-Bueche model provides information about the microporous system by characterizing the average pore size.


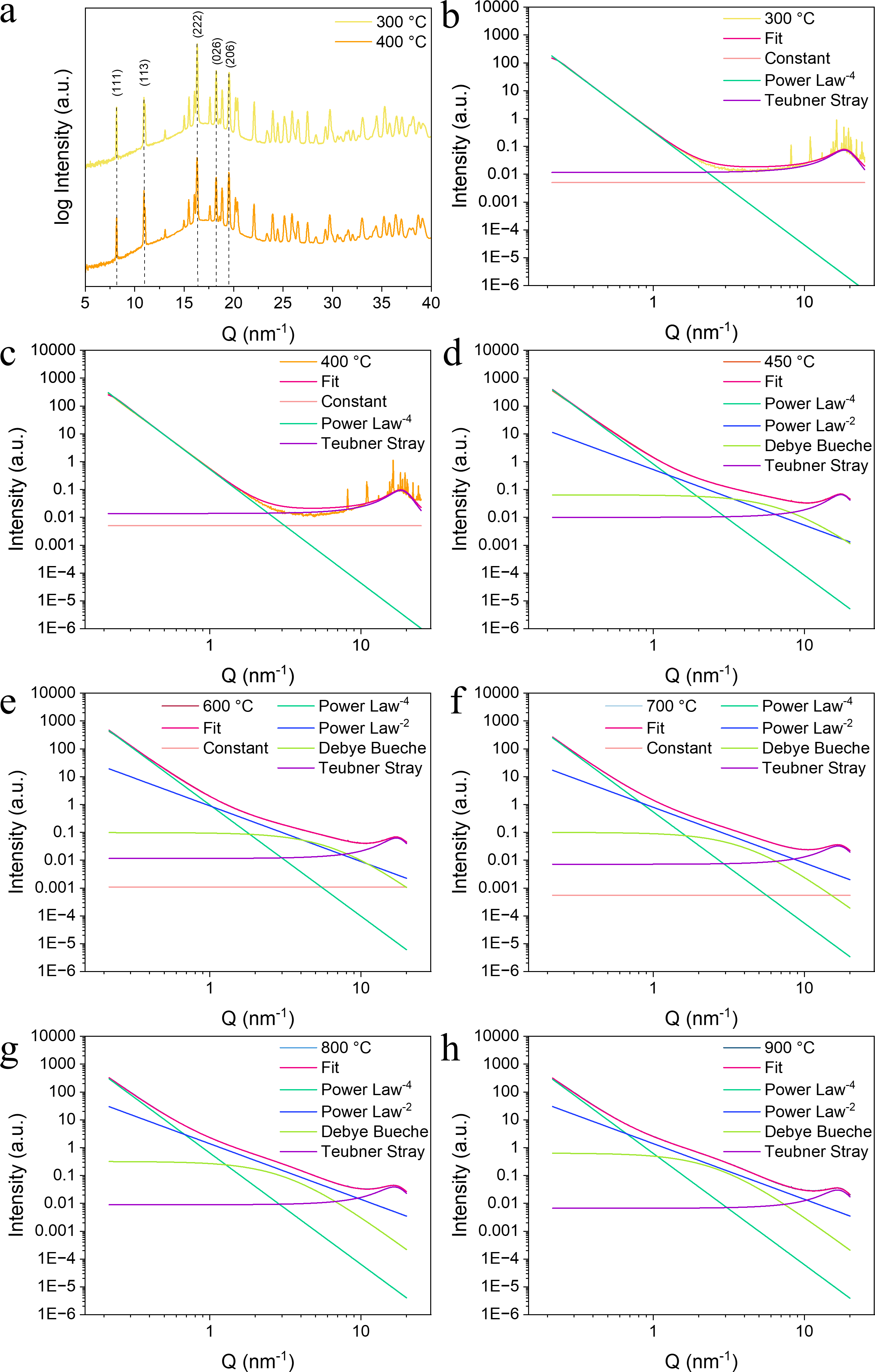


**Figure S9.** a) Indication of the five most intense peaks of the crystalline sulfur phase at 8.2, 10.9, 16.3, 18.2 and 19.5 nm^-1^ in agreement with reported values.^[47-48]^ b-h) SAXS/WAXS measurements of sulfur-carbons thermally condensed between 300 and 900 °C, showing measured data, fitting curves, and their respective components.

­­­
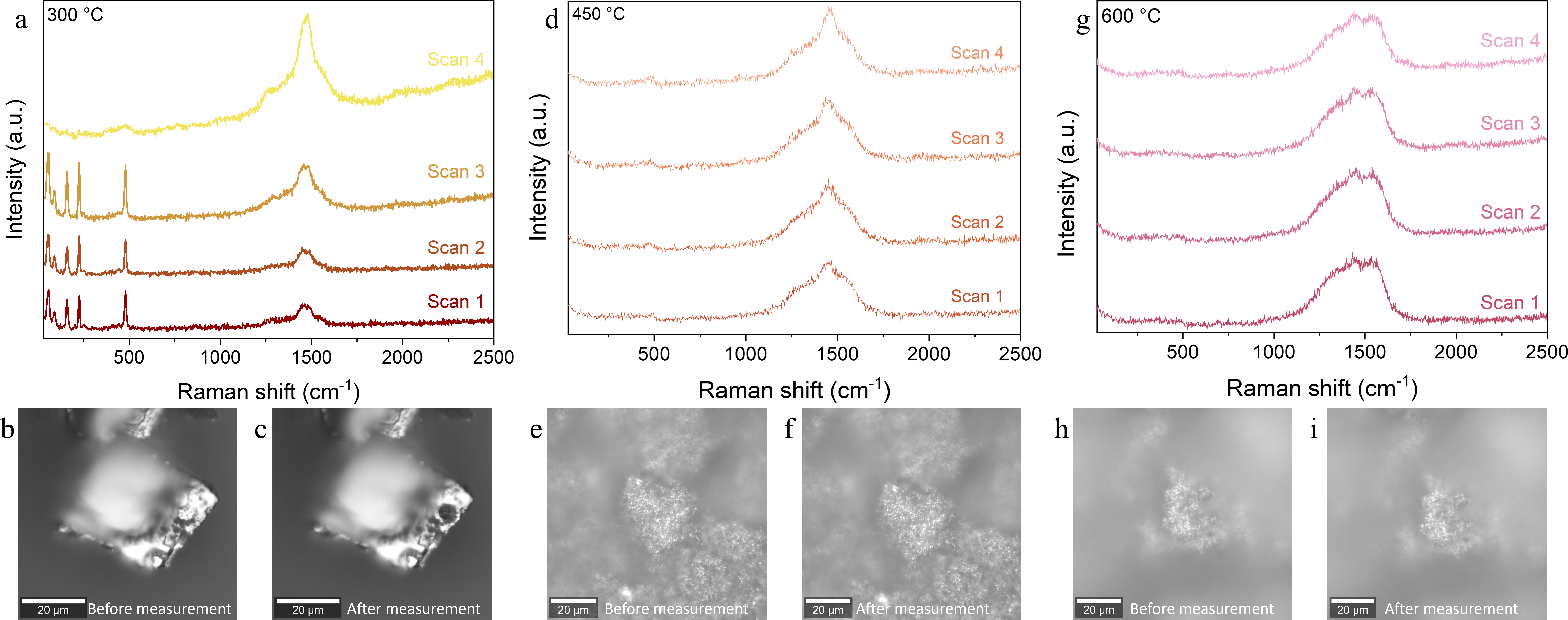


**Figure S10.** Raman spectra (laser power: 0.5 mW, acquisition time: 5 s) recorded consecutively from the same spot, illustrating the stability of sulfur-carbons condensed at 300, 450 and 600 °C. a) Spectra of the sulfur-carbon thermally condensed at 300 °C, with corresponding optical images b) before and c) after the measurement with distinct sulfur instability. d) Spectra of the sulfur-carbon thermally condensed at 450 °C, displaying minimal decomposition, seen from comparison e) before to f) after the measurement. g) Spectra of the sulfur-carbon thermally condensed at 600 °C, demonstrating no observable decomposition, from comparing optical images h) before and i) after the measurement.

X-ray photoelectron spectroscopy (XPS) analysis of the C 1s spectra show a decrease in intensity of the C–S peak and a shift towards lower binding energy with increasing condensation temperature, reflecting a transition to a less electronegative environment of carbon atoms suggesting thermal cleavage of less stable densely-packed long-chain polysulfides, resulting in the formation of shorter sulfur chains and low-molecular-weight thiols (Figure S11b).^[43, 49-50]^ This alludes to the possibility that the polysulfide chains shorten and their structure becomes more carbon-rich. However, from 700 to 900 °C, the C–S binding energy shifts back to higher values, indicating that the formed polysulfides become closely associated with more electronegative graphitic carbons rather than sp^3^-hybridized carbon atoms.

XPS peak fitting of the S 2p spectra is challenging due to the close overlap of multiple sulfur-sulfur and sulfur-carbon components, making the analysis extremely sensitive and requiring precise evaluation of subtle changes in peak shape (Figure S11c).^[51]^ However, comparing relative changes in the peak position and shape across different temperatures can provide more meaningful insights into the actual chemical transformations occurring in the material, rather than attempting to assign each deconvoluted peak to a specific component. At 300 °C, the central signal of the S 2p peak consists of two main doublets, designated to the central S of the polysulfides covalently bonded to carbon at 163.6 eV and unreacted sulfur at 164.1 eV. The lower binding energy of the former corresponds to organic thioether (R–S–R′) and/or aliphatic thiol (R–SH) groups, which are likely formed through electrophilic addition of sulfur radicals to olefinic C=C double bonds during inverse vulcanization and allylic hydrogen abstraction by sulfur radicals leading to thiol formation.^[52-55]^ The latter one may correspond to unreacted sulfur molecules, present in the form of mono- or disubstituted open polysulfide chains, and unreacted S_8_, as seen from SAXS/WAXS measurements (Figures 2c and d).^[52-53, 56]^ At 800 and 900 °C, the diminishing of the high binding energy signal (>166.5 eV) associated with oxidized sulfur, coincides with the emergence of a thiophene peak.^[53-55, 57-60]^ This implies that the sulfoxide groups may undergo reduction to sulfur-containing heterocycles due to graphitization at high temperatures.^[61-62]^


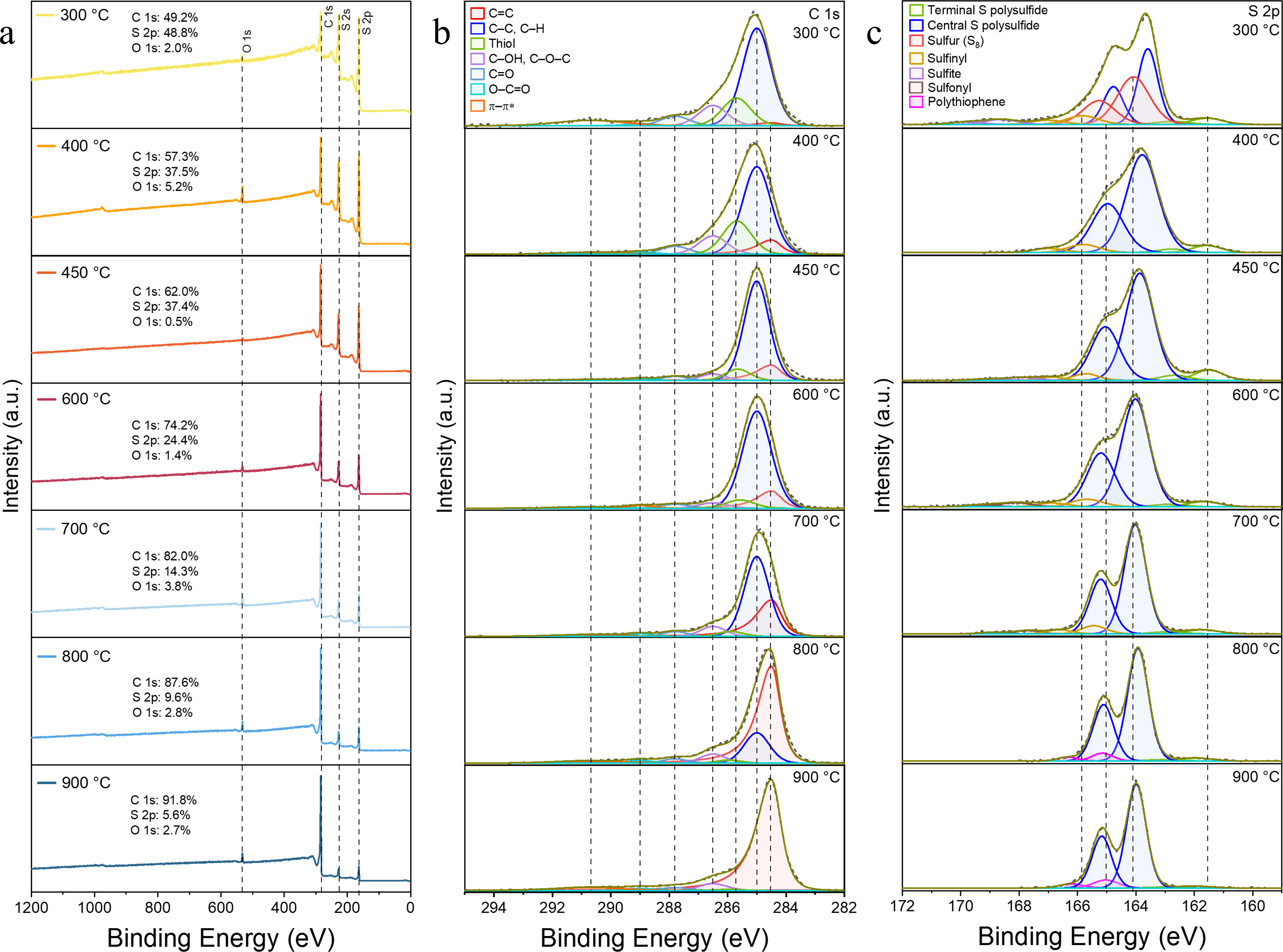


**Figure S11.** XPS analysis of sulfur-carbons condensed between 300 and 900 °C showing the a) survey spectra with enhanced regions with included XPS fits of b) C 1s and c) S 2p, highlighting the transition from a predominantly sp^3^-hybridized carbon-phase at lower condensation temperatures to a primarily sp^2^-hybridized carbon-phase at higher condensation temperatures. This evolution corresponds to the conversion from an amorphous to a pseudo-graphitic structure, as observed in HR-TEM. The S 2p region also reveals a more complex shortening of the sulfur-chains with increasing condensation temperature.


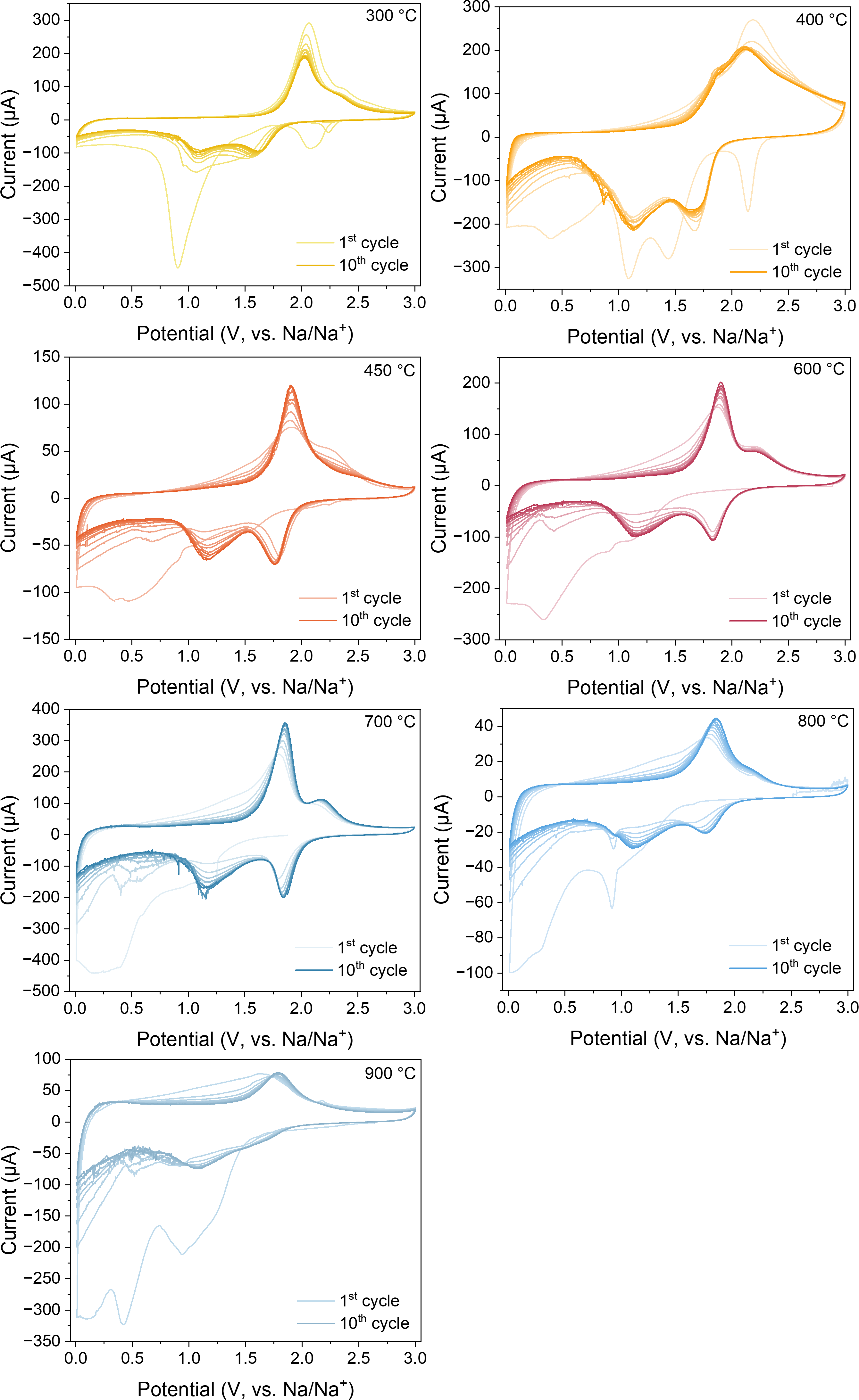


**Figure S12.** Cyclic voltammetry (CV) curves (scan rate: 0.1 mV s^-1^) of sulfur-carbons condensed between 300 and 900 °C. Each graph compares the first 10 cycles, showing the evolution of the redox peaks and current response as a function of applied potential.


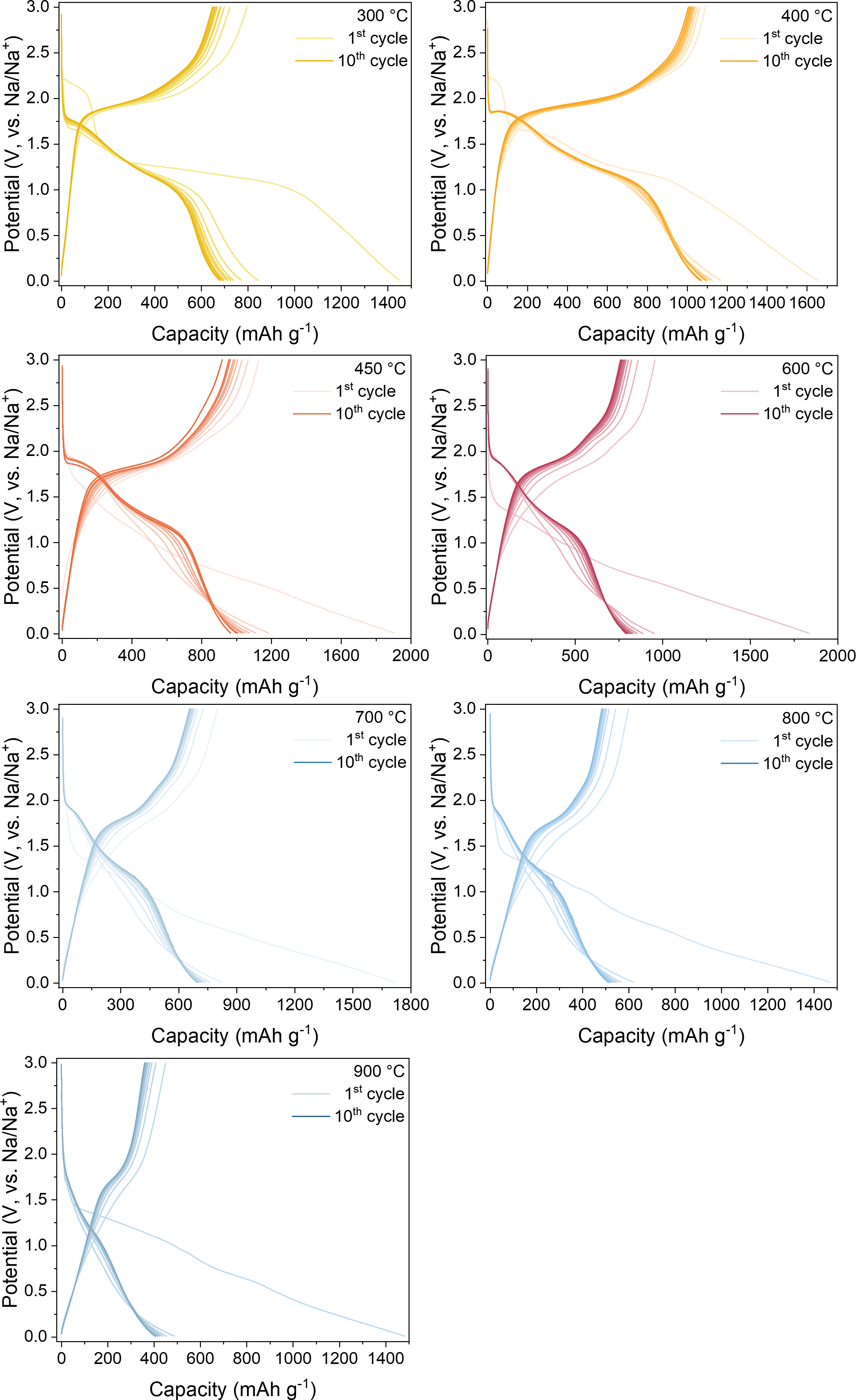


**Figure S13.** Galvanostatic charge-discharge (GCD) profiles of sulfur-carbons condensed between 300 and 900 °C at a charge rate (C-rate) of 0.1 C. Each plot compares the first 10 cycles, showing the evolution of voltage profiles and capacity retention during cycling.


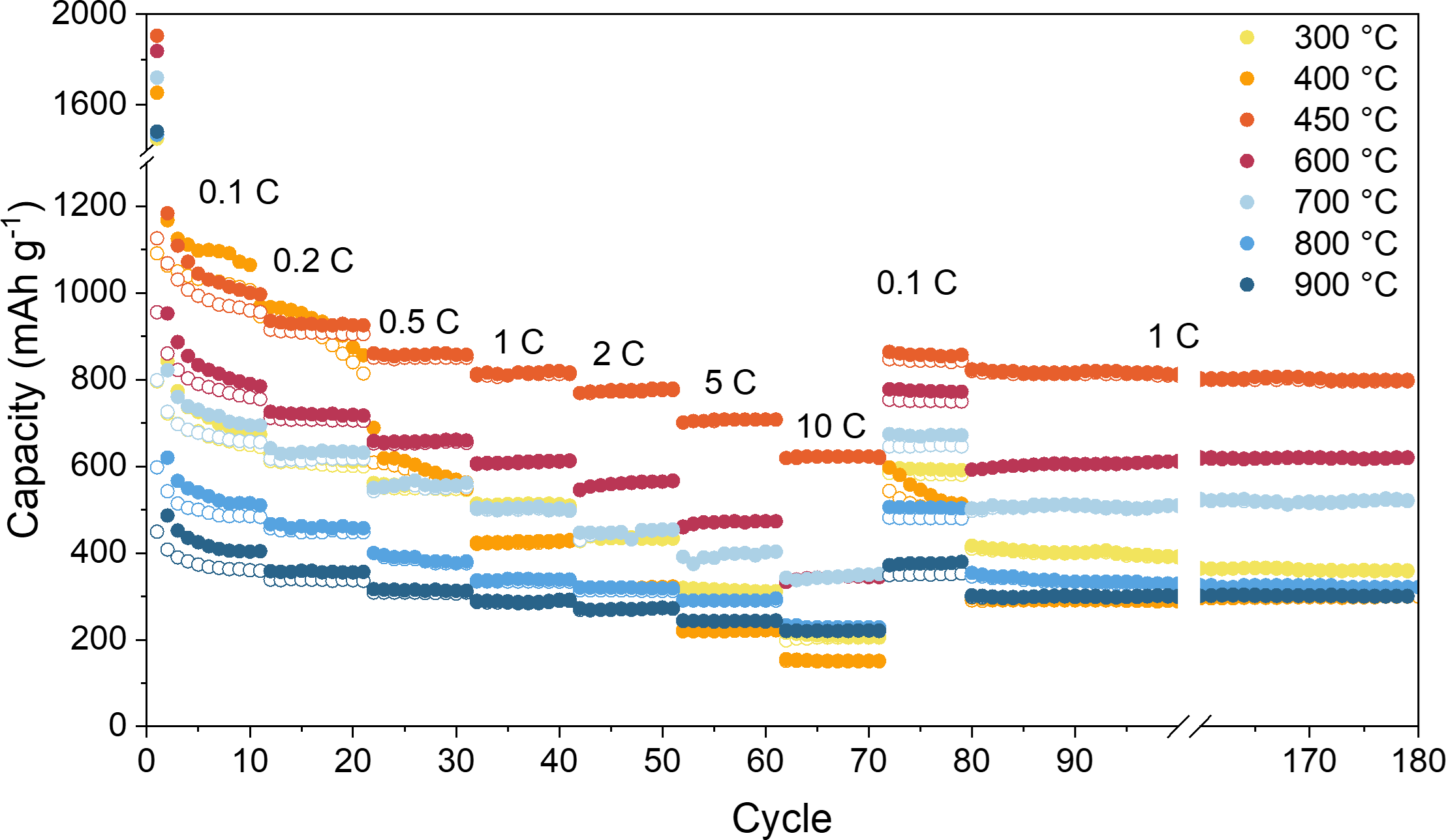


**Figure S14.** Rate capability and cycling performance of sulfur-carbon condensed between 300 and 900 °C. The electrodes were tested by GCD at various C-rates from 0.1 to 10 C and then reverted to 0.1 and 1 C to evaluate their stability and recovery over 100 cycles. The capacity and C-rate performance vary with thermal condensation temperature, demonstrating the impact of condensation temperature on electrochemical performance.

To investigate the evolution of sulfur species and the role of confinement on the electrochemical conversion mechanism, *ex-situ* XPS was conducted on sulfur-carbons condensed at 300 °C and 600 °C, across different states of charge (Figure S15). Upon discharge to 1.4 V, the XPS spectrum of the sulfur-carbon condensed at 300 °C reveals three distinct components, sulfur (S^0^) at 163.6 eV, sodium polysulfides (Na₂Sₓ, 2 ≤ x ≤ 4) at 162.3 eV, and sodium sulfide (Na₂S) at 160.5 eV, with the latter two appearing in approximately equal signal intensities. The prominent early Na₂S signature implies that the sulfur phase is highly accessible in a weakly protected, carbon-deficient matrix, enabling fast progression from S^0^ to Na₂Sₓ and subsequently to Na₂S. Upon further discharge to 0.01 V, the Na₂S signal intensifies, while the polysulfide and S^0^ features diminish, consistent with progressive reduction to Na₂S. However, even at full discharge, residual S^0^ and polysulfide signals remain, likely because the sulfur-rich cathode provides limited conductive sites, while agglomerated Na₂S isolates active sulfur and hinders complete conversion. This incomplete sulfur reduction reduces overall capacity and explains limited sulfur utilization in hybrid sulfur carbons synthesized at low temperatures. Upon charging to 3 V, the Na₂S signal disappears, suggesting reversible oxidation of accessible Na₂S into higher-order polysulfides and S^0^.

In the case of the sulfur-carbon condensed at 600 °C, similar sulfur species are present at 1.4 V, but the relative intensity of the Na₂S is significantly lower compared to the signal of reduced polysulfides. The weaker Na₂S signal at 1.4 V is consistent with delayed Na₂S precipitation in confined domains, where early reduction is dominated by conversion of S⁰ to Na₂Sₓ, whereas the Na₂S signature emerges only at deeper discharge. Upon full discharge at 0.01 V, Na₂S becomes dominant and S⁰ is nearly absent, indicating an almost complete conversion of the confined sulfur. Upon charging to 3 V, the Na₂S signal reverses fully, with only the central sulfur and polysulfide components remaining, which evidences high reversibility and ready sulfur access due to its homogeneous dispersion around conductive carbon sites.

Comparison between the non-cycled pristine sulfur-carbon electrodes and those at different state of charge offers further insight into chemical evolution of the sulfur phase during cycling. Unlike the cathode prepared at 600 °C, which shows negligible alterations in the position of central S peak at 164.0 eV during cycling, the 300 °C sulfur-carbon demonstrates significant shift of S–S peak to higher binding energy. The peak shift reveals transition of sulfur phase to a dominant S^0^ state which can be attributed to irreversible electrochemical cleavage of sulfur chains, which in sulfur rich state and absence of stable confined environment leads to reformation of sulfur during oxidation to more energetically favorable S^0^. This chemical reorganization is accompanied by segregation of larger sulfur domains, as corroborated by EDX mapping (Figure S19a-c), and highlights the critical role of confinement in maintaining reversible chemistry and long-term cycling stability.


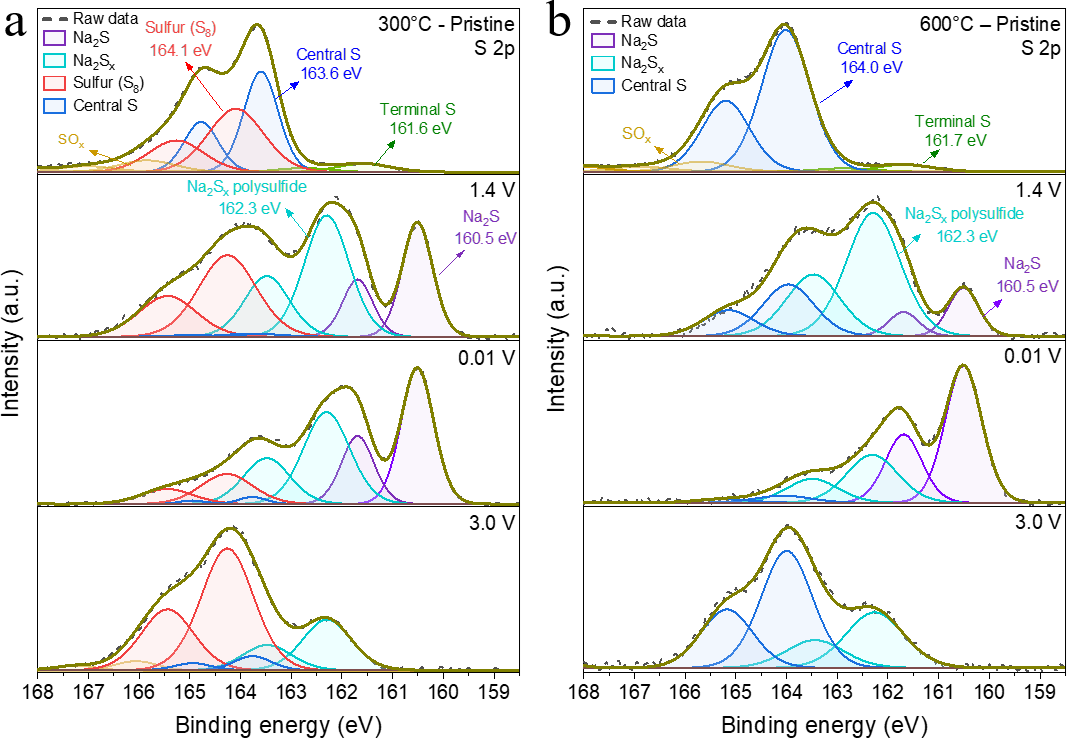


**Figure S15.** Ex-situ XPS analysis of pristine sulfur-carbons and at different states of charge of the sulfur-carbons condensed at a) 300 and b) 600 °C.

Electrochemical impedance spectroscopy (EIS) was conducted to investigate the behavior of the interfacial resistance at different charge states (Figure S16a-c). For the sulfur-carbon condensed at 300 °C, during sodiation the total impedance decreases between 3.0 V and about 1.0 V, which can be ascribed to the formation of soluble Na_2_S_x_ (2 ≤ x ≤ 4) species that improve electrode wetting, enhance interfacial ion transport, and lower the charge transfer barrier (Figure S16a). Near 1.0 V, the spectra develop a reproducible second semicircle and the overall resistance increases, consistent with the onset of solid-state conversion to Na₂S (Figure S17). In the sulfur-carbon condensed at 300 °C, weaker sulfur confinement favors non-uniform Na₂S nucleation and partial surface coverage, which reduces electrochemically active area and increases contact resistance, thereby producing the pronounced impedance growth. In contrast, for the sulfur-carbons condensed at 600 and 900 °C, shorter sulfur species are better confined within a more conductive carbon matrix, resulting in the formation of Na₂S as finely dispersed nanodomains that preserve electronic percolation and ion transport, hence the additional time constant is less prominent, and the impedance increase is small.

At 0.5 V, the impedance response diverges with condensation temperature. For the cathode materials condensed at 600 °C, and especially the 900 °C, the high-frequency semicircle grows, which we assign to thickening of the electrode-electrolyte interphase on the more exposed and conductive carbon framework. This effect is strongest at 900 °C, where sulfur is strongly depleted and CV shows the absence of long-chain polysulfide reduction, leaving a carbon-dominated surface that is more prone to electrolyte reduction and SEI formation (Figure 4a, b, and S12). In contrast, the 300 °C electrode is already passivated by non-uniform Na₂S deposits and contains less effective conductive carbon, so additional sodiation yields little new interphase, while the net resistance remains nearly unchanged.

During desodiation, the 300 °C sample essentially mirrors the sodiation trend, showing a shrinkage of the high-frequency semicircle at higher potentials and reaching minimal values of charge-transfer resistance by 3.0 V, consistent with oxidative removal of Na₂S and recovery of electronic/ionic pathways. Similarly, 600 °C electrode decreases steadily from 0.5 to 3.0 V, indicating efficient Na₂S oxidation. The 900 °C electrode displays small, nearly overlapping spectra across the 1.0 to 3.0V desodiation window, pointing to a stable, low-resistance interface.


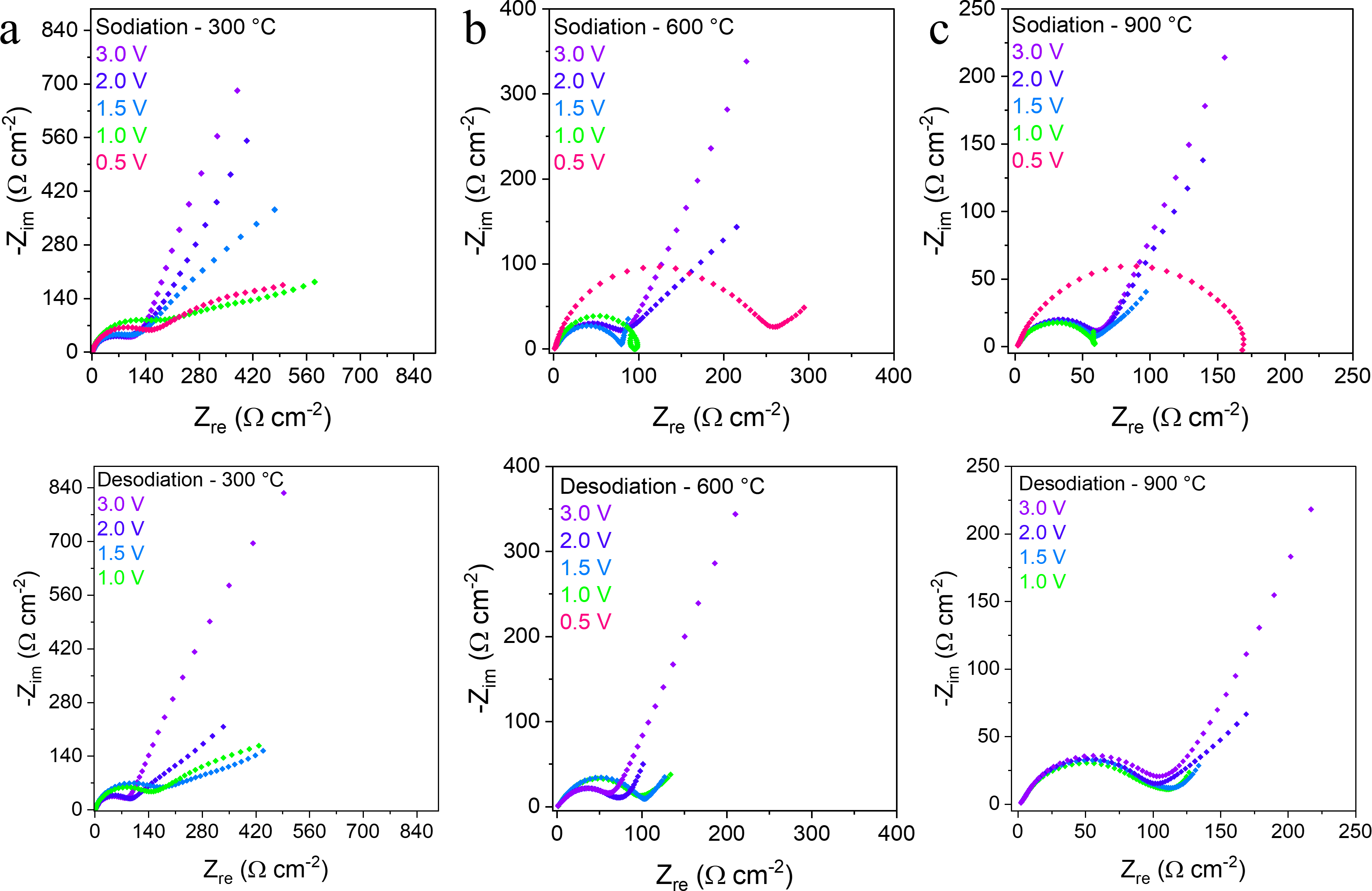


**Figure S16.** Nyquist plots from in situ EIS for sulfur-carbons condensed at a) 300, b) 600, and c) 900 °C, measured at 3.0, 2.0, 1.5, 1.0, and 0.5 V during sodiation and desodiation.


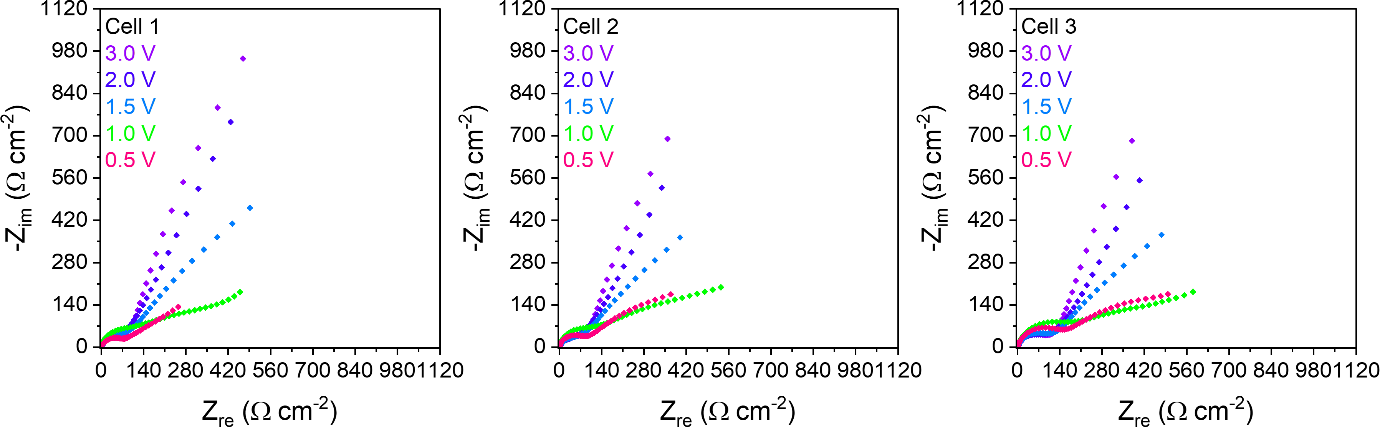


**Figure S17.** Reproducibility of in situ EIS for the 300 °C sulfur-carbon: Nyquist plots from three separately assembled cells.

After short-term cycling, the elemental maps show broadly homogeneous distributions of C, S, O, Na, and F, with a notable exception at 300 °C, where aggregated sulfur-rich clusters appear (Figure S18a-d). This observation can be ascribed to dissolution and subsequent shuttling of unconfined sulfur, leading to redistribution and localized accumulation of residual sulfur, forming the clustered patterns shown (Figure S18a, S mapping). In contrast, the sulfur-carbon synthesized at higher condensation temperatures exhibits a homogenous sulfur distribution, consistent with improved confinement and matrix integrity achieved through a gradual transition from sp^3^ to sp^2^ hybridization and formation of partially conjugated and locally graphitized carbon domains.


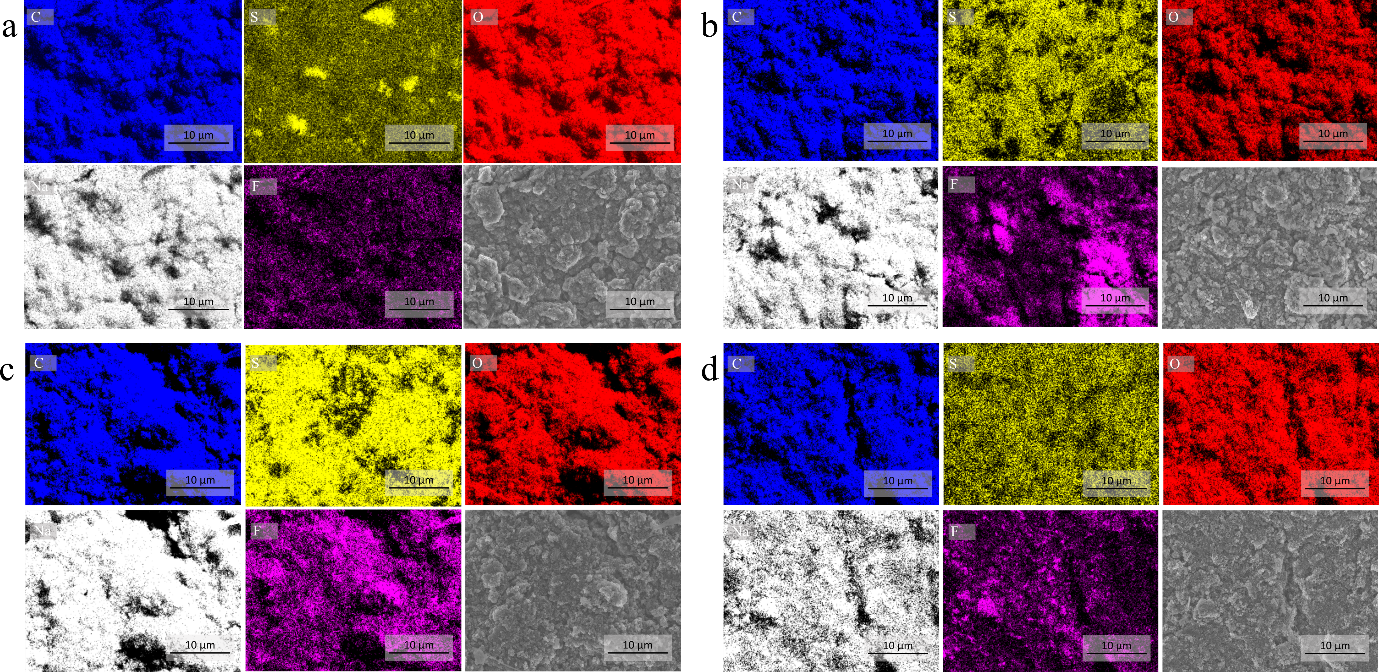


**Figure S18.** EDX elemental maps of post-cycling electrodes with sulfur-carbons condensed at a) 300 °C, b) 600 °C, c) 700 °C, d) 900 °C.

SEM images of the sulfur-carbon electrode before and after cycling (Figure S19a and b) confirm that the sulfur-carbon condensed at 600 °C maintains its overall integrity after long-term cycling, with no evidence of severe cracking, detachment, or catastrophic collapse. However, cycling induces noticeable morphological evolution, specifically particle coarsening, surface roughening, and the formation of larger aggregated domains. This suggests that while sulfur remains well confined (as supported by electrochemical data), prolonged electrochemical cycling still promotes some degree of surface reorganization. These changes can be explained by the gradual build-up of a thin cathode-side interphase that acts like a glue through Na, O, and F containing inorganic and polymeric species, together with sodium salt deposition and limited dissolution and reprecipitation of confined sulfur, which encourage small grains to cluster (Table S5). In parallel, repeated conversion reactions impose cyclic expansion and contraction, leading to slow compaction that presses neighboring particles into contact and promotes coalescence.


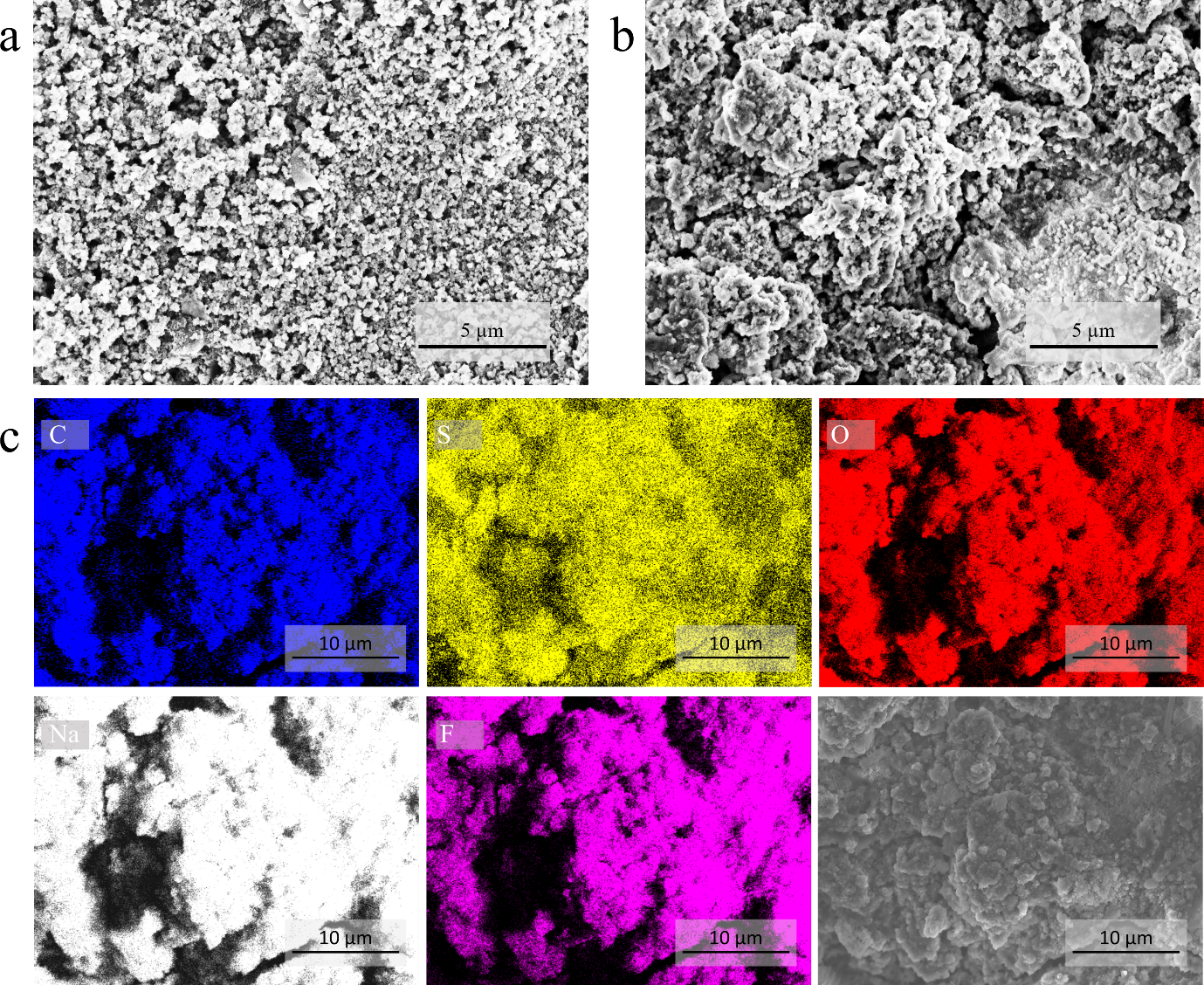


**Figure S19.** SEM images of the electrode of the sulfur-carbon synthesized at 600 °C at a a) non-cycled state and b) after 500 GCD cycles with additional e) EDX mapping of C, S, O, Na, and F.

High-resolution scanning transmission electron microscopy (HR-STEM) images of both the sulfur-carbon powder and the post-cycling electrodes reveal only minor structural alterations in the carbon matrix after long-term cycling (Figure S20a). The short-range order characteristics of the carbon nanodomains is largely preserved, indicating that the partially graphitized structure remains stable under electrochemical operation. Importantly, no signatures of large crystalline Na_2_S domains or sulfur-rich agglomerates are detected, suggesting that conversion reactions do not lead to macroscopic segregation of sulfur species. Elemental mapping further shows a homogeneous distribution of carbon and sulfur across the electrode, with no localized sulfur enrichment (Figure S20b-d). Together, these observations indicate that sulfur remains well confined within the carbon matrix after extended cycling. Such nanoscale confinement supports electrochemical reversibility, since it suppresses polysulfide shuttling, limits irreversible side reactions, and helps preserve the porous electrode architecture. Overall, the HR-STEM and elemental mapping results demonstrate the structural robustness of the sulfur-carbon material and provide strong evidence that the designed nanostructure stabilizes sulfur species during prolonged cycling.


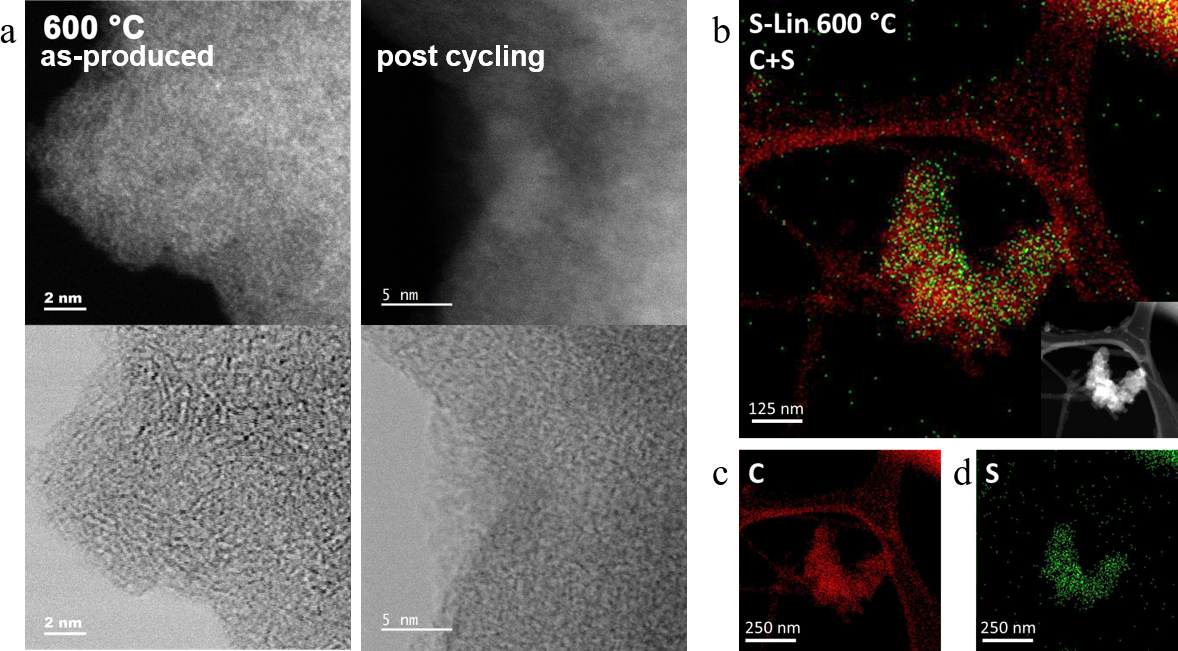


**Figure S20.** a) Dark and bright field HR-STEM images of the sulfur-carbon condensed at 600 °C: as produced non-cycled material and post cycled electrode, and b) elemental mapping in HR-TEM with inlay and individual mappings of c) C and d) S.

The galvanostatic intermittent titration technique (GITT) profiles show a stable, decreasing discharge potential until reaching 1.0 V, followed by a sharp drop (Figures S21a and S22a). From these curves, Na-ion diffusion coefficients (D_Na_) indicate a two-phase discharge and charge profile (Figures S22b and S23b) with D_Na_ decreasing to a minimum at 1.3 V, consistent with the reduction of long-chain polysulfides to shorter-chain polysulfides and Na_2_S, which reduces ion mobility.^[63]^ At this stage, D_Na_ is primarily limited by solid-state diffusion as Na_2_S_2_ and Na_2_S formation becomes dominates. Sulfur-carbons condensed at 300 and 400 °C exhibit the lowest D_Na_, which increases with higher thermal condensation temperatures due to formation of shorter polysulfide chains that facilitate Na-ion diffusion. During charge, D_Na_ decreases at 1.3 V as Na_2_S converts to short-chain polysulfides (Figures S21c and S22c), followed by a sharp increase at 1.9 V as short-chain polysulfides convert back to longer chains.


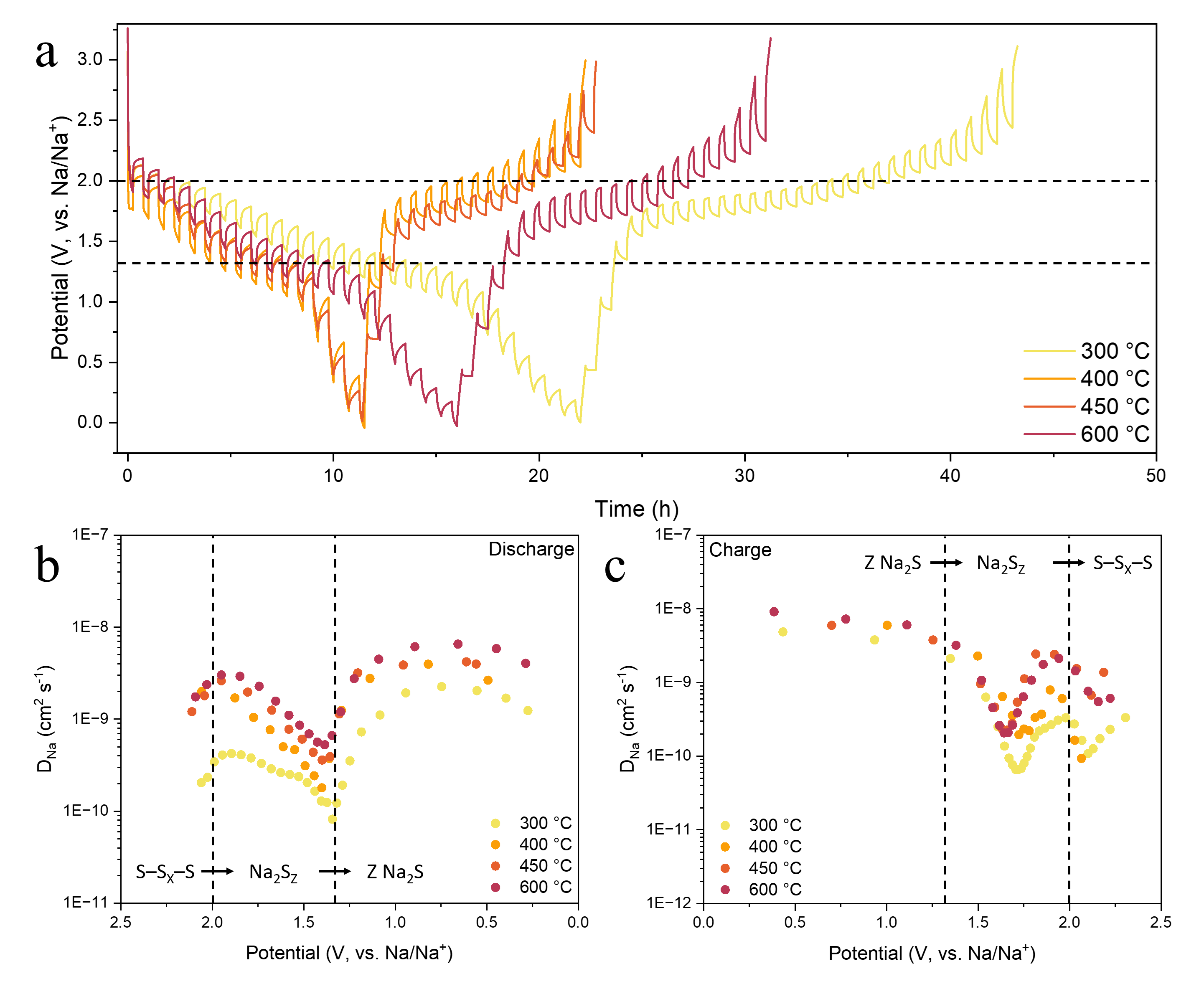


**Figure S21.** a) GITT profiles of sulfur-carbons condensed between 300 and 600 °C during discharge and charge. b) Na-ion diffusion coefficient (D_Na_) as a function of potential during discharge, highlighting the transition from long-chain polysulfides to Na_2_S. c) Na-ion diffusion coefficient during charge, showing the conversion of Na_2_S to long-chain polysulfides. The lowest D_Na_ values during discharge are observed at 1.3 V, where solid-state diffusion limitations dominate due to the formation of Na_2_S_2_ and Na_2_S


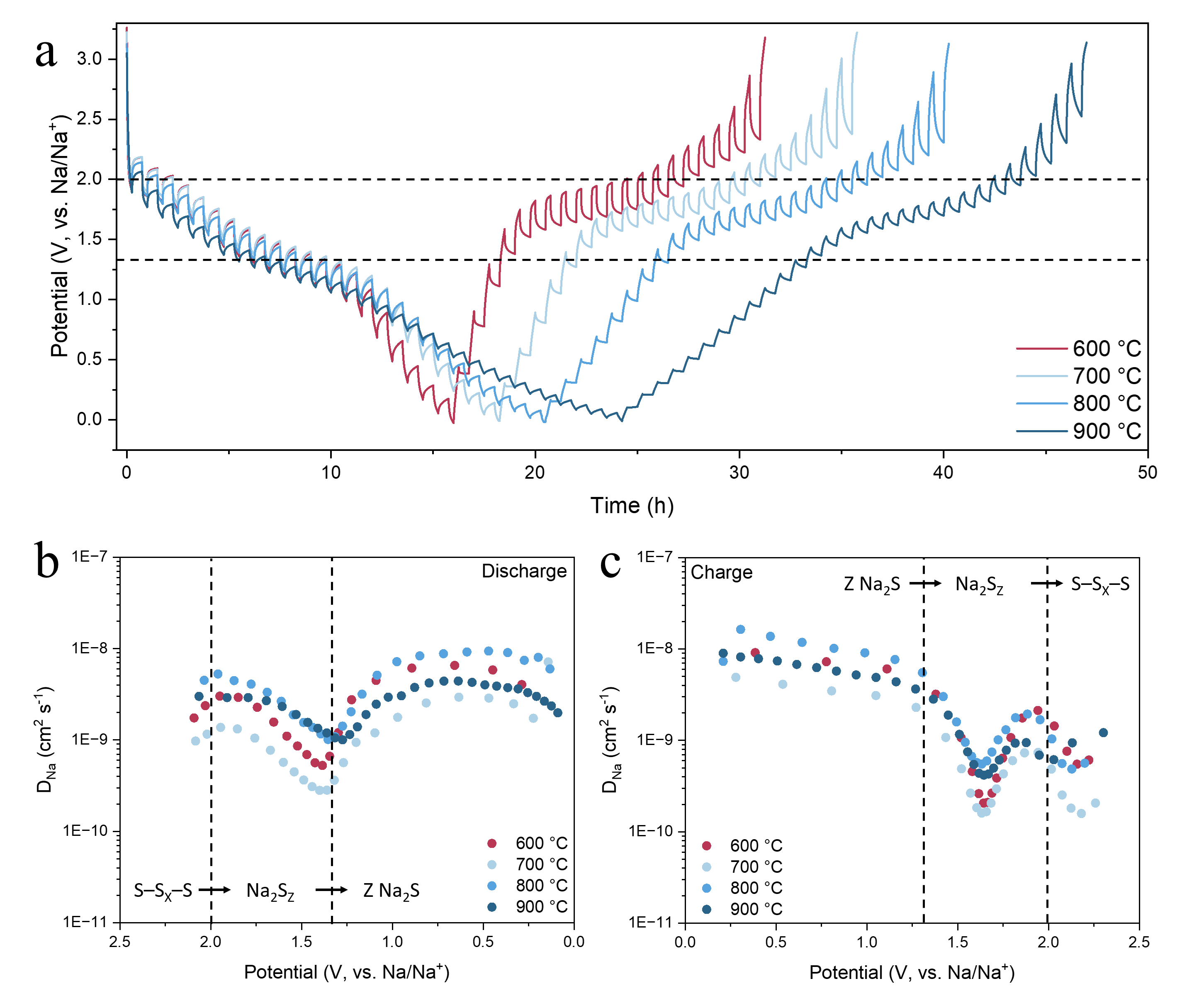


**Figure S22.** a) GITT profiles of sulfur-carbons condensed between 600 and 900 °C during discharge and charge. b) Na-ion diffusion coefficient as a function of potential during discharge, illustrating the phase transition from long-chain polysulfides to Na_2_S. c) Na-ion diffusion coefficient during charge, highlighting the conversion of Na_2_S to long-chain polysulfides. Higher condensation temperatures lead to increased Na-ion diffusion coefficients, facilitating better electrochemical performance.

Using density functional theory (DFT) calculations, we investigated the interactions between graphene sheets characterized by intrinsic vacancies and exposed edges, and sulfur atoms. Starting from a defective, pure graphene layer configuration (Model 1), sulfur atoms were incrementally introduced (Figure S25a) to calculate the enthalpy of formation and construct a convex hull diagram that captures the thermodynamic progression of sulfur incorporation into Model 1 (Figure S30). This approach reveals the energetically preferred graphene-sulfur configurations that sodium may encounter during cycling (Table S8).


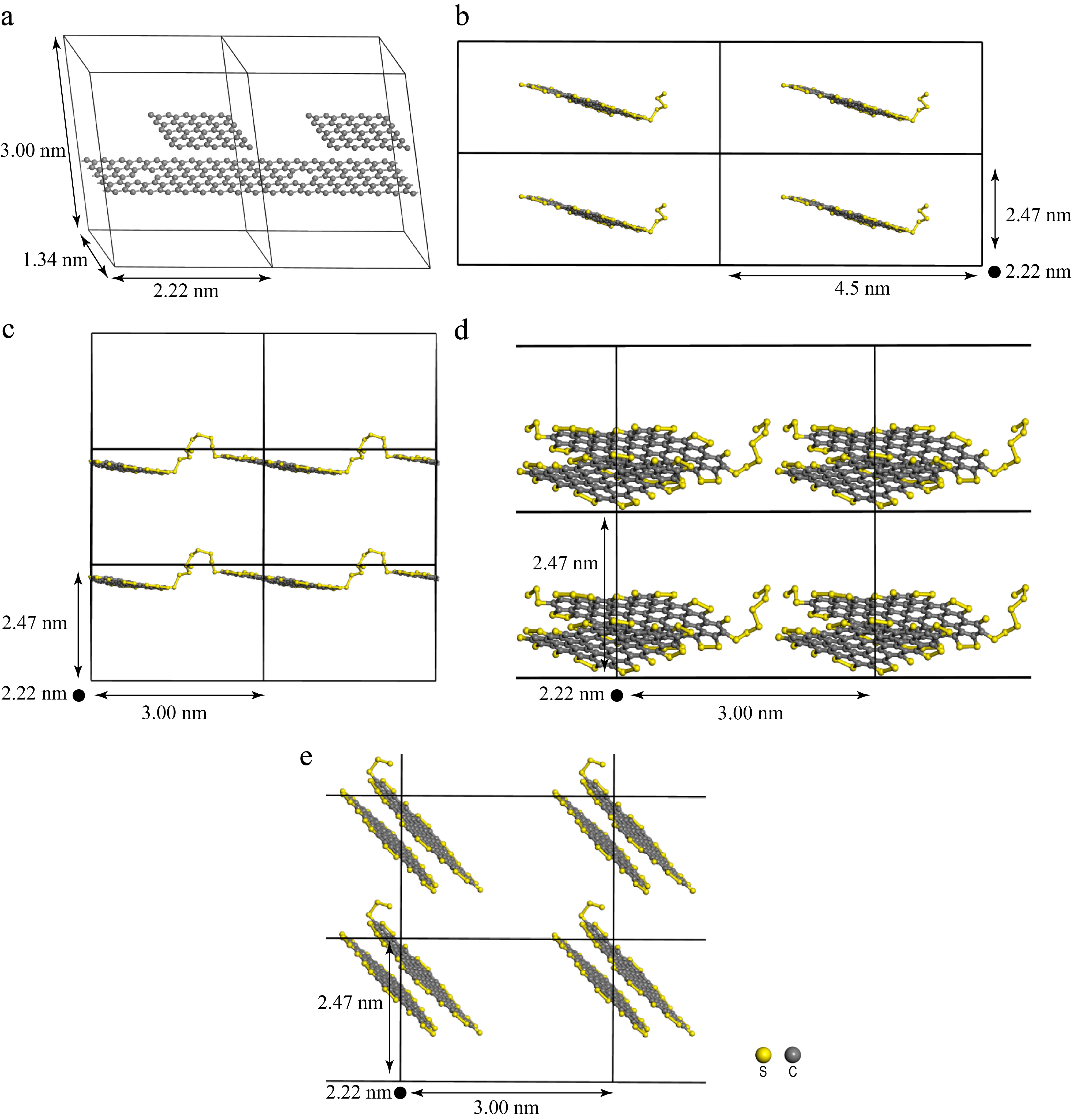


**Figure S23.** Models for DFT calculations: a) Model 1 b) Model iso-4S-chain representing an isolated sulfidated graphene sheet with a short sulfur chain consisting of four sulfur atoms. c) Model inter-8S-chain denoting a sulfidated graphene sheet that, due to periodic boundary conditions, effectively behaves as two interacting sulfidated graphene sheets with an eight-atom sulfur chain confined between them. d) Model 2-inter-8S-chain introducing an additional sulfidated graphene layer, creating a configuration with four interacting sheets and further increasing confinement around the same eight-atom sulfur chain. e) Model 2-inter-2S-chain featuring a similarly confined geometry as 2-inter-8S-chain, but encloses a much shorter sulfur chain composed of only two sulfur atoms.


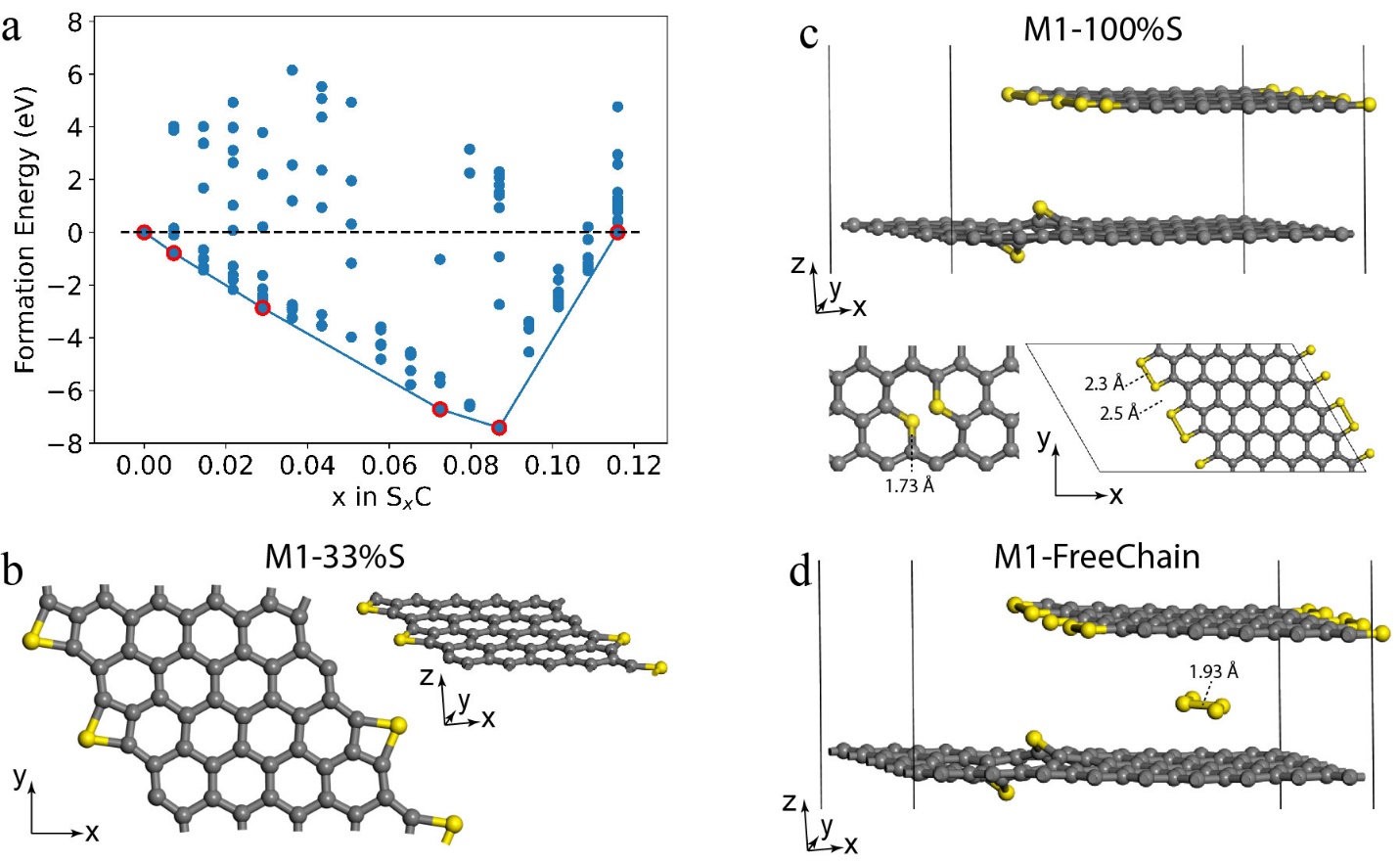


**Figure S24.** a) Convex hull diagram illustrating the introduction of sulfur in Model 1 b) Stable configuration at S₀.₀₃C, where sulfur occupies 33 at.% of the available defects (vacancies and edges) in Model 1, designated as M1-33%S c) Stable configuration at S₀.₀₉C, with sulfur occupying 100 at.% of the available defects, designated as M1-100%S d) Stable configuration at S₀._12_C, where sulfur occupies 100 at.% of the defects and includes a free sulfur chain of four atoms, designated as M1-FreeChain.

Sulfidation begins at the exposed carbon edges, resulting in a dilute sulfur distribution (Figure S25b). As the sulfur concentration increases, sulfur atoms begin to occupy graphene vacancies and progressively assemble along the edges, eventually saturating all available defect sites (Figure S25c). At higher sulfur loadings, small sulfur chains form and become stabilized either near edge-bound sulfur atoms or within the interlayer space between graphene sheets (Figure S25d). Calculated binding energies show that both sulfur and sodium have the strongest interaction at carbon edges, directing further development of the model (Table S9). Introducing Na atoms into the structures provides insight into their interactions within various sulfidated environments. To contextualize Na behavior across different levels of sulfidation, three sulfidated configurations were employed as host structures (Figure S25a-d and Table S11). This strategy allows comparison of Na interactions in a lightly sulfidated graphene environment, where some graphene defect sites remain accessible, with those in a fully sulfidated structure, where Na primarily interacts with the sp²-hybridized graphene surface or defect-bound sulfur. In addition, a fully sulfidated configuration containing a free sulfur chain (S₄) was examined to explore how Na interacts with the free S chain. The enthalpies of formation upon sodiation and voltage profiles are presented in Figure S25 below.


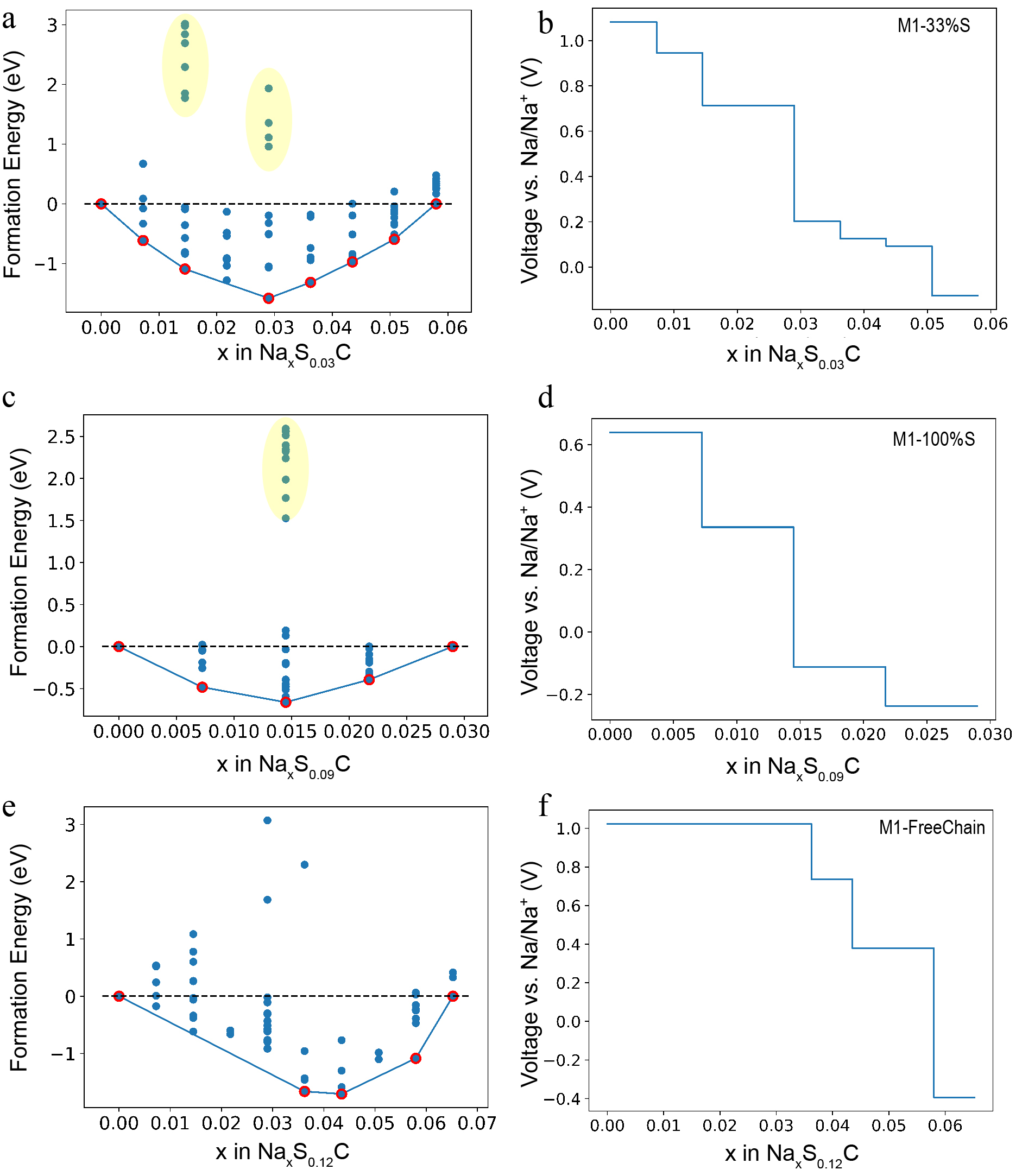


**Figure S25.** a, b) Convex hull diagram and voltage profile for the sodiation of the M1-33%S host. c, d) Convex hull diagram and voltage profile for the sodiation of the M1-100%S host. e, f) Convex hull diagram and voltage profile for the sodiation of the M1-FreeChain host. Enthalpy points highlighted in yellow represent configurations where S detaches from its stable positions (edges or vacancies) to form Na-S structures. The high positive energy of these points relative to the convex hull indicates that this process is energetically unfavorable.

We compare the voltage profiles of different models with pure defective graphene and compare them with the voltage profile resulting from sodiation of pure defective graphene (Figure S27).


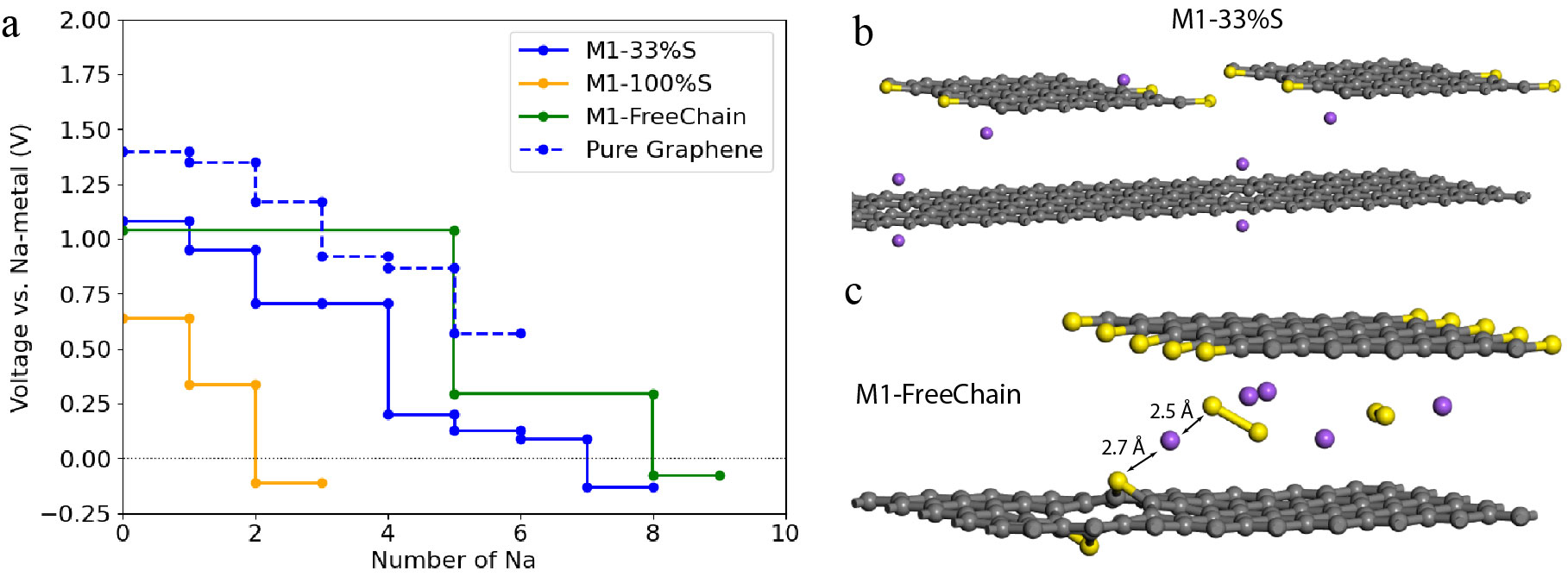


**Figure S26.** a) Voltage profiles for the sodiation of M1-33%S, M1-100%S, and M1-FreeChain models. For reference, the sodiation voltage profile of a pure defective graphene is also included (blue dotted line).^[64]^ Models during the sodiation process of b) M1-33%S, and pure defective graphene, and c) M1-FreeChain.

During sodiation of the lightly sulfidated host (M1-33%S), a sloping voltage profile is observed, indicative of a solid-solution Na insertion process. In this environment, Na-atoms attach to available edge and vacancy sites, becoming stabilized within the structure. This behavior is similar to that of sodiation in pure defective graphene, with the key difference being that sulfur occupies some of the low-energy binding sites, thereby reducing the number of accessible sites for Na and lowering the sodiation potential. In the fully sulfidated configuration (M1-100%S), all defect sites are occupied by sulfur, leaving minimal space for Na-atom incorporation. Only a limited number of Na-atoms can adsorb onto the surface at low potential, and additional sodiation rapidly results in negative potential relative to Na-metal, indicating thermodynamically unfavorable conditions. The introduction of a free sulfur chain (M1-FreeChain) alters this scenario. Unlike defect-bound sulfur, the atoms in free chains are more reactive and can dissociate upon Na insertion to form small Na-S clusters. A stable configuration formed at 1.1 V is found where Na-atoms are primarily coordinated by sulfur from the free chains, with additional stabilization from nearby defect-bound sulfur. This configuration also reveals a shift in the sodiation mechanism, from a solid-solution process with a sloping voltage profile to a first-order phase transition, characterized by the emergence of a voltage plateau. Throughout this investigation, the possibility of defect-bound sulfur detaching from the graphene to form larger Na-S clusters was repeatedly examined. In all cases, this process exhibited highly positive formation energies, suggesting it is thermodynamically unfavorable (yellow shaded points in Figure S26). These findings reinforce the conclusion that free sulfur chains, rather than defect-bound sulfur, are primarily responsible for the formation of Na-S reaction products.


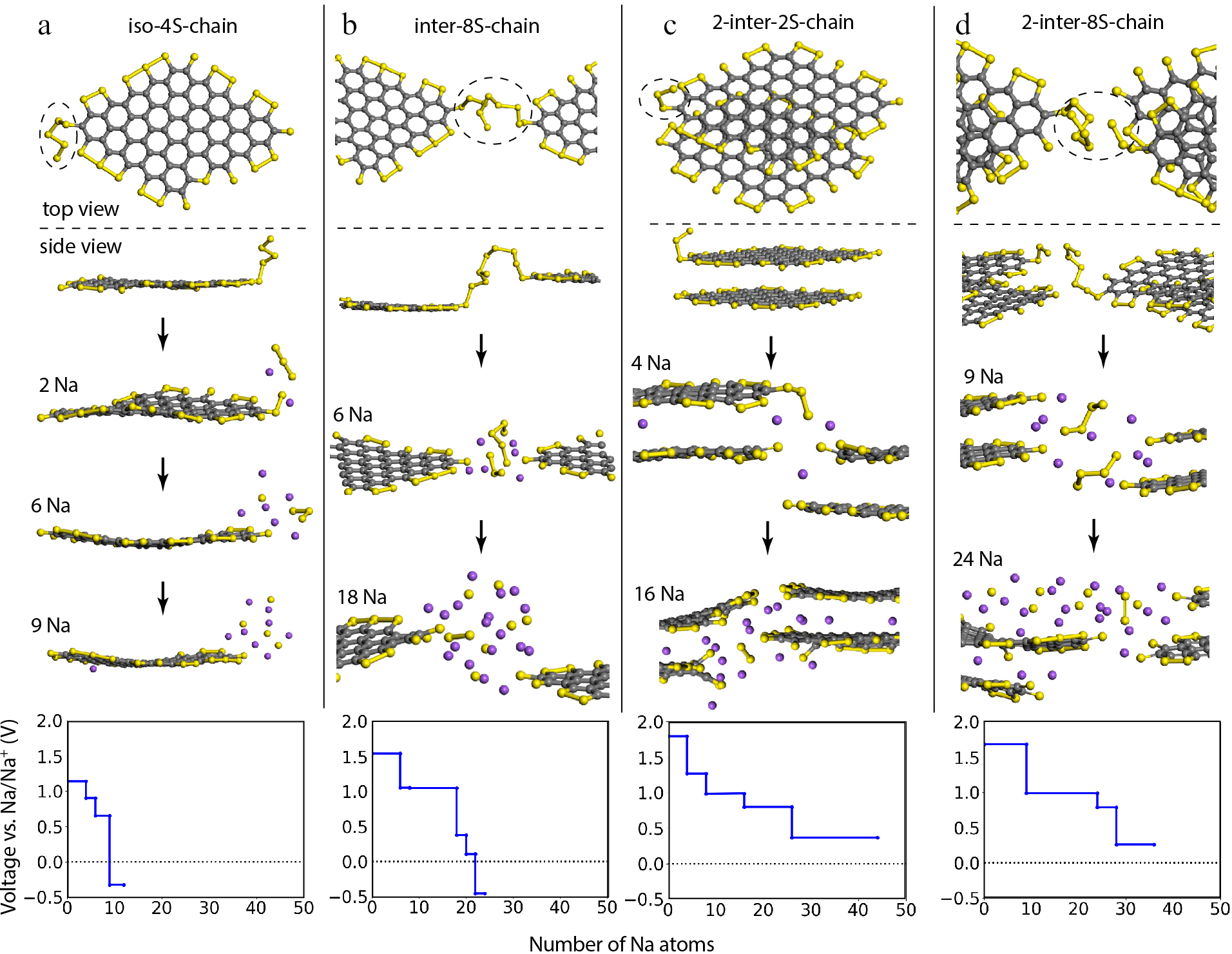


**Figure S27.** a) Sodiation of model iso-4S-chain: This represents an isolated sulfidated graphene sheet in a vacuum, with a short free S chain comprising four sulfur atoms attached. b) Sodiation of Model inter-8S- chain: This configuration features a single sulfidated graphene sheet where, due to periodic boundary conditions, the edges interact with each other. Each edge has a short free S chain consisting of four sulfur atoms attached (eight free chain atoms in total). c) Sodiation of Model 2-inter-2S chain: This configuration includes two sulfidated graphene sheets that, due to periodic boundary conditions, form an environment with four interacting sheet edges. The shorter free S chain, consisting of only two sulfur atoms, allows the sheet edges to stabilize in closer proximity. d) Sodiation of Model 2-inter-8S-chain: This configuration features two sulfidated graphene sheets that, due to periodic boundary conditions, create an environment of four interacting sheet edges. This configuration also contains eight free-chain atoms in total. All models were geometry-optimized as shown in Figure S26 and enthalpies of formation are presented in Figure S27.

Building on initial observations and a hypothesis regarding the role of free sulfur, more complex models are constructed to indicate how confinement and disordered carbon environments impact the sodiation profile. While replicating the exact proportions of these environments observed in real samples is not feasible, rendering the relative length of different regions in the voltage profile arbitrary, the sequence of sodiation events can be systematically analyzed. This methodology enables a detailed examination of the sodiation mechanism within these environments and provides insight into how mixed carbon structures influence the overall voltage profile.

To investigate the influence of spatial confinement and disordered carbon environments on the interactions between sulfur and sodium, four types of sulfidated graphene sheets with accessible sulfur chains were simulated (Figure S28). These models capture varying degrees of confinement and illustrate the interactions between sulfidated carbon sheets and sulfur chains available for incoming Na ions.

In the isolated sulfidated graphene sheet (iso-4S-chain), sodiation begins near the sulfur chains, which are cleaved at a calculated voltage of 1.14 V. As Na concentration increases, the sulfur chain continues to dissociate, forming a localized Na-S cluster with an approximate 1:1 Na:S ratio. Some edge-bound sulfur atoms contribute to stabilizing this cluster, coordinating with Na-atoms at interatomic distances of around 2.7 Å. In addition, a separate Na-atom adsorbs near a sulfidated graphene edge, independent of the cluster, at a lower voltage of 0.65 V. These relatively low sodiation potentials are likely due to the unconfined vacuum environment, which increases the surface energy cost of Na-S cluster formation. This behavior is reminiscent of alkali metal interactions with hard carbon materials, as reported in previous studies.^[64]^ A clearer two-step sodiation process is observed in the second model, which consists of two sulfidated graphene sheets connected by a longer sulfur chain comprising eight atoms (inter-8S-chain). The initial dissociation occurs at 1.54 V, reaching a Na:S ratio of 6:8, followed by a second voltage plateau at 1.05 V as the system approaches a 1:1 Na:S ratio. Compared to the isolated chain case, both plateaus are more extended, reflecting the increased availability and flexibility of the longer sulfur chain. The elevated plateau voltages can be attributed to the more confined environment, where the Na-S cluster receives additional coordination from the opposing sulfidated graphene edge. In the next configuration, further confinement is introduced by adding additional sulfidated graphene sheets, forming a geometry with four interacting sulfidated edges (2-inter-8S-chain). This results in a more complex and highly confined environment. Under these conditions, voltage plateaus are calculated at 1.68 V and 1.00 V. Notably, a new phenomenon emerges in this configuration: Na ions become stabilized not only within the Na-S cluster but also in the interstitial regions between the closely spaced sulfidated graphene sheets. This dual stabilization mechanism, within both the Na-S cluster and the confined interlayer space, further extends the length and stability of both sodiation plateaus. This concept is further examined in the final model, where the sulfur chain length is reduced and two interacting sulfidated graphene sheets are brought closer together (2-inter-2S-chain). The initial sodiation stage occurs at a relatively high potential of 1.79 V, with Na ions localizing near both the short sulfur chains and adjacent sulfidated carbon edges, consistent with the behavior observed in the previous model. Such a geometry exhibits a more disordered sodiation process, characterized by shorter and less distinct voltage plateaus, as Na-atoms continues to interact dynamically with both the sulfur chains and the confined environment.

Throughout this investigation, Na-ions do not intercalate the sulfidated graphene layers; instead, they stabilize between adjacent layer edges. Instead, they consistently remain outside the layers, stabilizing near sulfur atoms bound to edge sites. To maintain computational simplicity, the current models were intentionally designed as simplified and relatively open representations of real materials, which are expected to be significantly more intricate and disordered. This simplification raises important questions that could be addressed through future simulations using denser, more amorphous models. For example, how would increased confinement affect the second voltage plateau, which corresponds to the complete dissolution and reaction of free sulfur chains, a process that may require greater spatial freedom for Na insertion and clustering? Additionally, how would variations in defect density or sulfur content influence Na adsorption and the voltage profile? Understanding the role of structural disorder in modulating sodiation behavior and the distribution of active sites remains a key area for future investigation. These questions highlight the need to extend atomistic modeling toward more realistic representations of complex sulfur-carbon architectures in Na-S systems. These atomistic models, though simplified, reveal clear trends in how sulfur chain length and confinement influence Na-S interaction pathways and voltage behavior.


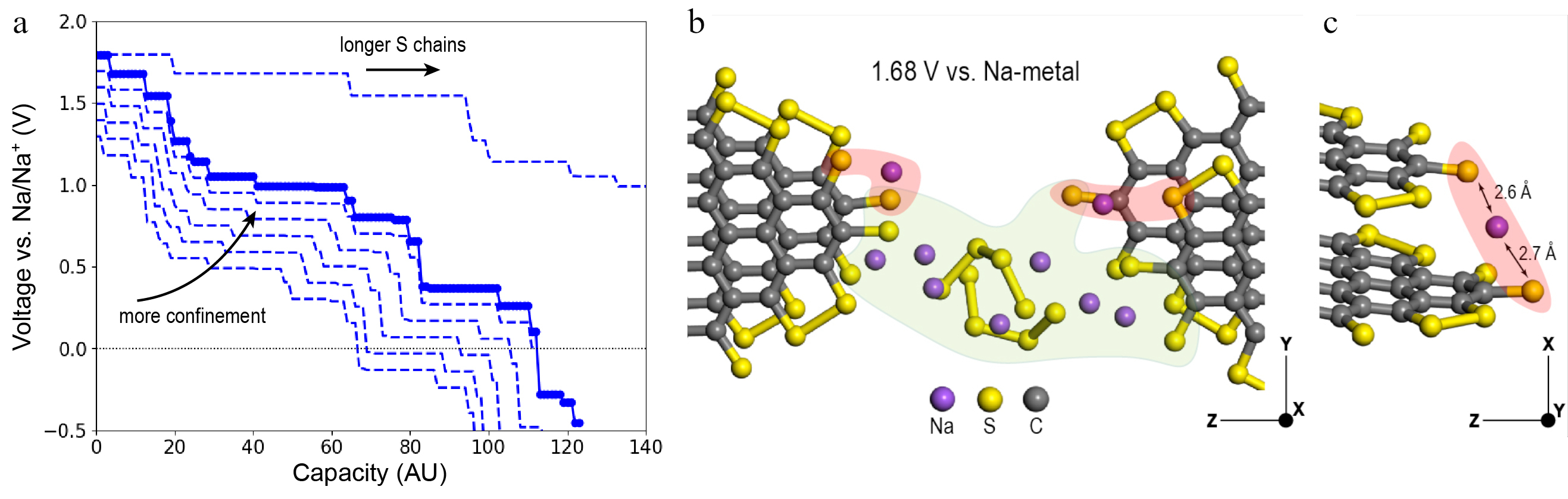


**Figure S28.** a) A schematic voltage profile based on the DFT calculations representing the trends we observed. Longer free sulfur chains contribute to greater capacity by enabling Na–S reactions and the formation of small clusters. Increased confinement raises the potential at which these reactions occur and introduces localized pockets that stabilize Na ions, thereby enhancing overall capacity. b) Representative configuration calculated at 1.68 V, featuring a small, stable Na–S cluster. The light green shaded area highlights atoms involved in the cluster, including edge-bound sulfur atoms that assist in coordinating the structure. Red shaded regions indicate a separate Na atom stabilized between two sulfidated graphene edges, spatially distinct from the main cluster. These regions do not indicate intercalation; rather, the Na atom is stabilized at the outer edges of the sulfidated layers. c) Close-up view showing the confinement-driven stabilization of a Na-atom situated near two adjacent sulfidated graphene edges, and not between (intercalation) the layers.


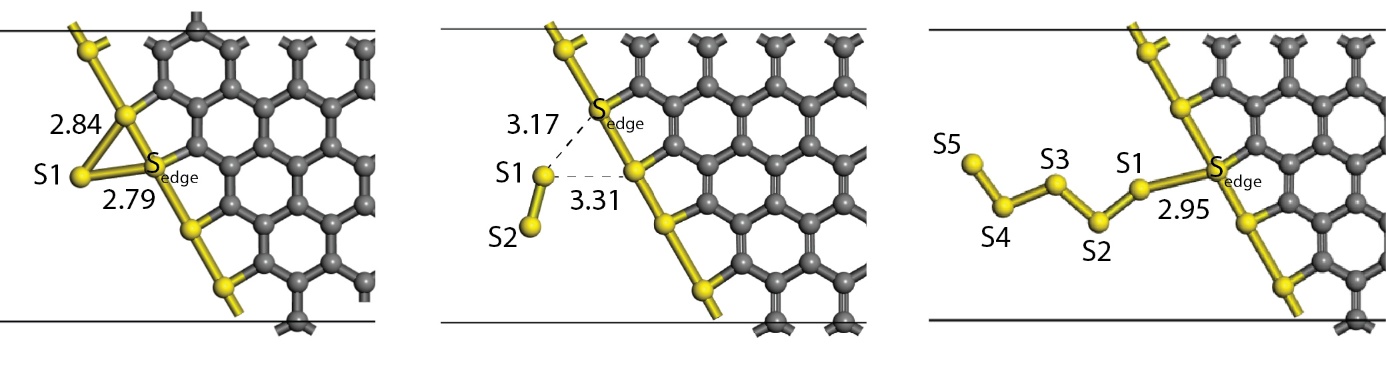


**Figure S29.** The optimized geometries of S chains attached to the sulfated edge for the following environments: (from left to right) chain length 1, chain length 2, chain length 5.

All anchoring strengths are negative, indicating that attachment of the sulfur chains to the sulfated carbon edge is thermodynamically favorable across all chain lengths studied. The S1 unit (chain length = 1) exhibits the strongest interaction with the edge. This strong anchoring arises partly from the reference energy. An isolated S atom in a vacuum supercell is comparatively high in energy, making the edge-bound configuration substantially more stable. The optimized structure shows S1 forming two short S–S edge contacts with edge sulfur atoms (~2.8 Å), characteristic of a chemisorption-like interaction.

For S2 (chain length = 2), the anchoring is weakened by both geometric and reference-state effects. When a second sulfur is added on the vacuum side, the system adopts an S2–S1 edge arrangement. Because the terminal sulfur (S2) has no additional bonding partner extending into the vacuum, it interacts solely with S1. This causes the S2–S1 intrachain bond to strongly compete with the S1–S_edge_ bond, pulling S1 slightly away from the edge and reducing the effective anchoring. In addition, the reference state for this comparison, a free S2 molecule in vacuum, is already relatively stable, which further lowers the net anchoring strength. As a result of these combined factors, S2 exhibits a weaker but still negative anchoring energy (≈ –4 kT), meaning that the small radical will remain in the vicinity of the sulfated edge.

For longer chains (chain length = 3 to 5), we observe that the S1–S_edge_ bond recovers and stabilizes around ~2.95 Å, with anchoring energies in the range of –0.14 to –0.22 eV. Small fluctuations arise from differences in the conformations adopted by the free chains in the vacuum supercell, which slightly modify their reference energies. Despite these variations, all values remain negative, demonstrating that sulfur chains of lengths 1 through 5 thermodynamically prefer to anchor to the sulfated carbon edge rather than remain free.

Finally, Table S11 summarizes the energetics of Na-driven sulfur extraction from edge sites. All energies are reported relative to the convex hull of the most stable C–S configurations. Positive values show that Na extraction of edge-bound sulfur is energetically disfavored, reinforcing the confinement effect.


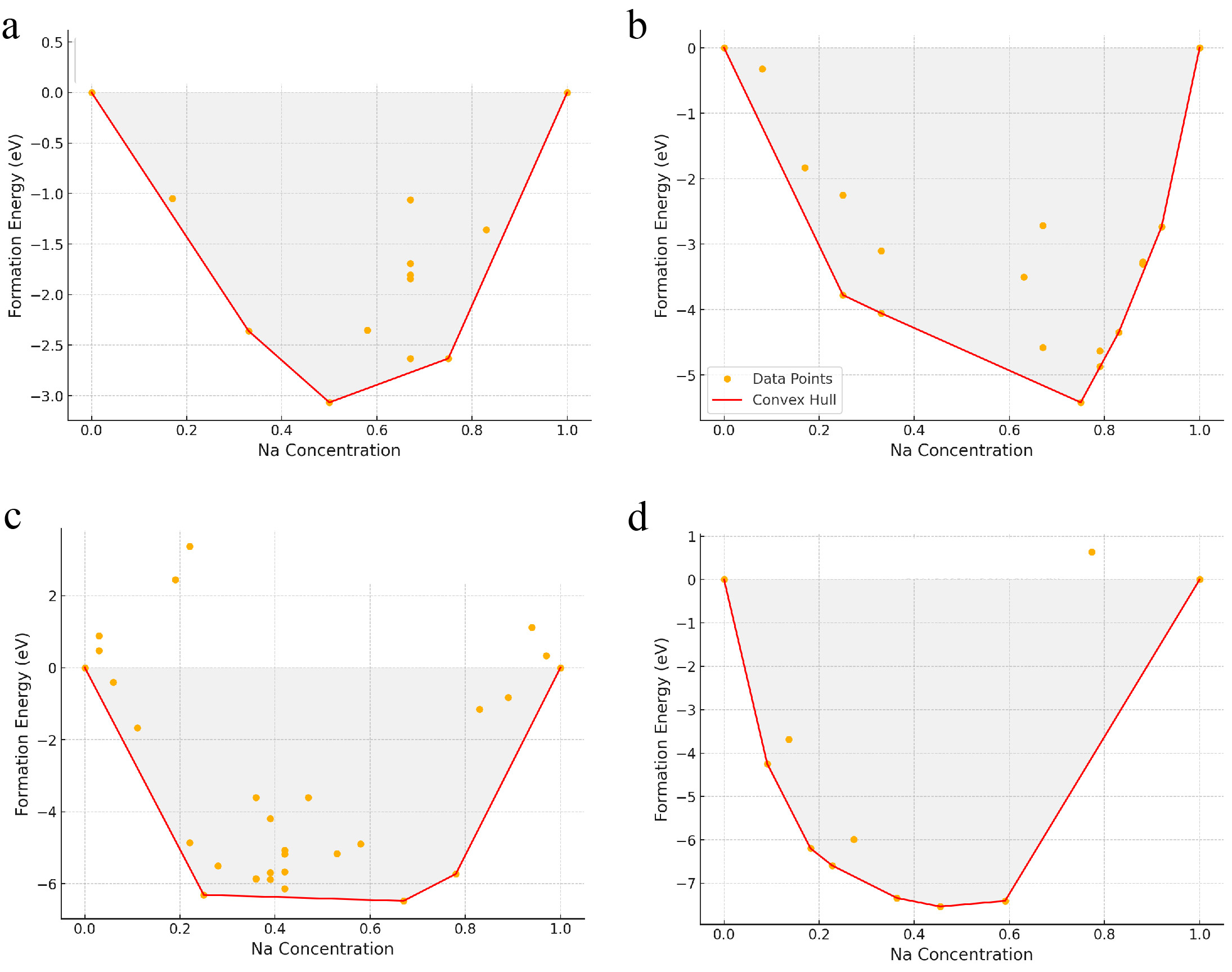


**Figure S30.** Convex hull diagram and voltage profile for the a) iso-4S-chain b) inter-8S-chain c) 2-inter-8S-chain d) 2-inter-2S-chain models.

The sulfur utilization of cathodes in metal-sulfur batteries is an important metric to evaluate the efficiency of the active material in contributing to the overall capacity of the battery. It can be calculated by normalizing the capacity to the amount of sulfur in the active material and comparing it to the theoretical specific capacity of sulfur (C_Theo Sulfur_ = 1675 mAh g^-^¹).^[65-66]^ The formula for sulfur utilization is given by:

$$Sulfur utilization \%=\frac{C_{Sulfur}}{C_{Theo Sulfur}}$$

The carbon matrix plays a dual role in the investigated sulfur-carbons, not only supporting the structural stability, but also contributing directly to the overall capacity. A systematic approach is used to differentiate the contributions of the sulfur (C_Sulfur_) and the carbon matrix (C_Carbon_) to the total capacity (C_Total_). After activation cycles and stable electrochemical performance, the sulfur-carbons are first discharged to a potential of 0.01 V, and then charged to a higher cut-off potential. The high cut-off potential is gradually increased from 0.25 to 2.5 V (Figure S31). During this increase, a linear slope of charge-discharge profiles is observed, due to the adsorption of Na-ions on the carbon surface and defects sites. The increase in capacity at higher cut-off potentials remains linear up to 1.5 V, beyond which a significant increase in capacity is observed. The appearance of a plateau in the GCD profile reflects the initiation of the redox processes related to the sulfur conversion. By analyzing the capacity contributions before and after the start of sulfur conversion, the respective roles of the carbon matrix and sulfur can be deconvoluted. To calculate the capacity of sulfur, the sulfur contribution to the total capacity is then normalized by the sulfur content (Sulfur%) in the active material. Finally, this value is divided by the theoretical specific capacity of sulfur to calculate the sulfur utilization.

$$Sulfur utilization (\%)=\frac{C_{Total}-C_{Carbon}}{C_{Theo Sulfur}\cdot Sulfur\%}$$

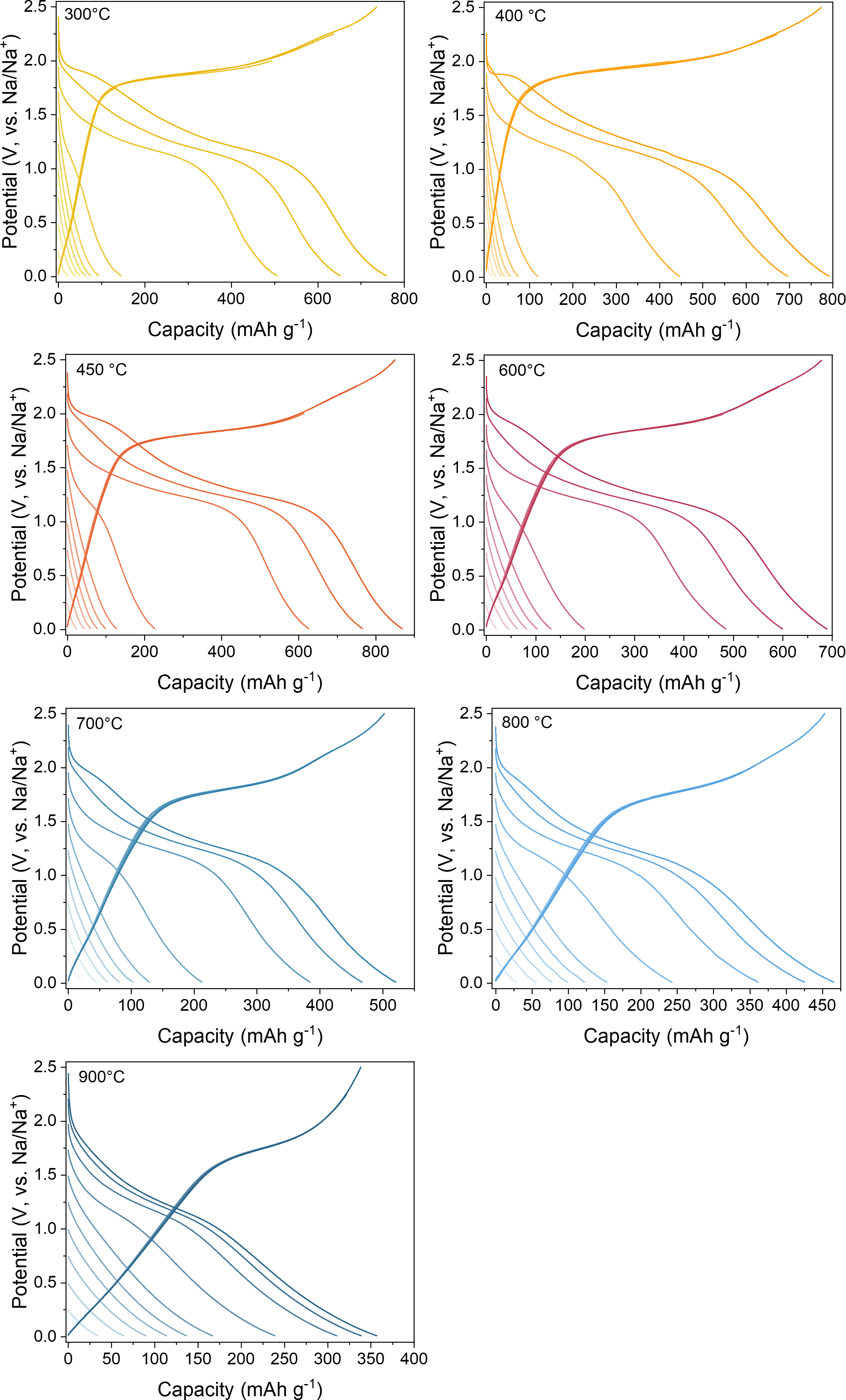


**Figure S31.** GCD measurements at 0.1 C with a 0.25 V step-wise increase of the high cut-off potential for sulfur-carbons condensed between 300 and 900 °C, showing minor sulfur-contribution till a potential of 1.5 V.

Calculating the sulfur utilization for sulfur-carbons thermally condensed at 800 and 900 °C, may underestimate the contribution of the carbon matrix due to its high surface area and numerous active sites that facilitate Na-ion adsorption at elevated potentials. To mitigate this, the sulfur-carbons prepared at these condensation temperatures were cycled within constrained potential ranges where possible sulfur conversion will be irreversible, thereby excluding sulfur contribution (Figure S32). These potential ranges were determined for each sulfur-carbon through analysis of the sulfur redox peaks in CV measurements. This approach provides a more accurate estimation of contribution from the carbon matrix to the total capacity.


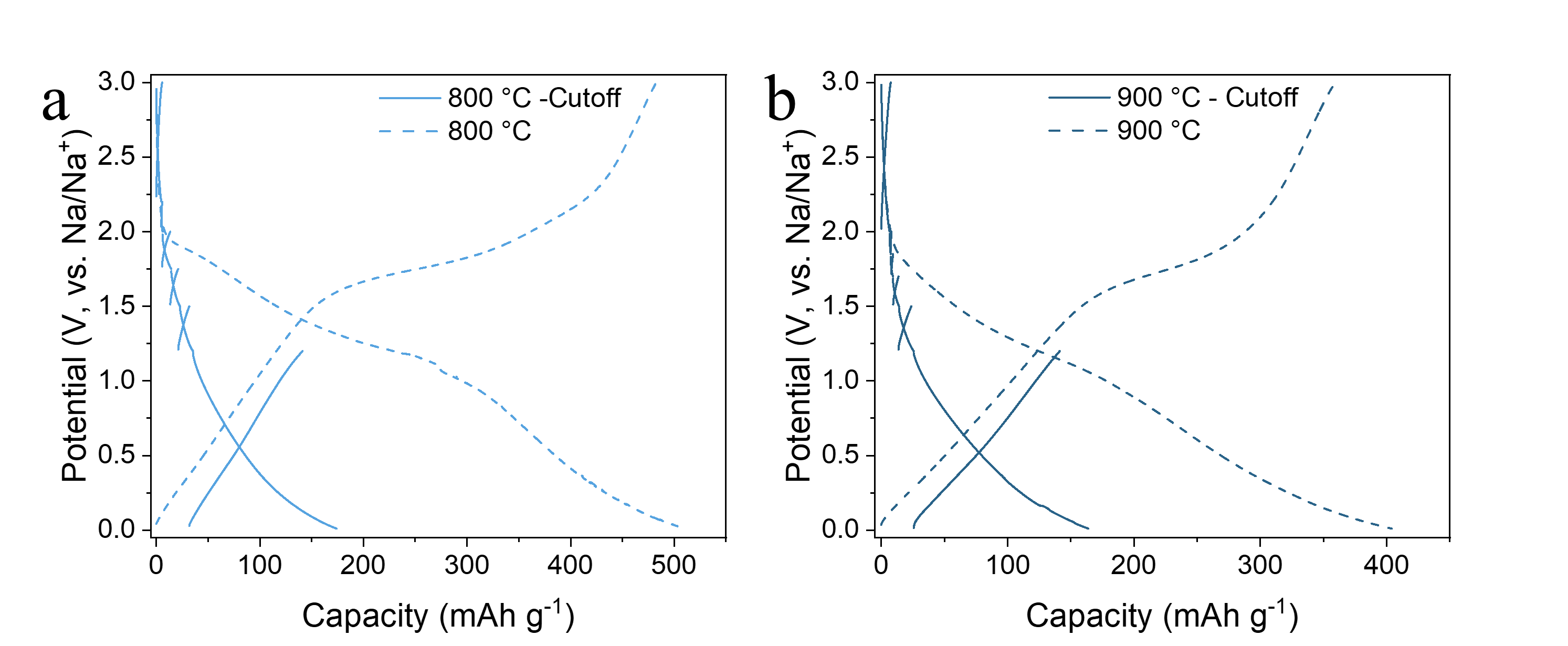


**Figure S32.** Sulfur-carbons condensed at 800 and 900 °C cycled in constrained potential ranges at 0.1 C to exclude sulfur conversion, allowing for a more accurate calculation of the contribution of the carbon matrix to the total capacity.

Typically, sulfur conversion in Na-S battery systems occurs between 0.75 and 2.50 V.^[67]^ However, gradually increasing the low-cutoff potential from 0.01 to 0.75 V results in a noticeable reduction in the current response of the sulfur oxidation peak at 1.8 V, suggesting that the redox processes associated with sulfur conversion are influenced over a broader potential range in sulfur-carbons cathodes compared to conventional cathodes (Figure S33). This behavior can be attributed to the confinement of sulfur in the sulfur-carbons, which requires lower potentials to fully access all sulfur species. The confinement restricts the availability of sulfur for conversion at higher potentials, necessitating a broader potential window for full utilization. This behavior is observed for all sulfur-carbon condensed between 300 and 900 °C. The reduction in the current response is more pronounced at lower condensation temperatures and decreases with increasing condensation temperature. This is trend is related to the structural changes occurring in the sulfur-carbons as the condensation temperature rises. At lower condensation temperatures, sulfur species are confined within an amorphous carbon matrix, making them less accessible for conversion at higher potentials. As the condensation temperature increases, the carbon matrix becomes more ordered and graphitic, improving the accessibility of sulfur species and reducing the necessity for lower low-cutoff potentials to achieve high sulfur utilization. This transition leads to a more efficient sulfur conversion process at higher temperatures, as the sulfur-carbons become more suited for Na-ion transport and sulfur utilization.


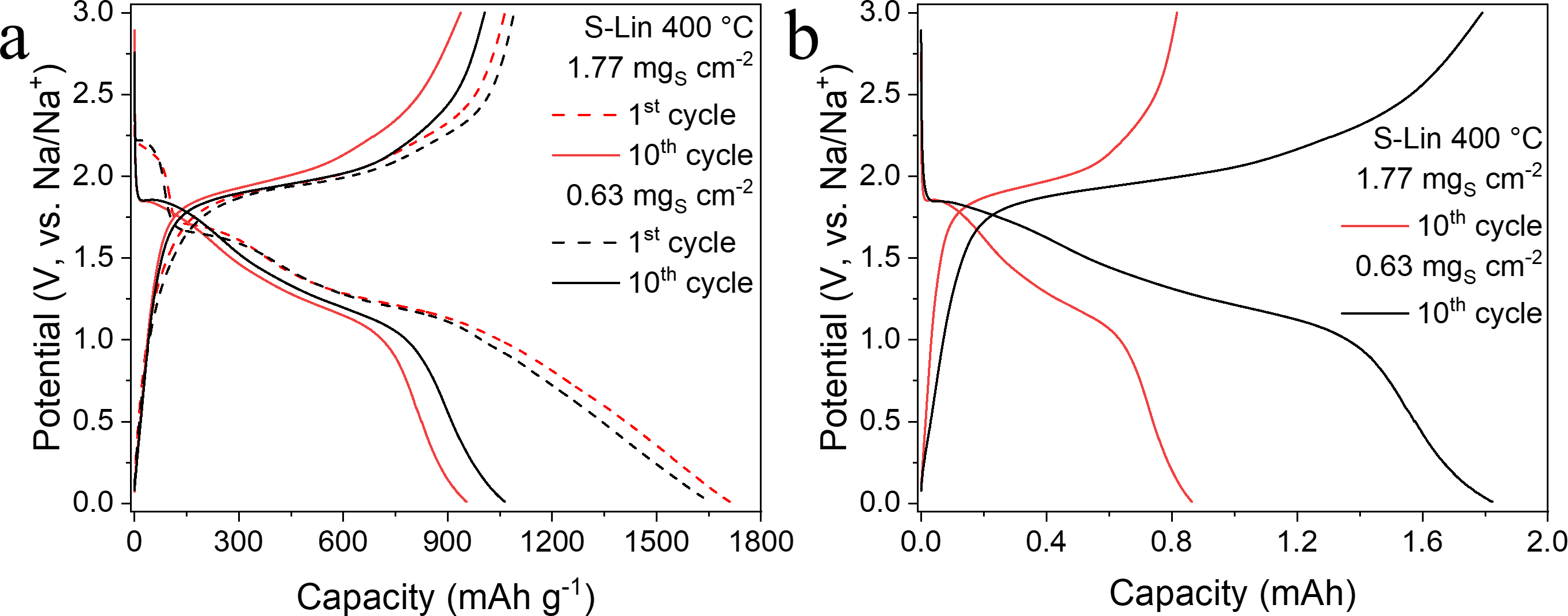


**Figure S33.** GCD measurements at 0.1 C of sulfur-carbon condensed at 400 °C with prepared electrodes with normal and high sulfur loadings with a) normalized and b) absolute capacity.


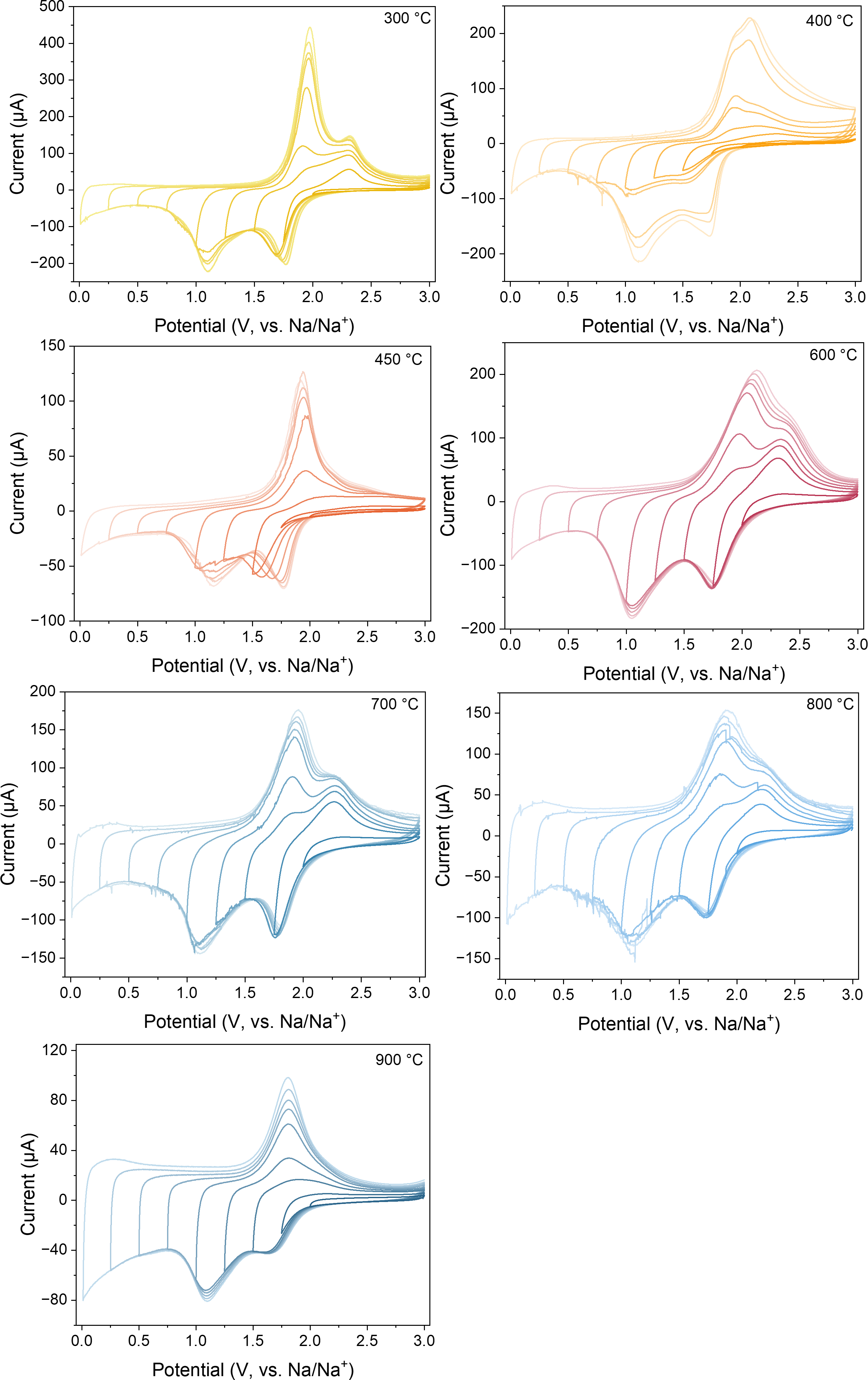


**Figure S34.** CV curves (scan rate 0.1 mV s^-1^) of sulfur-carbons condensed between 300 and 900 °C, with step-wise increasing low-cutoff potential, showing a decrease in current response as the low-cutoff potential is increased, even at potentials below typical sulfur conversion potential ranges.

[1] M. Teubner, R. Strey, *J. Chem. Phys.* **1987**, *87*, 3195-3200.

[2] P. Debye, A. M. Bueche, *J. Appl. Phys.* **1949**, *20*, 518-525.

[3] D. Saurel, J. Segalini, M. Jauregui, A. Pendashteh, B. Daffos, P. Simon, M. Casas-Cabanas, *Energy Storage Mater.* **2019**, *21*, 162-173.

[4] S. Wei, S. Xu, A. Agrawral, S. Choudhury, Y. Lu, Z. Tu, L. Ma, L. A. Archer, *Nat. Commun.* **2016**, *7*, 11722.

[5] S. Li, Z. Zeng, J. Yang, Z. Han, W. Hu, L. Wang, J. Ma, B. Shan, J. Xie, *ACS Appl. Energy Mater.* **2019**, *2*, 2956-2964.

[6] X. Xu, D. Zhou, X. Qin, K. Lin, F. Kang, B. Li, D. Shanmukaraj, T. Rojo, M. Armand, G. Wang, *Nat. Commun.* **2018**, *9*, 3870.

[7] B.-W. Zhang, T. Sheng, Y.-D. Liu, Y.-X. Wang, L. Zhang, W.-H. Lai, L. Wang, J. Yang, Q.-F. Gu, S.-L. Chou, H.-K. Liu, S.-X. Dou, *Nat. Commun.* **2018**, *9*, 4082.

[8] B.-W. Zhang, T. Sheng, Y.-X. Wang, S. Chou, K. Davey, S.-X. Dou, S.-Z. Qiao, *Angew. Chem. Int. Ed.* **2019**, *58*, 1484-1488.

[9] Q. Guo, S. Li, X. Liu, H. Lu, X. Chang, H. Zhang, X. Zhu, Q. Xia, C. Yan, H. Xia, *Adv. Sci.* **2020**, *7*, 1903246.

[10] C. Ye, Y. Jiao, D. Chao, T. Ling, J. Shan, B. Zhang, Q. Gu, K. Davey, H. Wang, S. Z. Qiao, *Adv. Mater.* **2020**, *32*, e1907557.

[11] Z. Yan, J. Xiao, W. Lai, L. Wang, F. Gebert, Y. Wang, Q. Gu, H. Liu, S.-L. Chou, H. Liu, S.-X. Dou, *Nat. Commun.* **2019**, *10*, 4793.

[12] H. Liu, W. Pei, W.-H. Lai, Z. Yan, H. Yang, Y. Lei, Y.-X. Wang, Q. Gu, S. Zhou, S. Chou, H. K. Liu, S. X. Dou, *ACS Nano* **2020**, *14*, 7259-7268.

[13] D. Zhao, S. Jiang, S. Yu, J. Ren, Z. Zhang, S. Liu, X. Liu, Z. Wang, Y. Wu, Y. Zhang, *Carbon* **2023**, *201*, 864-870.

[14] A. Ghosh, S. Shukla, M. Monisha, A. Kumar, B. Lochab, S. Mitra, *ACS Energy Lett.* **2017**, *2*, 2478-2485.

[15] F. Zheng, Y. Zhang, G. Ding, Y. Xiao, L. Wei, J. Su, C. Wang, Q. Chen, H. Wang, *Adv. Funct. Mater.* **2023**, *34*, 2310598.

[16] Samriddhi, A. Patel, A. Tiwari, S. P. Singh, V. Yadav, R. K. Tiwari, R. K. Singh, *J. Energy Storage* **2024**, *99*, 113260.

[17] Y. X. Wang, J. Yang, W. Lai, S. L. Chou, Q. F. Gu, H. K. Liu, D. Zhao, S. X. Dou, *J. Am. Chem. Soc.* **2016**, *138*, 16576-16579.

[18] N. Wang, Y. Wang, Z. Bai, Z. Fang, X. Zhang, Z. Xu, Y. Ding, X. Xu, Y. Du, S. Dou, G. Yu, *Energy Environ. Sci.* **2020**, *13*, 562-570.

[19] X. Ye, J. Ruan, Y. Pang, J. Yang, Y. Liu, Y. Huang, S. Zheng, *ACS Nano* **2021**, *15*, 5639-5648.

[20] B. Guo, W. Du, T. Yang, J. Deng, D. Liu, Y. Qi, J. Jiang, S. J. Bao, M. Xu, *Adv. Sci.* **2020**, *7*, 1902617.

[21] A. Y. S. Eng, Y. Wang, D. T. Nguyen, S. Y. Tee, C. Y. J. Lim, X. Y. Tan, M. F. Ng, J. Xu, Z. W. Seh, *Nano Lett.* **2021**, *21*, 5401-5408.

[22] W. Du, W. Gao, T. Yang, B. Guo, L. Zhang, S. J. Bao, Y. Chen, M. Xu, *J. Colloid Interface Sci.* **2020**, *565*, 63-69.

[23] J. Yan, W. Li, R. Wang, P. Feng, M. Jiang, J. Han, S. Cao, Z. Zhang, K. Wang, K. Jiang, *ACS Energy Lett.* **2020**, *5*, 1307-1315.

[24] L. Wang, X. Chen, S. Li, J. Yang, Y. Sun, L. Peng, B. Shan, J. Xie, *J. Mater. Chem. A* **2019**, *7*, 12732-12739.

[25] Z. Qiang, Y.-M. Chen, Y. Xia, W. Liang, Y. Zhu, B. D. Vogt, *Nano Energy* **2017**, *32*, 59-66.

[26] Y. Hao, X. Li, X. Sun, C. Wang, *ChemistrySelect* **2017**, *2*, 9425-9432.

[27] Y.-M. Chen, W. Liang, S. Li, F. Zou, S. M. Bhaway, Z. Qiang, M. Gao, B. D. Vogt, Y. Zhu, *J. Mater. Chem. A* **2016**, *4*, 12471-12478.

[28] X. Huo, Y. Liu, R. Li, J. Li, *Ionics* **2019**, *25*, 5373-5382.

[29] X. M. Zhao, Y. W. Yan, X. X. Ren, L. Chen, S. D. Xu, S. B. Liu, X. M. Wang, D. Zhang, *ChemElectroChem* **2019**, *6*, 1229-1234.

[30] Q. Wu, W. Zhang, M. Qin, W. Zhong, H. Yan, H. Zhu, S. Cheng, J. Xie, *Nano Energy* **2024**, *129*, 110049.

[31] F. Zheng, Y. Zhang, Z. Li, G. Yao, L. Wei, C. Wang, Q. Chen, H. Wang, *Nature Communications* **2025**, *16*, 4372.

[32] J. Luo, K. Wang, Y. Qian, P. Wang, H. Yuan, O. Sheng, B. Li, H. Wang, Y. Wang, Y. Liu, J. Nai, X. Tao, W. Li, *Nano Energy* **2023**, *118*, 108958.

[33] T. Mei, X. Li, X. Lin, L. Bai, M. Xu, Y. Qi, *Advanced Functional Materials* **2025**, *35*, 2418126.

[34] M. C. Biesinger, *Applied Surface Science* **2022**, *597*, 153681.

[35] T. L. Barr, S. Seal, *Journal of Vacuum Science & Technology A* **1995**, *13*, 1239-1246.

[36] P. Swift, *Surface and Interface Analysis* **1982**, *4*, 47-51.

[37] G. Beamson, D. R. Briggs, **1992**.

[38] J. Riga, J. J. Verbist, *Journal of the Chemical Society, Perkin Transactions 2* **1983**, 1545-1551.

[39] B. J. Lindberg, K. Hamrin, G. Johansson, U. Gelius, A. Fahlman, C. Nordling, K. Siegbahn, *Physica Scripta* **1970**, *1*, 286.

[40] P. E. Laibinis, G. M. Whitesides, D. L. Allara, Y. T. Tao, A. N. Parikh, R. G. Nuzzo, *Journal of the American Chemical Society* **1991**, *113*, 7152-7167.

[41] C. D. Bain, H. A. Biebuyck, G. M. Whitesides, *Langmuir* **1989**, *5*, 723-727.

[42] N. L. Kapuge Dona, C. P. Maladeniya, R. C. Smith, *Eur. J. Org. Chem.* **2024**, *27*.

[43] J. Sharma, P. A. Champagne, *Chemistry* **2023**, *29*, e202203906.

[44] A. D. Buckingham, R. L. Disch, *Proc. R. Soc. Lond. A* **1997**, *273*, 275-289.

[45] M. Thommes, K. Kaneko, A. V. Neimark, J. P. Olivier, F. Rodriguez-Reinoso, J. Rouquerol, K. S. W. Sing, *Pure and Applied Chemistry* **2015**, *87*, 1051-1069.

[46] L. M. Anovitz, D. R. Cole, *Rev. Mineral. Geochem.* **2015**, *80*, 61-164.

[47] Y. Xia, C. Wang, R. Li, M. Fukuto, B. D. Vogt, *Langmuir* **2018**, *34*, 8767-8776.

[48] S. J. Rettig, J. Trotter, *Acta Crystallographica Section C* **1987**, *43*, 2260-2262.

[49] J. F. Watts, J. Wolstenholme, *An Introduction to Surface Analysis by XPS and AES*, **2019**.

[50] Z. Xu, S. Gu, Z. Sun, D. Zhang, Y. Zhou, Y. Gao, R. Qi, W. Chen, *Environ. Sci. Pollut. Res. Int.* **2020**, *27*, 11012-11025.

[51] M. Fantauzzi, B. Elsener, D. Atzei, A. Rigoldi, A. Rossi, *RSC Advances* **2015**, *5*, 75953-75963.

[52] J. Riga, J. J. Verbist, *J. Chem. Soc. Perkin Trans. 2* **1983**.

[53] B. J. Lindberg, K. Hamrin, G. Johansson, U. Gelius, A. Fahlman, C. Nordling, K. Siegbahn, *Phys. Scr.* **1970**, *1*, 286-298.

[54] P. E. Laibinis, G. M. Whitesides, D. L. Allara, Y. T. Tao, A. N. Parikh, R. G. Nuzzo, *J. Am. Chem. Soc.* **1991**, *113*, 7152-7167.

[55] C. D. Bain, H. A. Biebuyck, G. M. Whitesides, *Langmuir* **2002**, *5*, 723-727.

[56] X. Liang, C. Hart, Q. Pang, A. Garsuch, T. Weiss, L. F. Nazar, *Nat. Commun.* **2015**, *6*, 5682.

[57] S. R. Kelemen, G. N. George, M. L. Gorbaty, *Fuel* **1990**, *69*, 939-944.

[58] C. E. Mixan, J. B. Lambert, *J. Org. Chem.* **2002**, *38*, 1350-1353.

[59] L. D. Setiawan, H. Baumann, D. Gribbin, *Surf. Interface Anal.* **2004**, *7*, 188-195.

[60] A. Lachkar, A. Selmani, E. Sacher, *Synth. Met.* **1995**, *72*, 73-80.

[61] Y. Lu, Z. Dong, P. Wang, H.-B. Zhou, in *Thiophenes* (Ed.: J. A. Joule), Springer International Publishing, Cham, **2015**, pp. 227-293.

[62] F. F. Roman, J. L. Diaz de Tuesta, A. M. T. Silva, J. L. Faria, H. T. Gomes, *Catal.* **2021**, *11*.

[63] A. Y. S. Eng, D.-T. Nguyen, V. Kumar, G. S. Subramanian, M.-F. Ng, Z. W. Seh, *J. Mater. Chem. A* **2020**, *8*, 22983-22997.

[64] A. Vasileiadis, Y. Li, Y. Lu, Y. S. Hu, M. Wagemaker, *ACS Appl. Energy Mater.* **2023**, *6*, 127-140.

[65] Z. C. Yan, L. F. Zhao, Y. X. Wang, Z. Q. Zhu, S. L. Chou, *Adv. Funct. Mater.* **2022**, *32*, 2205622.

[66] E. Senokos, H. Au, E. O. Eren, T. Horner, Z. Song, N. V. Tarakina, E. B. Yilmaz, A. Vasileiadis, H. Zschiesche, M. Antonietti, P. Giusto, *Small* **2024**, *20*, e2407300.

[67] Y. X. Wang, B. W. Zhang, W. H. Lai, Y. F. Xu, S. L. Chou, H. K. Liu, S. X. Dou, *Advanced Energy Materials* **2017**, *7*.
